# A DNA damage-activated kinase phosphorylates a transcriptional repressor to control bacterial immune pathway expression

**DOI:** 10.1038/s44318-026-00831-y

**Published:** 2026-06-09

**Authors:** Lydia R Chambers, Phoolwanti Rani, Ryan K Min, Elizabeth Villa, Kevin D Corbett

**Affiliations:** 1 Department of Biochemistry and Molecular Biophysics, University of California San Diego, La Jolla, CA 92093 USA; 2 Department of Molecular Biology, University of California San Diego, La Jolla, CA 92093 USA; 3 Howard Hughes Medical Institute, University of California San Diego, La Jolla, CA 92093 USA; 4 Department of Cellular and Molecular Medicine, University of California San Diego, La Jolla, CA 92093 USA

**Keywords:** Chromatin, Transcription & Genomics, Microbiology, Virology & Host Pathogen Interaction, Structural Biology

## Abstract

Bacteria encode numerous stress-response pathways that protect their hosts against both internal and external threats. A key question is how these pathways are regulated, especially anti-phage immune pathways that mediate host-cell killing. Here, we identify two proteins termed CapK and CapS that are encoded upstream of diverse immune operons, and regulate these operons’ expression in response to DNA damage. CapK resembles bacterial anti-sigma factor kinases, and CapS resembles STAS-domain antagonists of these proteins. CapS is a DNA-binding transcriptional repressor, and phosphorylation of CapS by CapK results in dissociation of a CapS homodimer and de-repression of transcription. The CapK kinase is directly activated by single-stranded DNA generated as a byproduct of DNA repair. Finally, we show that CapK and CapS-like proteins have been co-opted into an anti-phage toxin-antitoxin system with a VapC-like protein, where they similarly respond to DNA damage to activate VapC nuclease activity. Overall, our results reveal how a kinase-substrate pair can regulate expression of an adjacent operon in response to DNA damage, and highlight the modularity of immune and other stress-response pathways.

## Synopsis

Regulation of bacterial anti-phage immune pathways that mediate host cell killing need to be tightly regulated. This work identifies a pair of proteins, CapK and CapS, that together control expression of adjacently-encoded immune pathway in response to a universal stress signal, DNA damage.

- The kinase CapK and the STAS-domain transcription factor CapS are encoded adjacent to bacterial immune pathways.
- CapS forms a homodimer that binds the promoter of the adjacent immune pathway, repressing transcription.
- Upon DNA damage, CapK is activated and phosphorylates the STAS domain of CapS.
- Phosphorylation causes dissociation of the CapS homodimer, disrupting DNA binding and de-repressing the bacterial immune pathway.
- CapK and CapS-like proteins have been co-opted into a separate toxin-antitoxin system, where they control the activity of a VapC-like RNase toxin.

## Abstract (web summary)

CapK and CapS are encoded in bacterial genomes adjacent to known and putative immune pathways and regulate their expression in response to the universal stress signal DNA damage.

## Graphical abstract

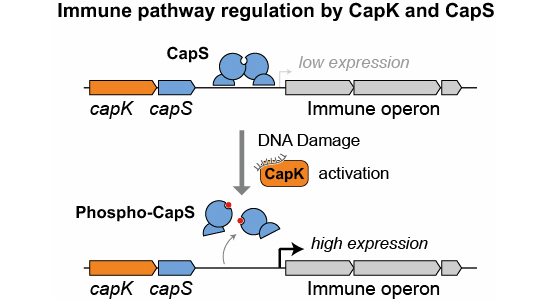

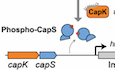

## Introduction

Under the pressure of environmental stress and the constant threat of phage infection, bacteria have evolved a large repertoire of immune pathways with diverse mechanisms for infection sensing and host protection (Bernheim and Sorek, 2020; Hampton et al, 2020). In the past decade, hundreds of distinct bacterial immune pathways have been identified, which are often encoded in clusters termed “defense islands” in bacterial genomes. Immune pathways typically function either by directly targeting foreign DNA (as in Restriction-Modification (R-M) and CRISPR-Cas systems) or by causing growth inhibition and/or cell death to limit phage replication (broadly termed abortive infection) (Gao et al, 2020; Johnson et al, 2022; Lopatina et al, 2020; Tamulaitiene et al, 2024). Abortive infection pathways in particular must be tightly regulated to prevent aberrant activation, which could lead to toxicity in the absence of infection.

Many anti-phage immune pathways are regulated at the level of transcription and/or translation, with expression of these pathways induced by external or internal stress signals. For example, quorum sensing is utilized by some CRISPR-Cas systems, with these pathways’ expression repressed at low cell density and de-repressed at high cell density (Høyland-Kroghsbo et al, 2017; Patterson et al, 2016). Many R-M systems encode a controller (C) protein that enables fine-tuned control over the relative expression timing of these systems’ methylase and endonuclease components (Negri et al, 2021). Additionally, two families of DNA damage-responsive transcriptional regulators have been identified that regulate the expression of adjacently-encoded immune operons, including BREX, Pycsar, CBASS, and DISARM. These include CapW/BrxR, which represses transcription until it binds single-stranded DNA (ssDNA); and CapH+CapP, whose CapP protease is activated upon ssDNA binding and cleaves the DNA-binding repressor CapH (Lau et al, 2022; Blankenchip et al, 2022; Blankenchip and Corbett, 2024; Picton et al, 2022; Luyten et al, 2022). These two families of DNA damage activated regulators highlight the importance of direct control of gene expression in immune pathways.

Here, we identify and characterize a third family of immune pathway-associated, DNA damage activated transcriptional regulators termed CapK+CapS. We identify *capK+capS* genes associated with diverse known and putative bacterial immune pathways, including CBASS, Bil (bacterial ISG15-like), and Bub (bacterial ubiquitination-like). We find that CapS is a DNA-binding transcriptional repressor, which is phosphorylated by CapK upon DNA damage to de-repress an adjacently-encoded immune operon. Finally, we identify standalone operons encoding CapK and CapS-like proteins plus a VapC-like RNase toxin, and show that these operons control VapC activation through a similar DNA damage sensing mechanism. Overall, our findings emphasize the prevalence and importance of immune pathway regulation at the transcriptional level, and the central role of DNA damage in activation of immune pathway expression.

## Results

### A predicted kinase-substrate gene pair associated with bacterial immune operons

**Figure 1:**
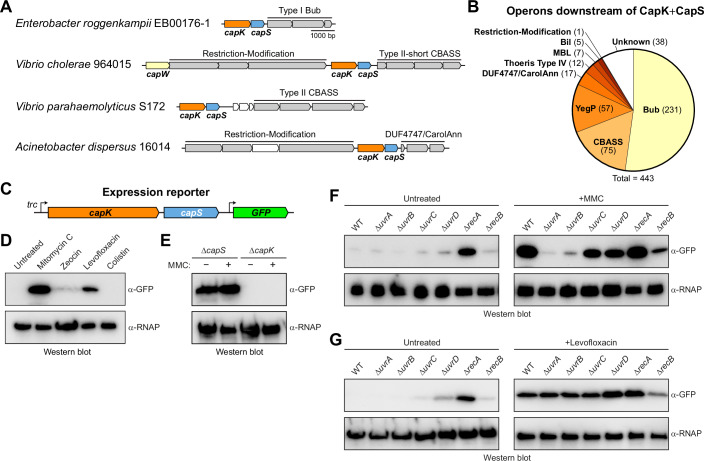
Identification of CapK and CapS. (**A**) Operon schematics of four bacterial immune pathways associated with *capK* (orange) and *capS* (blue) genes. Immune pathways are shown in gray and labeled; unknown genes are shown in white. See Dataset EV2 for the full list and accession numbers. (**B**) Classification of 443 identified *capKS*-associated operons. (**C**) Schematic of the GFP expression reporter derived from an *E. roggenkampii* strain EB00176-1 CBASS-associated *capKS* locus (see panel (**A**)). An IPTG-inducible *trc* promoter is positioned upstream of *capK*, and the CBASS operon is replaced by the coding sequence for GFP (green). For some experiments (noted in figure legends), an N-terminal FLAG-tag was fused to the *capS* gene. (**D**) Anti-GFP western blot showing induction of GFP expression in the presence of different small molecules (concentrations noted in Materials and methods), using the GFP expression reporter expressing FLAG-CapS. α-RNAP: loading control blot for RNA polymerase subunit RpoS. Western blots in panels (**D**–**G**) are representative of at least three independent trials. (**E**) Anti-GFP western blot showing the effect of deleting *capS* or *capK* in the GFP expression reporter, in the presence or absence of mitomycin C (MMC). (**F**) Anti-GFP western blot showing GFP expression in the presence or absence of mitomycin C (MMC) in the indicated single-gene knockout *E. coli* strains. This experiment used the GFP expression reporter expressing FLAG-CapS. (**G**) Anti-GFP western blot showing GFP expression in the presence or absence of levofloxacin in the indicated single-gene knockout *E. coli* strains. This experiment used the GFP expression reporter expressing FLAG-CapS. Source data are available online for this figure.

We recently defined a family of putative bacterial immune pathways termed Bub (bacterial ubiquitination-like), and identified hundreds of Bub operons in diverse bacteria (Gong et al, 2025; Ye et al, 2026). By manually inspecting these operons’ gene neighborhoods, we found that 219 out of 544 identified Bub operons are associated with either CapW (CBASS-associated protein, WYL domain) or CapH+CapP (CBASS-associated proteins, Helix-turn-helix and Peptidase), both of which control the expression of their adjacent operon in response to DNA damage (Blankenchip et al, 2022; Blankenchip and Corbett, 2024; Lau et al, 2022). Further inspection revealed that 59 of 544 Bub operons were associated with two genes of unknown function, one of which encodes a predicted HTH (helix-turn helix), STAS (sulfate transporter and anti-sigma factor antagonist) (Aravind and Koonin, 2000), and GHKL (DNA Gyrase, histidine kinase, MutL) (Dutta and Inouye, 2000) ATPase/kinase domains, and the second of which encodes a predicted STAS domain followed by a wHTH (winged helix-turn-helix) domain (Fig. 1A; Dataset EV1). These two genes’ location adjacent to the Bub operons’ promoters suggests that they may regulate transcription in a manner similar to CapW and CapH+CapP. Following a similar naming convention, we named the HTH-STAS-GHKL kinase protein CapK (Kinase), and the STAS-wHTH protein CapS (STAS domain). Reflecting the fact that CapK + CapS was identified adjacent to Bub immune pathways rather than CBASS, we propose that “Cap” for transcriptional regulators (including CapW, CapH + CapP, and CapK+CapS) henceforth stand for “Controller of associated/adjacent pathways”.

We performed comprehensive BLAST searches in the Integrated Microbial Genomes (IMG) database, and identified 443 homologs of *capK*, all of which are immediately followed by *capS*. In 348 of 443 cases, *capK* and *capS* are followed by a known or predicted bacterial immune operon (Fig. 1B; Dataset EV2), including CBASS (75 cases) (Davies et al, 2012; Cohen et al, 2019; Ye et al, 2020), DUF4747/CarolAnn prophage system (17 cases) (Montgomery et al, 2019; Parma et al, 1992), and Type IV Thoeris (12 cases) (Rousset et al, 2025). We also identified five Bil operons (Millman et al, 2022; Chambers et al, 2024; Hör et al, 2024), 7 MBL operons related to Type II CBASS, and 231 Bub operons with upstream *capK* and *capS*. In 57 cases, *capK* and *capS* were followed by a gene encoding a protein homologous to the uncharacterized *E. coli* protein YegP. Finally, we found that in 88 out of 443 cases, *capK* and *capS* are located immediately downstream of a predicted R-M system (Fig. 1A), suggesting that the two genes may coordinate the immune activities of an upstream R-M system and a downstream immune system like CBASS or Bub.

### CapK and CapS regulate transcription of a downstream immune operon

The location of *capK* and *capS* upstream of diverse immune operons suggests that, like CapW and CapH+CapP, they regulate transcription of the downstream operon in response to stress. To test this idea, we chose a *capK*+*capS* gene pair associated with a Bub operon in *Enterobacter roggenkampii* strain EB00176-1 (Fig. 1A), and created a GFP expression reporter by replacing the Bub operon with a gene encoding GFP (maintaining the intergenic sequence downstream of *capS*; Fig. 1C). We also fused *capS* to an N-terminal FLAG epitope tag, enabling us to track expression of CapS directly. In log-phase *E. coli* cultures in rich media, GFP expression was undetectable. When we exposed cells to different types of stress by adding antibiotics, membrane-disrupting compounds, and DNA-damaging agents, we observed induction of GFP expression after adding the DNA-damaging agent mitomycin C (MMC) or the type II DNA topoisomerase inhibitor levofloxacin (Fig. 1D). We next deleted either *capK* or *capS* in our GFP expression reporter, and found that deletion of *capS* results in constitutive GFP expression, and that deletion of *capK* disrupts MMC-activated expression (Fig. 1E). These data suggest that CapS is a transcriptional repressor for the downstream operon, and that CapK relieves CapS-mediated repression in response to DNA damage.

Mitomycin C damages DNA by creating interstrand crosslinks (Tomasz, 1994). These lesions are predominantly repaired by the UvrABCD nucleotide excision repair (NER) pathway (Truglio et al, 2006). To determine whether the UvrABCD pathway plays a role in CapK+CapS-mediated transcriptional regulation, we tested MMC-dependent GFP reporter expression in *E. coli* strains lacking *uvrA*, *uvrB*, *uvrC*, or *uvrD*, in addition to strains lacking the homologous recombination repair proteins *recA* or *recB*. We found that MMC-dependent GFP expression depends on both *uvrA* and *uvrB*, which act early in the NER pathway to recognize damaged DNA (Fig. 1F). In contrast, GFP expression after exposure to levofloxacin, which generates a range of lesions due to its inhibition of type II DNA topoisomerases, does not depend on *uvrA* or *uvrB* (Fig. 1G). In both MMC- and levofloxacin-treated cells, deletion of the *recB* nuclease reduced GFP expression (Fig. 1F,G). Overall, these data support a model in which CapK and CapS sense a byproduct of DNA repair to activate downstream operon expression.

### CapS binds two sites in the *capK-capS* region

**Figure 2:**
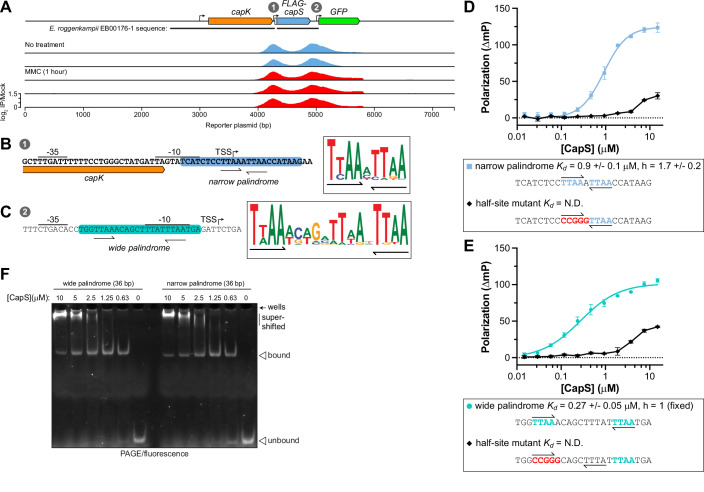
CapS binds two sites in the *capK-capS* region. (**A**) ChIP-Seq results measuring binding of FLAG-CapS to a plasmid encoding the *capK*-*capS* genomic region (660 bp upstream of *capK* to the start codon of the downstream Bub operon) followed by a gene coding for GFP. Data is expressed as the log2 enrichment of FLAG-immunopurified samples over mock-purified samples (log2 IP/Mock), normalized for RPM coverage (see Methods). No-treatment samples are shown in blue, and MMC-treated samples are shown in red. Each profile represents an independent biological replicate; one replicate of the no-treatment condition failed after DNA isolation and was not included. (**B**) Closeup schematic of promoter #1 (upstream of *capS*) as indicated in panel (**A**). *Inset:* Sequence logo from alignment of 100 related *capKS* loci. (**C**) Closeup schematic of promoter #2 (downstream of *capS*) as indicated in panel (**A**). *Inset:* Sequence logo from alignment of 100 related *capKS* loci. The conserved “TTAA” sequence between the two indicated palindrome half-sites is part of the -10 site. (**D**) Fluorescence polarization DNA-binding assay with *E. roggenkampii* CapS and a 24-bp narrow palindrome DNA (light blue squares) or a half-site mutant DNA (black diamonds). Each datapoint is an average of three technical replicates, and error bars indicate the mean ± standard deviation (error bars not shown if they are smaller than the datapoint itself). Data were fit with a cooperative binding model. Fit *K*_d_ and h (Hill coefficient) values are shown at bottom. Data presented were representative of three independent trials. (**E**) Fluorescence polarization DNA-binding assay with *E. roggenkampii* CapS and a 24-bp wide palindrome DNA (light green circles) or a half-site mutant DNA (black diamonds). Each datapoint is an average of three technical replicates, and error bars indicate the mean ± standard deviation (error bars not shown if they are smaller than the datapoint itself). Data were fit with a single-site binding model. Fit *K*_d_ values are shown at bottom. The data presented were representative of three independent trials. (**F**) Electrophoretic mobility shift assay (EMSA) for CapS binding 36-bp dsDNA fragments containing the wide and narrow palindromic sequences. The band labeled “unbound” represents the DNA alone; CapS binding results in the formation of an initial shifted band (marked “bound”) and a super-shifted band close to the wells at the top. Data presented were representative of three independent trials. Source data are available online for this figure.

Our GFP expression reporter assays suggested that CapS acts as a repressor, likely by binding promoter DNA through its predicted wHTH domain. To identify CapS binding sites, we modified our GFP expression reporter to include the entire *E. roggenkampii capK-capS* region, including 660 bp upstream of *capK* and the entire intergenic region between *capS* and the downstream Bub operon. We used ChIP-Seq to measure binding of FLAG-CapS to DNA, in either unperturbed cells or one hour after MMC treatment (Fig. 2A). We detected two strong peaks of CapS-DNA binding: one in the short intergenic region between *capK* and *capS* (peak #1) and a second in the longer intergenic region between *capS* and the downstream GFP gene (peak #2). Unexpectedly, exposure of cells to MMC did not disrupt CapS-DNA binding as measured by ChIP-Seq (Fig. 2A); this likely reflects incomplete removal of CapS from promoter DNA, exacerbated by the dramatic increase in CapS expression upon MMC exposure (see below).

**Figure EV1:**
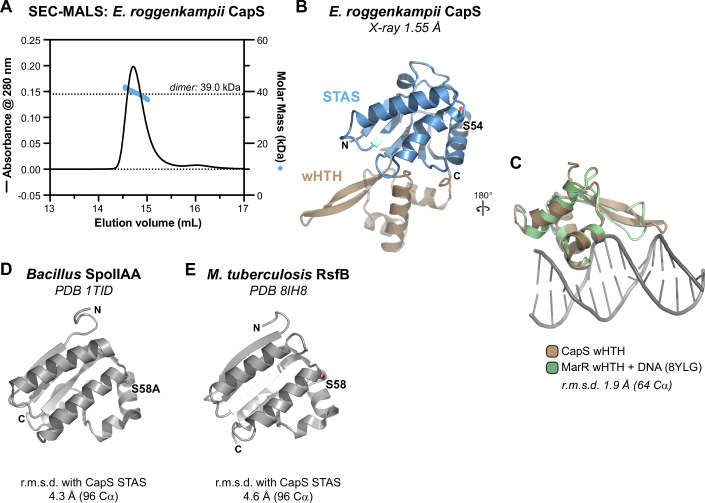
Structure of *E. roggenkampii* CapS. (**A**) Size exclusion chromatography coupled to multi-angle light scattering (SEC-MALS) analysis of purified *E. roggenkampii* CapS. This experiment was performed once. (**B**) Structure of *E. roggenkampii* CapS, with N-terminal STAS domain blue and C-terminal wHTH domain brown. The N- and C-termini of the STAS domain are labeled, and Ser54 is shown as sticks and labeled. (**C**) Overlay of *E. roggenkampii* CapS (brown) with a crystal structure of MarR bound to DNA (green; PDB ID 8YLG) (Song et al, 2024); only the wHTH domains of both proteins are shown. (**D**) Structure of *Geobacillus stearothermophilus* SpoIIAA (from PDB ID 1TID) (Masuda et al, 2004), with Ser58 (mutated to alanine) shown as sticks and labeled. The overall Cα r.m.s.d. (root mean squared deviation) with CapS is 4.3 Å over 96 residues. (**E**) Structure of *Mycobacterium tuberculosis* RsfB (from PDB ID 8IH8; unpublished), with Ser58 shown as sticks and labeled. The overall Cα r.m.s.d. with CapS is 4.6 Å over 96 residues.Source data are available online for this figure.

We used the BPROM server (Solovyev and Salamov, 2011) to identify putative promoter sequences in both the *capK-capS* intergenic region (Fig. 2B) and the *capS*-Bub operon intergenic region (Fig. 2C). Based on our observation that purified *E. roggenkampii* CapS is dimeric in solution and therefore likely binds a symmetric target sequence (Fig. EV1A), we searched for palindromic sequences overlapping these promoters and corresponding to ChIP-Seq peaks #1 and #2. We identified conserved palindromic sequences in both putative promoters with the same half-site sequence (TTAA), but with the half-sites spaced differently in the two promoters. In the *capK-capS* intergenic region (corresponding to peak #1), we identified a “narrow palindrome” with the sequence TTAAaTTAA, where the two half-sites are separated by one base pair (Fig. 2B). In the *capS*-downstream gene region (corresponding to peak #2), we identified a “wide palindrome” with the sequence TTAAacagctttatTTAA, where the two half-sites are separated by ten base pairs (Fig. 2C). We performed fluorescence polarization DNA-binding assays with 24-bp DNA segments containing each palindromic sequence, and found that CapS binds the narrow palindrome with a dissociation constant (*K*_d_) of 0.9  ± 0.1 µM (Fig. 2D), and binds the wide palindrome with a slightly higher affinity (*K*_d_ = 0.27 ± 0.05 µM; Fig. 2E). Demonstrating specificity, CapS did not detectably bind DNAs with one palindromic half-site mutated (Fig. 2D,E). Notably, CapS binding to the narrow palindrome showed significant cooperativity, with a best-fit Hill coefficient (h) of ~1.7 (Fig. 2D). CapS binding to the wide palindrome did not show cooperativity, with a best-fit Hill coefficient of 0.9; a single-site binding model was therefore used to fit this data (Fig. 2E).

Spurred by these results, we next tested longer 36-bp wide and narrow palindromes for CapS binding in an electrophoretic mobility shift assay (EMSA). Both DNAs showed an initial shift with sub-micromolar binding affinity, followed by a super-shift consistent with the binding of additional CapS dimers (Fig. 2F). In this assay, we did not observe a measurable difference in the electrophoretic mobility of the first shifted species, suggesting that in each case one CapS dimer binds DNA initially, followed by additional CapS dimers. Overall, our fluorescence polarization and EMSA assays show that CapS can bind palindromic sequences with a half-site sequence of TTAA, with these half-sites spaced either one base pair apart (“narrow palindrome”) or ten base pairs apart (“wide palindrome”). Moreover, CapS shows cooperative DNA-binding behavior that differs according to the palindromic half-site spacing.

### The structure of CapS reveals two distinct dimer interfaces

**Figure 3:**
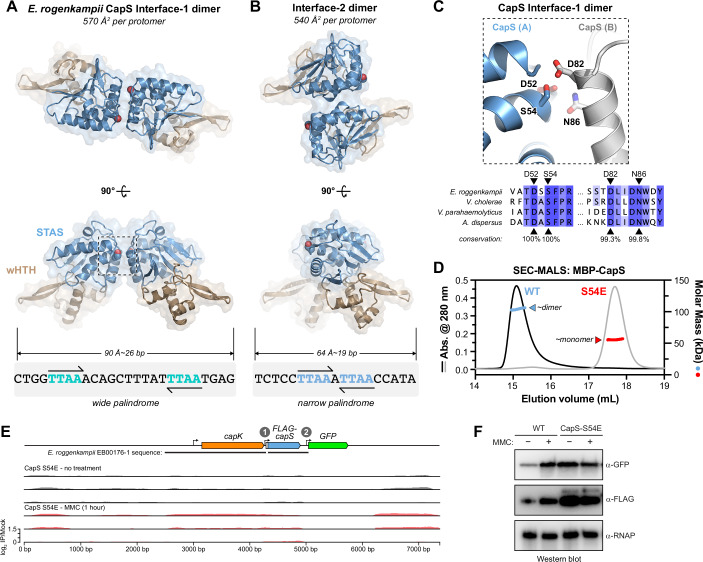
Structure of *E. roggenkampii* CapS. (**A**) *Top:* Top and side views of the *E. roggenkampii* CapS interface-1 dimer, with N-terminal STAS domain (residues 1–111) blue and C-terminal wHTH domain (residues 112–184) brown. Residue Ser54, buried in the dimer interface, is shown as spheres. *Bottom:* “wide palindrome” sequence from promoter #2 (Fig. 2C), to scale with CapS. (**B**) *Top:* Top and side views of the *E. roggenkampii* CapS interface-2 dimer, with N-terminal STAS domain (residues 1–111) blue and C-terminal wHTH domain (residues 112–184) brown. *Bottom:* “narrow palindrome” sequence from promoter #1 (Fig. 2B), to scale with CapS. (**C**) *Top:* Closeup of CapS residue Ser54, which is buried in the CapS interface-1 dimer and is surrounded by several negatively-charged or polar residues from the opposite CapS protomer (gray). *Bottom:* Section of sequence alignment the four CapS proteins shown in Fig. 1A (*E. roggenkampii* (NCBI WP_001567865.1), *V. cholerae* (NCBI WP_046127252.1), *V. parahaemolyticus* (IMG 2662856252), showing conservation of residues in dimer interface 1. (**D**) SEC-MALS of purified *E. roggenkampii* MBP-tagged CapS wild-type (black/blue) or S54E (gray/red). CapS wild-type shows a molecular weight consistent with a homodimer (128.4 kDa), and the S54E mutant shows a molecular weight consistent with a monomer (64.2 kDa). The data presented were representative of three independent trials. (**E**) ChIP-Seq results measuring binding of FLAG-CapS S54E to a plasmid encoding the *capK*-*capS* genomic region, followed by a gene coding for GFP. No-treatment samples are shown in gray, and mitomycin C (MMC)-treated samples are shown in pink. Each profile represents an independent biological replicate. (**F**) Western blots from GFP expression reporter containing FLAG-tagged CapS, in the presence and absence of mitomycin C (MMC) and with wild-type CapS (WT) or the CapS S54E mutant. α-RNAP: loading control blot for RNA polymerase subunit RpoS. The data presented were representative of three independent trials. Source data are available online for this figure.

**Figure EV2:**
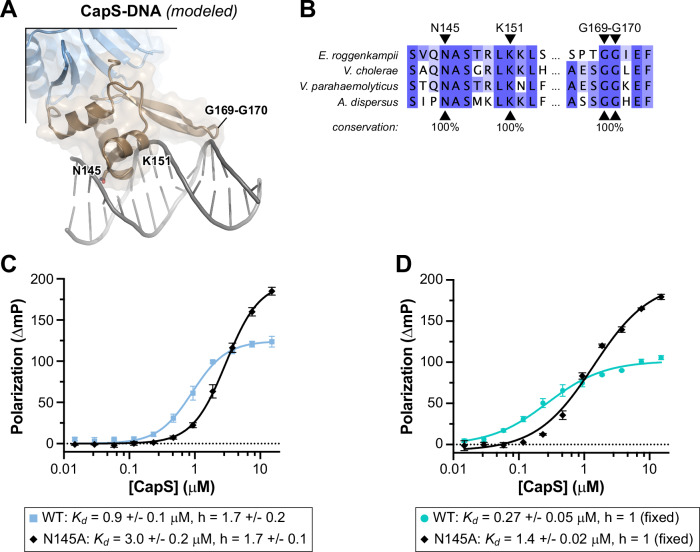
DNA binding by CapS. (**A**) Model of the CapS wHTH domain (brown) bound to DNA (gray), generated by overlaying the CapS wHTH domain with a DNA-bound structure of MarR (PDB ID 8YLG; Fig. EV1C) (Song et al, 2024). Conserved residues predicted to bind DNA (N145, K151, G169, and G170) are shown as sticks and labeled. (**B**) Section of sequence alignment of the four CapS proteins shown in Fig. 1A (*E. roggenkampii* (NCBI WP_001567865.1), *V. cholerae* (NCBI WP_046127252.1), *V. parahaemolyticus* (IMG 2662856252), showing conservation of predicted DNA-binding residues. (**C**) Fluorescence polarization DNA-binding assay with *E. roggenkampii* CapS (wild-type shown in light blue squares, N145A shown in black diamonds) binding a 24-bp narrow palindrome DNA. Each datapoint is an average of three technical replicates, and arrow bars indicate the mean ±  standard deviation (error bars not shown if they are smaller than the datapoint itself). DNA-binding curves were fit with a cooperative binding model (h: Hill coefficient). The data presented in panels (**C**, **D**) were representative of three independent trials. (**D**) Fluorescence polarization DNA-binding assay with *E. roggenkampii* CapS (wild-type shown in light green circles, N145A shown in black diamonds) binding a 24-bp wide palindrome DNA. Each datapoint is an average of three technical replicates, and arrow bars indicate the mean ± standard deviation (error bars not shown if they are smaller than the datapoint itself). DNA-binding curves were fit with a single-site binding model.Source data are available online for this figure.

We next crystallized and determined the structure of *E. roggenkampii* CapS to a resolution of 1.55 Å (Appendix Table S1). The structure reveals that CapS comprises an N-terminal STAS domain and a C-terminal wHTH domain (Fig. 3A,B). Structure comparisons with the DALI server show strong similarity of CapS’s wHTH domain to DNA-binding transcriptional repressors, including ArsR (Viswanathan et al, 2021; Prabaharan et al, 2019) and MarR-family proteins (Conway et al, 2022; Peng et al, 2017; Song et al, 2024) (Fig. EV1B,C). We modeled CapS bound to DNA based on a structure of a DNA-bound MarR transcription factor (Song et al, 2024), and identified several absolutely-conserved residues predicted to contact DNA, including N145, K151, and two glycine residues (169–170) that are positioned at the distal end of the wing motif predicted to bind the DNA minor groove (Fig. EV2A,B). Mutation of N145 to alanine (N145A) reduced CapS’ binding affinity for both the narrow and wide palindrome DNAs by 3-5 fold, supporting a model in which CapS binds DNA via its wHTH domain (Fig. EV2C,D).

Our structure of *E. roggenkampii* CapS contains one protomer in the crystallographic asymmetric unit, but the protein exists as a homodimer in solution (Fig. EV1A) and binds palindromic DNA sequences. We used the PDBePISA server (Krissinel and Henrick, 2007) to identify crystal packing interactions likely to represent the physiologically relevant dimer interface. We identified two major crystal packing interfaces, both involving the STAS domain: interface 1 buries 570 Å^2^ of surface area per protomer (Fig. 3A), and interface 2 buries 540 Å^2^ of surface area per protomer (Fig. 3B). Revisiting the two identified binding sites for *E. roggenkampii* CapS, we found that the spacing of the wide palindrome perfectly matches the spacing of wHTH domains in a CapS dimer generated by interface 1 (Fig. 3A*,* bottom). Similarly, the spacing of the narrow palindrome perfectly matches the spacing of a CapS dimer generated by interface 2 (Fig. 3B*,* bottom).

**Figure EV3:**
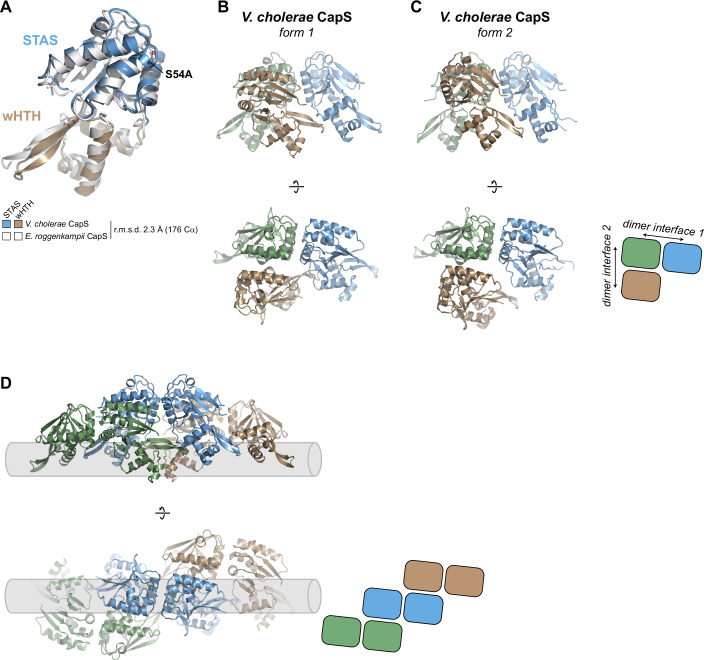
Structure of *V. cholerae* CapS (S58A). (**A**) Overlay of *V. cholerae* CapS (STAS domain blue; wHTH domain brown) from crystal form 1 with *E. roggenkampii* CapS (Cα r.m.s.d. 2.3 Å over 176 residues). (**B**) Two views of three non-crystallographic symmetry-related *V. cholerae* CapS protomers in crystal form 1, showing dimer interface 1 (green-blue) and dimer interface 2 (green-brown). (**C**) Two views of three non-crystallographic symmetry-related *V. cholerae* CapS protomers in crystal form 2, showing dimer interface 1 (green-blue) and dimer interface 2 (green-brown). (**D**) Two views of a *V. cholerae* CapS filament (six protomers) assembled from a combination of crystallographic and non-crystallographic symmetry. Three dimers assembled via interface 1 are colored green, blue, and brown, respectively. Each of these dimers associates with other dimers through interface 2. Shown in gray is a theoretical DNA duplex positioned such that all six CapS wHTH domains could bind it.

We next crystallized and determined two independent structures of a second CapS from *V. cholerae* 964015 (Fig. 1A) to a resolution of 2.38 Å (form 1; space group P3_1_21) and 1.84 Å (form 2; space group C222_1_), respectively (Appendix Table S1). *V. cholerae* CapS is 41% identical to *E. roggenkampii* CapS, and the two proteins overlay closely with an overall Cα r.m.s.d. of 2.3 Å (over 176 Cα pairs; Fig. EV3A). The two structures of *V. cholerae* CapS each contain five CapS protomers per asymmetric unit, and both structures show crystal packing interactions equivalent to both interface 1 and interface 2 in our structure of *E. roggenkampii* CapS (Fig. EV3B,C). Moreover, since dimer interfaces 1 and 2 do not overlap, a single CapS protomer can associate with two other CapS protomers via these two interfaces. In this manner, a continuous helical filament of CapS could self-assemble using these interfaces, with all protomers’ wHTH domains aligned to bind a single continuous DNA duplex (Fig. EV3D).

### CapK and CapS resemble bacterial anti-sigma factors and their antagonists

Structure comparisons with the DALI server show that the N-terminal STAS domain of CapS is most similar to bacterial anti-sigma factor antagonists (also known as anti-anti-sigma factors) from *Bacillus* (SpoIIAA) (Masuda et al, 2004) and Mycobacteria (RsfB; PDB ID 8IH8*unpublished*) (Fig. EV1D,E), which regulate important developmental switches in processes like sporulation and stress response (Dworkin and Losick, 2001; Moy and Seshu, 2021). These proteins contain a single STAS domain with a conserved serine residue that is phosphorylated by a GHKL-family histidine kinase domain in their cognate anti-sigma factors (*Bacillus* SpoIIAB and *Mycobacteria* RsbW, respectively). We found that CapS possesses an absolutely conserved serine in an equivalent position on the second α-helix of its STAS domain (*E. roggenkampii* CapS S54; Fig. 3C). To gauge whether phosphorylation of this residue might affect the oligomerization state of CapS in solution, we generated an *E. roggenkampii* CapS mutant with S54 mutated to glutamate (S54E), mimicking phosphorylation. By size exclusion chromatography coupled to multi-angle light scattering (SEC-MALS), we found that while wild-type CapS is dimeric, CapS S54E is monomeric (Fig. 3D). In our structure of *E. roggenkampii* CapS, S54 is buried in dimer interface 1, and surrounded by a highly conserved set of polar/negatively-charged residues (Fig. 3A,C), whereas this residue is not involved in dimer interface 2 (Fig. 3B). The fact that the S54E mutant disrupts the CapS dimer observed in solution therefore indicates that CapS likely dimerizes primarily through interface 1.

To assess how phosphorylation of CapS S54 affects DNA binding, we performed ChIP-Seq analysis. Strikingly, we found that the CapS S54E mutation resulted in a complete loss of binding to both sites in the *capK-capS* region (Fig. 3E). The CapS S54E mutation also resulted in constitutively high GFP expression in our GFP expression reporter (Fig. 3F). The presence of FLAG-tagged CapS in this construct further enabled us to measure CapS expression; we found that in the wild-type expression reporter, FLAG-CapS expression is low in the absence of DNA damage and high upon exposure to MMC (Fig. 3F). The CapS S54E mutation resulted in constitutively high expression of FLAG-CapS, mirroring the expression pattern of GFP. Overall, these data plus our in vitro DNA-binding assays support a model for CapS-DNA binding in which (1) “wide palindrome” sites can be directly bound by a CapS interface-1 dimer; (2) “narrow palindrome” sites recruit two CapS interface-1 dimers, that each bind one half-site of the narrow palindrome, and that associate with the neighboring CapS dimer through interface 2. This model explains the observed CapS-DNA binding cooperativity specific to the narrow palindrome (Fig. 2D). In both cases (narrow and wide), further oligomerization could occur through recruitment of additional CapS dimers to adjacent sites along the DNA. Such oligomerization explains the super-shift observed with longer 36-bp DNAs in our EMSA assays (Fig. 2F), and may also explain why the CapS-DNA-binding peaks we observe by ChIP-Seq are notably wider than would be expected from the post-shearing DNA fragment length of ~200 bp used in these experiments (Fig. 2A).

**Figure 4:**
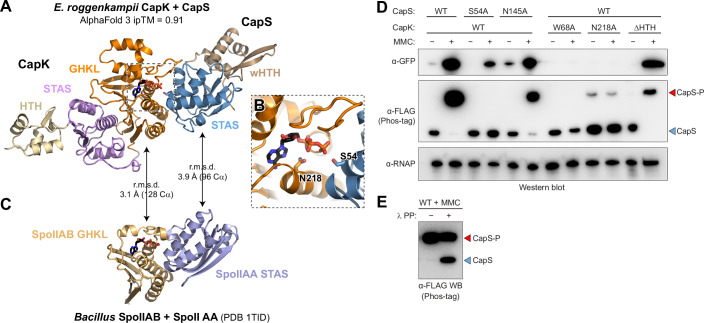
CapK phosphorylates CapS at a conserved serine residue. (**A**) AlphaFold 3-predicted structure of *E. roggenkampii* CapK (HTH yellow, STAS pink, GHKL orange) bound to CapS (STAS blue, wHTH brown) and ATP (black sticks). See Appendix Fig. S1B,C for AlphaFold confidence (pLDDT and PAE). (**B**) Closeup of the CapK GHKL kinase active site, showing the conserved CapK active site residue Asn145 and CapS Ser54 as sticks. (**C**) Crystal structure of the *Bacillus* SpoIIAB-SpoIIAA complex (PDB ID 1TID) (Masuda et al, 2004), aligned to CapK-CapS, with Cα r.m.s.d. values of the SpoIIAB and CapK GHKL domains, and the SpoIIAA and CapS STAS domains shown. ATP bound to the SpoIIAB GHKL domain is shown in sticks. (**D**) Western blots showing GFP expression (α-GFP, top) and CapS mobility shift (α-FLAG Phos-tag, middle) upon addition of mitomycin C (MMC) in the GFP expression reporter expressing FLAG-CapS. Unshifted FLAG-CapS is marked “CapS” and shifted FLAG-CapS is marked “CapS-P” for phosphorylation. CapK ΔHTH is a truncated construct missing the N-terminal HTH domain. α-RNAP: anti-RpoS RNA polymerase western blot (loading control). Western blots in panels (D)-(E) are representative of at least three independent trials. (**E**) Anti-FLAG western blot of CapS isolated from cells with the wild-type GFP expression reporter exposed to mitomycin C (MMC), then treated with lambda protein phosphatase (λ PP) before loading on the Phos-tag gel. Source data are available online for this figure.

Next, we sought to understand how CapK might bind and phosphorylate CapS. While we could purify a CapK-CapS complex using proteins from *V. cholerae* 964015 (Appendix Fig. S1A), this complex was not stable enough for stoichiometry analysis by SEC-MALS, or for experimental structure determination. Therefore, we used AlphaFold 3 to predict the structure of *E. roggenkampii* CapK bound to ATP and CapS (Fig. 4A,B; Appendix Fig. S1B,C). The two proteins are strongly predicted to bind one another (AlphaFold 3 ipTM = 0.91; values above 0.8 represent highly-confident interaction predictions (preprint: Kim et al, 2024)). In the resulting model, CapS S54 is positioned adjacent to the CapK histidine kinase active site, poised for phosphorylation (Fig. 4B). The predicted CapK-binding surface on CapS almost completely occludes CapS dimer interface 1, and partially occludes dimer interface 2 (Appendix Fig. S1D,E), suggesting that CapS binds CapK as a monomer, rather than as a dimer. This 1:1 stoichiometry is consistent with the relative band intensities we observe in SDS-PAGE analysis of the purified *V. cholerae* CapK-CapS complex (Appendix Fig. S1D).

The predicted structure of the CapK-CapS complex closely matches prior structures of *Bacillus* and Mycobacterial anti-sigma factor antagonists bound to their cognate kinase anti-sigma factors (Fig. 4C; Appendix Fig. S1F) (Masuda et al, 2004), suggesting that CapK might phosphorylate CapS. To test this idea, we used Phos-tag SDS-PAGE gels and detected a mobility shift of FLAG-CapS in our expression reporter upon addition of MMC, correlated with expression of GFP (Fig. 4D). Supporting the idea that this mobility shift is due to CapS phosphorylation, treatment of samples with lambda protein phosphatase (λ PP) reversed the mobility shift (Fig. 4E). Mutation of CapS S54 to alanine (S54A) prevented CapS phosphorylation, and strongly reduced MMC-dependent GFP expression (Fig. 4D). Mutation of CapS Asn145 to alanine (N145 A), which partially compromises DNA binding in vitro (Fig. EV2) resulted in a slight increase in baseline GFP expression (Fig. 4D), consistent with this mutation causing only a partial loss of DNA binding. Separately, mutation of a highly conserved asparagine residue in CapK’s kinase active site (N218A; Fig. 4B) strongly reduced CapS phosphorylation, and prevented MMC-dependent GFP expression (Fig. 4D). Finally, we tested the effect of deleting CapK’s N-terminal helix-turn-helix domain (ΔHTH). We did not observe any effect on MMC-activated GFP expression or CapS phosphorylation in this mutant, leaving the role of the HTH domain unknown (Fig. 4D). Overall, these data show that CapK phosphorylates CapS at a conserved serine upon DNA damage, dissociating the CapS homodimer and disrupting CapS-DNA binding, to mediate increased expression of both CapS and of the downstream operon.

### CapK is activated by single-stranded DNA

**Figure 5:**
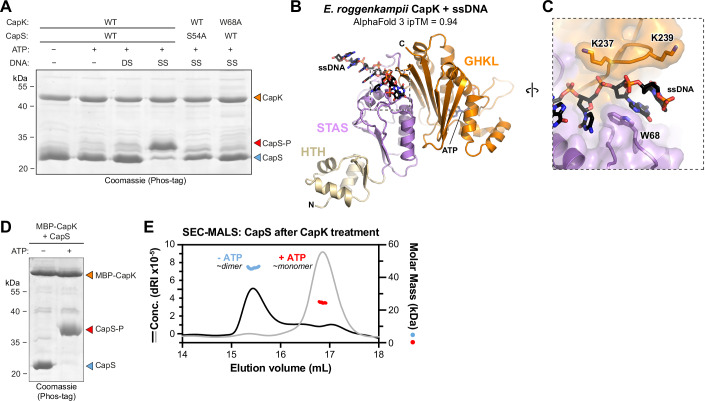
CapK is activated by ssDNA. (**A**) Phos-tag SDS-PAGE gel showing mobility shift of CapS upon treatment with CapK in the presence of ATP and single-stranded DNA. DS double-stranded DNA, SS single-stranded DNA. The data presented were representative of three independent trials. (**B**) AlphaFold 3-predicted structure of *E. roggenkampii* CapK (HTH domain yellow, STAS domain pink, GHKL domain orange) bound to ssDNA (black). See Appendix Fig. S2 for AlphaFold 3 confidence (pLDDT and PAE). (**C**) Closeup view of the predicted interaction between CapK and ssDNA, with CapK W68, K237, and K239 shown as sticks. (**D**) Phos-tag SDS-PAGE gel of CapS samples used for SEC-MALS analysis in panel (**E**). The data presented in panels (**D**, **E**) were representative of three independent trials. (**E**) SEC-MALS of CapS after treatment with MBP-tagged CapK and single-stranded DNA, in the absence of ATP (black/blue) or the presence of ATP (gray/red). Phosphorylated CapS shows a molecular weight consistent with a monomer (19.5 kDa), and unphosphorylated CapS shows a molecular weight consistent with a dimer (39 kDa). Source data are available online for this figure.

**Figure EV4:**
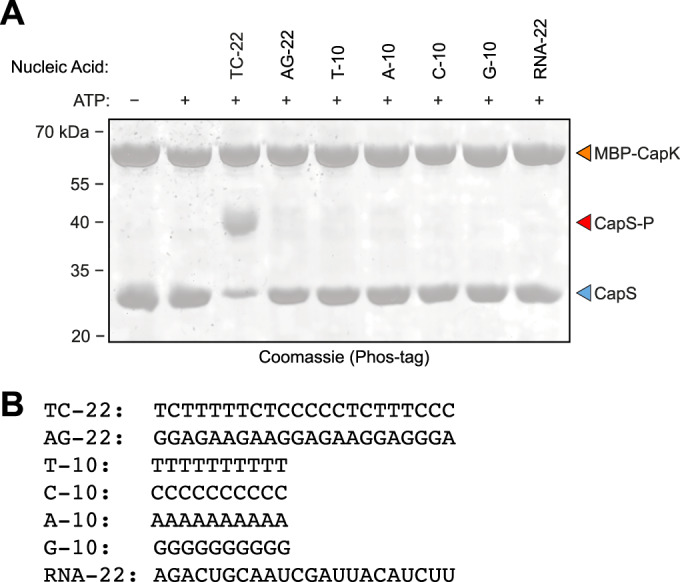
Single-stranded DNA activates CapK. (**A**) Phos-tag SDS-PAGE gel analysis of phosphorylation of CapS by MBP-tagged CapK, in the presence or absence of ATP and selected single-stranded nucleic acids. The data presented were representative of three independent trials. (**B**) Key showing the sequences of all nucleic acids used in panel (**A**). All are DNA except for “RNA-ss” which is RNA.Source data are available online for this figure.

Our data show that CapK+CapS de-represses transcription of the downstream operon when cells are treated with the DNA-damaging agent mitomycin C (MMC), and that de-repression requires UvrA and UvrB, proteins that act early in the NER DNA repair pathway. These data suggest that CapK’s kinase activity is activated by a byproduct of DNA repair. In prior work, we found that the transcriptional regulators CapW and CapP, which also respond to DNA damage, directly bind single-stranded DNA (ssDNA) for activation (Lau et al, 2022; Blankenchip and Corbett, 2024). To test whether CapK is similarly activated by ssDNA, we separately purified *E. roggenkampii* CapS and CapK and incubated the two proteins with ATP and either double-stranded or single-stranded DNA. We detected a mobility shift of CapS in Phos-tag gels that depended on CapK, ATP, and single-stranded DNA (Fig. 5A). This mobility shift was not observed when CapS S54 was mutated to alanine (S54A), confirming that the observed mobility shift is due to phosphorylation of S54 (Fig. 5A). We explored the sequence- and length-dependence of ssDNA activating CapK, and found that CapK requires a ~ 20 base ssDNA with a preference for T- and C-rich sequences, and that ssRNA does not activate the enzyme (Fig. EV4A,B).

We were unable to detect binding of CapK to ssDNA using a fluorescence polarization assay, so we instead performed an AlphaFold 3 structure prediction of *E. roggenkampii* CapK with a short poly-T ssDNA. The prediction shows a confident interaction (ipTM = 0.94) with ssDNA positioned in a positively-charged groove between the CapK STAS and GHKL kinase domains (Fig. 5B,C; Appendix Fig. S2). We identified a highly conserved tryptophan residue (W68) predicted to form pi-stacking interactions with the bound DNA (Fig. 5C). Mutation of W68 to alanine (W68A) eliminated ssDNA-stimulated kinase activity (Fig. 5A), supporting the AlphaFold model for CapK-ssDNA binding. The CapK W68A mutant also disrupted both CapS phosphorylation and GFP expression in our expression reporter (Fig. 4D).

To directly test whether phosphorylation disrupts the CapS dimer, we incubated wild-type *E. roggenkampii* CapS with MBP-tagged CapK, ATP, and ssDNA, then analyzed the reaction mixtures by both Phos-tag SDS-PAGE and SEC-MALS. We found that in the absence of ATP, CapS was not phosphorylated and was dimeric (Fig. 5D,E). In contrast, addition of ATP and ssDNA resulted in CapS phosphorylation and complete conversion to a monomeric state (Fig. 5D,E).

### Identification of a toxin-antitoxin locus with CapK and CapS-like proteins

**Figure 6:**
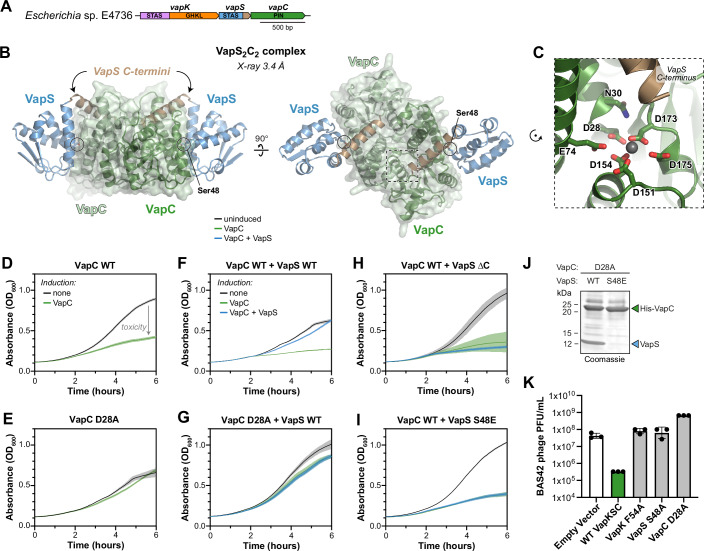
Identification of *vapKSC* operons. (**A**) Operon schematic of a *vapKSC* operon from *Escherichia* sp. E4736, with predicted domains of each protein noted. (**B**) Two views of the X-ray crystal structure of the *Escherichia* VapS_2_VapC_2_ complex, with VapS STAS domain colored blue and C-terminal region brown, and the VapC dimer colored dark green/light green. (**C**) Closeup of the VapC active site, with residues conserved across 279 VapC proteins in *vapKSC* operons shown as sticks, a bound Mg^2+^ ion shown in gray, and the nearby VapS C-terminus shown in brown. (**D**) Growth curves for *E. coli* cells transformed with an arabinose-inducible plasmid encoding *Escherichia* VapC, in non-inducing conditions (LB + 0.2% glucose, black line) or inducing conditions (LB + 0.2% arabinose, green line). Solid lines in panels (**D**–**I**) represent the average of three biological replicates, and shaded areas indicate standard deviation. (**E**) Growth curves for *E. coli* cells transformed with an arabinose-inducible plasmid encoding *Escherichia* VapC D28A. (**F**) Growth curves for *E. coli* cells transformed with an arabinose-inducible plasmid encoding *Escherichia* VapC plus an IPTG-inducible plasmid encoding VapS, in non-inducing conditions (LB + 0.2% glucose, black line), VapC-inducing conditions (LB + 0.2% arabinose, green line), and VapC + VapS-inducing conditions (LB + 0.2% arabinose + 50 µM IPTG, blue line). (**G**) Growth curves for *E. coli* cells transformed with an arabinose-inducible plasmid encoding *Escherichia* VapC D28A plus an IPTG-inducible plasmid encoding VapS. (**H**) Growth curves for *E. coli* cells transformed with an arabinose-inducible plasmid encoding *Escherichia* VapC plus an IPTG-inducible plasmid encoding VapS ΔC. (**I**) Growth curves for *E. coli* cells transformed with an arabinose-inducible plasmid encoding *Escherichia* VapC plus an IPTG-inducible plasmid encoding VapS S48E. (**J**) SDS-PAGE gel showing results of a Ni^2+^ pulldown assay after coexpression of His_6_-tagged VapC D28A with VapS wildtype (WT; left lane) or S48E (right lane). This experiment was performed once. (**K**) Plaque assay showing VapKSC-mediated protection of *E. coli* against phage AndreasVesalius (Bas42) (Maffei et al, 2021). Bars represent the average of three biological replicates, and error bars represent the standard deviation (individual data points also shown as black circles). Source data are available online for this figure.

In BLAST searches for CapK-related proteins in bacteria, we identified a family of proteins containing CapK-like STAS and GHKL kinase domains. Gene neighborhood analysis using webFLAGS (Saha et al, 2021) and *fast.genomics* (Price and Arkin, 2024) revealed that this protein is reproducibly found in a three-gene operon with an unknown protein containing a STAS domain and a short C-terminal region, and a VapC-like protein containing a predicted pilT N-terminal (PIN) nuclease domain (Fig. 6A). VapC is the toxin component of VapBC toxin-antitoxin (TA) systems, and cleaves RNAs when it is released from a complex with its antitoxin VapB (Pandey and Gerdes, 2005; Arcus et al, 2011). We hypothesized that this three-gene operon possesses a similar TA-like function, and named the operon *vapKSC*, encoding a CapK-like kinase (VapK), a STAS domain protein (VapS), and a VapC-like predicted nuclease. Through iterative BLAST searches in IMG, we identified 289 instances of *vapKSC* across diverse bacterial groups, and analysis of their gene neighborhoods using PADLOC (Payne et al, 2022) revealed that 103 (36%) are located within 10 kb of at least one other identified anti-phage immune gene (Dataset EV3).

Based on the function of VapBC systems and our findings with CapK and CapS, we hypothesized that VapS binds VapC and functions as an antitoxin, and that VapK phosphorylates VapS to disrupt the VapSC complex and activate VapC. Supporting this model, we were unable to express and purify a VapC protein from *Escherichia* sp. E4736 in *E. coli* due to toxicity, but we could co-express the protein with its cognate VapS. By SEC-MALS, we found that *Escherichia* VapS and VapC form a complex with 2:2 stoichiometry (Appendix Fig. S3). We crystallized and determined a 3.4 Å resolution X-ray crystal structure of the *Escherichia* VapSC complex (Appendix Table S1; Appendix Fig. S4), revealing a 2:2 heterotetramer with a central VapC dimer, and each VapC protomer bound to one protomer of VapS (Fig. 6B). VapS binds VapC primarily via its STAS domain, and the VapS C-terminus forms an α-helix that extends to within ~6 Å of the VapC catalytic site (Fig. 6B,C). Unlike VapB proteins, many of which use a conserved arginine residue to displace the catalytic Mg^2+^ ion from the VapC active site (Miallau et al, 2009; Dienemann et al, 2011; Min et al, 2012; Kang et al, 2020), VapS does not displace Mg^2+^ from the VapC active site (Fig. 6C). Instead, VapS may block binding of the proposed RNA target of VapC, preventing catalysis.

Sequence alignments of VapS reveal that, like CapS, this protein possesses an absolutely conserved serine or threonine residue (*Escherichia* VapS S48) in its STAS domain. In our structure of the VapSC complex, this residue is buried in the VapS-VapC interface (Fig. 6B). In an AlphaFold 3-predicted structure of a VapK-VapS complex, the conserved serine residue is positioned for phosphorylation by the VapK kinase domain, as in prior structures of bacterial anti-sigma factor antagonists and their cognate kinase anti-sigma factors (Appendix Fig. S5). To test whether VapS regulates VapC toxicity, we used a bacterial growth assay with VapC and VapS controlled by separate inducible promoters. As expected, expression of wild-type VapC but not a catalytic-dead VapC mutant (aspartate 28 to alanine; D28A) resulted in strong toxicity (Fig. 6D,E). Coexpression of VapS alongside wild-type VapC suppressed VapC-mediated toxicity, confirming that VapS is an antitoxin (Fig. 6F,G). Deletion of the VapS C-terminal region (residues 94–116) rendered the protein unable to counteract VapC toxicity, confirming that VapS directly inhibits VapC activity through its C-terminus (Fig. 6H). Mimicking VapS phosphorylation with a serine 48 to glutamate mutation (S48E) also renders VapS unable to counteract VapC toxicity (Fig. 6I). Supporting this idea, when we purified VapC (catalytic mutant D28A) from *E. coli* cells also expressing either wild-type VapS or the VapS S48E mutant, we found that VapC copurified with wild-type VapS but not the VapS S48E mutant (Fig. 6J).

To determine the activation mechanism of VapKSC, we performed an AlphaFold 3 prediction of a VapK-ssDNA complex, resulting in a high-confidence prediction with an ipTM of 0.94, showing ssDNA bound at the interface of the STAS and GHKL kinase domains (Appendix Fig. S6). Close inspection of the predicted structure identified a conserved phenylalanine residue in VapK (F54) equivalent to CapK W68 (Appendix Fig. S6B). These results suggest that, like CapK, VapK is activated by binding ssDNA resulting from DNA damage. We were unfortunately unable to test this idea: VapK is insoluble when expressed in *E. coli*; and we could not detect a FLAG-tagged VapS on western blots, presumably because of low expression. We did, however, find that the *vapKSC* operon provided a ~2-log protection against at least one bacteriophage, *E. coli* phage AndreasVesalius (Bas42) (Maffei et al, 2021), in the *Tequatovirus* family (Fig. 6K). Mutations of VapC (D28A), VapS (S48A), or the VapK putative ssDNA binding site (F54A) all eliminated protection against this phage (Fig. 6K). Overall, these data support a model in which DNA damage (arising from phage infection or other stress) activates VapK, which phosphorylates VapS, releasing it from VapC.

## Discussion

**Figure 7:**
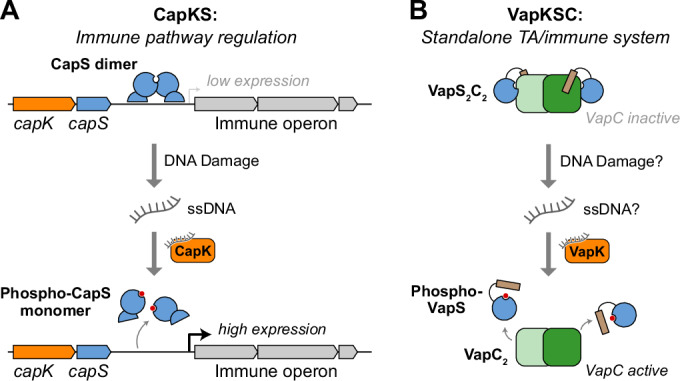
Proposed mechanism for CapKS and VapKSC. (**A**) Model for immune operon regulation by CapKS. In unperturbed cells, CapS forms a DNA-binding homodimer (blue) that suppresses transcription of its downstream operon (gray). Upon DNA damage, ssDNA is produced that binds and activates CapK (orange). CapK phosphorylates CapS (red circles), disrupting the CapS dimer and releasing it from DNA to allow increased expression of the downstream operon. (**B**) Model for VapKSC. In unperturbed cells, VapC (green) and VapS (blue/brown) form an inhibited VapC_2_S_2_ complex. Upon DNA damage and/or phage infection, ssDNA is produced that binds and activates VapK (orange). VapK phosphorylates VapS (red circles), releasing it from VapC to release inhibition of VapC.

Here we identify CapK and CapS, which control the expression of downstream immune operons—Bub, CBASS, and others—in response to DNA damage. Our data show that CapS is a homodimeric DNA-binding transcriptional repressor that cooperatively binds its own promoter and that of the downstream immune operon to suppress transcription (Fig. 7A). Upon binding ssDNA byproducts of DNA repair, CapK is activated and phosphorylates a conserved serine in the STAS domain of CapS, disrupting the CapS dimer to de-repress immune operon transcription (Fig. 7A). CapK+CapS is the newest example of immune operon-associated transcriptional regulators that sense and respond to DNA damage, a group that also includes CapW/BrxR (Blankenchip et al, 2022; Blankenchip and Corbett, 2024; Picton et al, 2022; Luyten et al, 2022) and CapH + CapP (Lau et al, 2022). These regulators may prevent toxicity arising from constitutive expression of immune operons, especially abortive infection systems like CBASS, whose high-level activation can kill the host cell (Ye et al, 2020; Cohen et al, 2019; Lopatina et al, 2020). Since phage infection often results in DNA damage and the production of excess ssDNA, sensing ssDNA allows these regulators to drive increased expression of immune pathways when they are needed most. Additionally, CapK + CapS and related regulators may enable a bacterium to coordinate immune responses between front-line restriction-modification pathways (which themselves generate DNA damage that could activate CapK) and later-acting abortive infection pathways (preprint: Oshiro et al, 2025). Supporting this idea, nearly 20% of identified *capK* +* capS* genes are encoded in a larger locus where *capK*+*capS* is sandwiched between an upstream restriction-modification operon and a downstream immune operon like CBASS or DUF4747/CarolAnn (Fig. 1A).

In addition to regulating expression of the downstream immune operon, our data shows that CapS regulates its own expression, binding its own promoter in unperturbed cells and dissociating when it is phosphorylated by CapK to boost its own expression. This autoregulatory circuit likely maintains CapS at levels that are (1) high enough to suppress both its own transcription and that of the downstream operon, but (2) low enough that activated CapK can readily phosphorylate the existing pool of CapS to release it from DNA. Strict control over CapS protein levels would therefore maintain the system in a poised state that is maximally sensitive to DNA damage arising from phage infection and/or activation of DNA-targeting immune pathways.

We find that CapS can bind both “wide” and “narrow” palindromic sequences with equivalent half-sites but significantly different spacing. The CapS homodimer that exists in solution is assembled through dimer interface 1, with the two CapS protomers’ wHTH domains perfectly positioned to bind a “wide” palindromic site. In contrast, “narrow” palindromic sites match the spacing between wHTH domains of CapS protomers assembled through interface 2. Our ChIP-Seq data suggests that CapS phosphorylation, which disrupts dimer interface 1, disrupts CapS binding to both wide and narrow palindromes. Together with our fluorescence polarization data showing that CapS binds cooperatively to the narrow palindrome, this data suggests that narrow palindrome binding involves two CapS interface-1 dimers binding neighboring sites along DNA and associating with one another through dimer interface 2. This difference in recognition mode between CapS binding sites in the downstream operon’s promoter (wide palindrome) and the *capS* promoter (narrow palindrome) means that the binding affinity for these sites could be independently tuned through mutations in the two CapS dimer interfaces, thereby enabling CapS to differentially regulate its own expression and that of the downstream operon. Testing this hypothesis will require further study, including isolation of a CapS mutant that specifically disrupts dimer interface 2.

Alongside CapK + CapS, we identify a related toxin/antitoxin system we term VapKSC, in which a CapS-like protein (VapS) binds and inhibits a VapC-family toxin (Fig. 7B). Our data suggest that the CapK-like kinase VapK is activated by ssDNA arising from DNA damage and/or phage infection. VapK-mediated phosphorylation of VapS causes dissociation of the VapC-VapS complex, resulting in activation of VapC and inhibition of cell growth, likely through cleavage of ribosomal RNA (rRNA) or tRNA. Our finding that a GHKL kinase + STAS domain protein pair functions in multiple contexts to regulate different biochemical activities in response to a common signal reflects the emerging theme of modularity in the evolution and function of bacterial immune pathways. As the number of identified immune pathways has expanded into the hundreds, diverse immune pathways have been shown to share common sensor domains, effector domains, and activation mechanisms (Mariano and Blower, 2023; Georjon and Bernheim, 2023). This modularity likely enables bacteria to swiftly respond to phages that evolve to evade specific immune pathways’ detection or effector mechanisms.

We identify *capK *+ *capS* adjacent to several known anti-phage immune pathways, including CBASS, Thoeris, Bil, and restriction-modification systems (Fig. 1B). The majority of *capK*+*capS* genes we identify are encoded adjacent to Bub operons, which can also be regulated by CapW or CapH+CapP, but have not been demonstrated to protect a host cell against phage infection (Ye et al, 2026). Bub operons, as well as other *capK *+ *capS*-associated genes, including *yegP*, may be anti-phage immune pathways or may instead mediate more global stress responses. Indeed, the line between anti-phage immune pathways and stress-response pathways is likely fuzzier than generally appreciated, with many pathways - like VapKSC and potentially other toxin-antitoxin systems - protecting their host against phage infection as a result of their response to a global stress signal like DNA damage.

## Methods

**Table:** Reagents and tools table

Reagent/resource	Reference or source	Identifier or catalog number
**Experimental models**		
*E. coli* Rosetta 2 (DE3) pLysS	Millipore Sigma	71401-M
*E. coli* Genlantis SoluBL21	Fisher Scientific	C700200
*E. coli* ΔRM	(Maffei et al, 2021)	*E. coli* K-12 MG1655 ΔRM
*E. coli* BW25113	(Baba et al, 2006)	Horizon Discovery Keio collection #OEC5042
*E. coli* BW25113 *ΔrecA*	(Baba et al, 2006)	Horizon Discovery Keio collection #JW2669
*E. coli* BW25113 *ΔrecB*	(Baba et al, 2006)	Horizon Discovery Keio collection #JW2788
*E. coli* BW25113 *ΔuvrA*	(Baba et al, 2006)	Horizon Discovery Keio collection #JW4019
*E. coli* BW25113 *ΔuvrB*	(Baba et al, 2006)	Horizon Discovery Keio collection #JW0762
*E. coli* BW25113 *ΔuvrC*	(Baba et al, 2006)	Horizon Discovery Keio collection #JW1898
*E. coli* BW25113 *ΔuvrD*	(Baba et al, 2006)	Horizon Discovery Keio collection #JW3786
**Recombinant DNA**		
pTRC99a	(Amann & Brosius, 1985)	NCBI #M22744.1
pET His6 TEV LIC cloning vector (2B-T)	Addgene	#29666
pET co-transformation cloning vector (13S-A)	Addgene	#48323
pBAD LIC cloning vector (8A)	Addgene	#37501
**Antibodies**		
Mouse anti-GFP primary antibody	Roche	11814460001
Mouse anti-FLAG primary antibody	Sigma-Aldrich	F3165
Mouse anti-RNA polymerase primary antibody	BioLegend	#10019-878
Goat anti-mouse HRP-linked secondary antibody	Millipore Sigma	12-349
**Oligonucleotides and other sequence-based reagents**		
6-FAM/TCATCTCCTTAAATTAACCATAAG	IDT	24 bp Narrow palindrome
CTTATGGTTAATTTAAGGAGATGA	IDT	24 bp Narrow palindrome complement
6-FAM/TCATCTCCCCGGGTTAACCATAAG	IDT	24 bp Narrow palindrome half-site mutant
CTTATGGTTAACCCGGGGAGATGA	IDT	24 bp Narrow palindrome half-site mutant complement
6-FAM/TGGTTAAACAGCTTTATTTAATGA	IDT	24 bp Wide palindrome
TCATTAAATAAAGCTGTTTAACCA	IDT	24 bp Wide palindrome complement
6-FAM/TGGCCGGGCAGCTTTATTTAATGA	IDT	24 bp Wide palindrome half-site mutant
TCATTAAATAAAGCTGCCCGGCCA	IDT	24 bp Wide palindrome half-site mutant complement
6-FAM/TAGTATCATCTCCTTAAATTAACCATAAGAAGCAGG	IDT	36 bp Narrow palindrome
CCTGCTTCTTATGGTTAATTTAAGGAGATGATACTA	IDT	36 bp Narrow palindrome complement
6-FAM/ACACCTGGTTAAACAGCTTTATTTAATGAGATTCTG	IDT	36 bp Wide palindrome
CAGAATCTCATTAAATAAAGCTGTTTAACCAGGTGT	IDT	36 bp Wide palindrome complement
**Chemicals, enzymes and other reagents**		
Phos binding reagent	APExBIO	F4002
Lambda Protein Phosphatase	New England Biolabs	P0753S
**Software**		
Graphpad Prism	GraphPad Software	version: 10.1.1
XDS	(Kabsch, 2010)	version: Jan 19, 2025
AIMLESS	(Evans and Murshudov, 2013)	version: 0.8.2
CTruncate	(Evans and Murshudov, 2013)	version: 1.13.0
PHASER	(McCoy et al, 2007)	version: 2.0
COOT	(Emsley et al, 2010)	version: 0.9.8
phenix.refine	(Adams et al, 2010)	version: 2.0

### Methods and protocols

#### Bioinformatics

Initial identification and annotation of *capK* and *capS* genes associated with Bub operons (Ye et al, 2026) was performed by manual inspection of gene neighborhoods using the Integrated Microbial Genomes & Microbiomes server (IMG: https://img.jgi.doe.gov) (Dataset EV1). For comprehensive identification of *capK* +* capS-* associated immune operons, representative CapK proteins were used as queries for exhaustive BLAST searches in IMG, followed by manual annotation of CapS-encoding genes (Dataset EV2). Genomic DNA sequences ± 10 kb of each *capKS* locus was downloaded from IMG and submitted to PADLOC (Payne et al, 2022) for identification of nearby immune operons. Gene neighborhoods were further annotated by manual inspection in IMG.

For identification of *vapKSC* operons, representative CapK homologs lacking the N-terminal HTH domain were used for exhaustive BLAST searches in IMG. Genomic DNA sequences ±10 kb of each *vapK* gene was downloaded from IMG and searched for *vapC* genes using tblastn in BLAST+ (installed locally). The same sequences were submitted to PADLOC for identification of nearby immune operons. Hits were manually annotated in IMG to identify all cases containing *vapK*, *vapS*, and *vapC* (Dataset EV3).

To generate sequence logos of the narrow and wide promoters in *capKS* loci (Fig. 2C,D), DNA sequences of 100 related *capS* genes (±200 bp) were aligned in Jalview (Troshin et al, 2011), and sequence logos were generated with WebLogo v. 3.9.0 (Crooks et al, 2004). FASTA files used to generate each logo (with IMG accession number for each associated *capS* gene) are supplied as source data.

### Cloning, expression, and protein purification

Sequences of all proteins used in this study are in Appendix Table S2. To generate the GFP reporter plasmid, the native sequence of a CapK + CapS operon and the associated system’s promoter from *Enterobacter roggenkampii* strain EB00176-1 (reverse complement of bases 880,520-882,344 of NCBI RefSeq NZ_CM130038.1) followed by a gene encoding GFP, was synthesized (IDT) and cloned via isothermal assembly into pTRC99a (Amann and Brosius, 1985), which encodes an IPTG-inducible promoter. Subsequently, an N-terminal FLAG epitope tag was introduced on *capS* using PCR mutagenesis. For ChIP-Seq, a longer insert including the entire intergenic region upstream of *capK* was cloned into pTRC99a (reverse complement of bases 880,520-883,004 of NCBI RefSeq NZ_CM130038.1) followed by a gene encoding GFP.

For protein expression, *E. roggenkampii* CapK (NCBI WP_001567866.1) and CapS (NCBI WP_001567865.1) were amplified by PCR and cloned into UC Berkeley Macrolab vectors 2B-T (Addgene #29666; encoding an N-terminal TEV protease-cleavable His_6_-tag), or 13S-A (Addgene #48323) for expression and purification. Point mutations were generated by PCR-based mutagenesis. Genes encoding full-length CapK (NCBI WP_160230705.1) and CapS (NCBI WP_046127252.1) from *V. cholerae* strain 964015 (genome sequence NCBI RefSeq NZ_JAWJBE020000004.1) were codon-optimized for *E. coli* expression, synthesized (IDT) and cloned into UC Berkeley Macrolab vector 2B-T or 13S-A.

To generate the VapKSC plasmid used for bacteriophage infection assays, the native sequence of a VapK+VapS+VapC operon from *Escherichia* sp. E4736 (bases 78125-80342 of NCBI RefSeq NZ_VATT01000011.1) was cloned into vector pTRC99a (Amann et al, 1988). *Escherichia* sp. E4736 VapK (NCBI WP_135559716.1), VapS (NCBI WP_135559717.1), and VapC (NCBI WP_135559718.1) were cloned into UC Berkeley Macrolab vectors 2B-T or 13S-A for expression and purification. For toxicity assays, VapC was cloned into pBAD LIC cloning vector (8A) (Addgene #37501; no tag), and VapS was cloned into UC Berkeley Macrolab vector 13S-A. Point mutations were generated by PCR-based mutagenesis.

### GFP expression reporter assays

For GFP reporter expression assays, *E. coli* MG1655 cells were transformed with GFP expression reporter plasmids (wildtype or indicated mutants), and 2 mL cultures in LB media plus 100 µg/mL ampicillin were grown overnight at 37 °C with shaking. The next morning, 50 µL of saturated overnight culture was diluted into 5 mL fresh media and grown to an OD_600_ of 0.3. Cells were stressed by the addition of 10 µg/mL mitomycin C (MMC), 10 µg/mL levofloxacin, 10 µg/mL colistin, or 100 µg/mL zeocin and grown for a further 1 h at 37 °C. For GFP expression analysis, 500 µL was centrifuged, then the cell pellet was resuspended in 50 µL 2X SDS-PAGE loading buffer (125 mM Tris-HCl pH 6.8, 20% Glycerol, 4% SDS, 200 mM DTT, 0.01% w/v bromophenol blue). Sample volumes were adjusted based on the culture density. Samples were placed in a boiling water bath for 3 min, then 5 µL samples were loaded onto a 4–20% Mini-PROTEAN TGX gel (Bio-Rad, 4561096) or 10% SDS-PAGE gel supplemented with 50 µM Phos binding reagent (APExBIO F4002) and separated at 180 V for 40 min at room temperature or 4 °C, respectively.

For GFP expression reporter assays in single-gene knockout strains, single-gene knockout *E. coli* strains from the KEIO collection (Baba et al, 2006) were obtained from Horizon Discovery (Strain IDs: parent strain BW25113 (Datsenko and Wanner, 2000); *ΔrecA* JW2669, *ΔrecB* JW2788, *ΔuvrA* JW4019, *ΔuvrB* JW0762, *ΔuvrC* JW1898, *ΔuvrD* JW3786).

### Western blots

For western blots, samples were separated on 4–20% Mini-PROTEAN TGX gels (Bio-Rad, 4561096) and then transferred to PVDF membranes (Bio-Rad Turbo transfer kit, 170-4272). PVDF membranes (Bio-Rad) were blocked for 1 h at room temperature with 5% nonfat dry milk in TBST (50 mM Tris-HCl pH 8.5, 150 mM NaCl, 0.1% Tween-20) followed by blotting for primary antibody overnight at 4 °C or room temperature for 1 h (Antibodies used: Mouse anti-GFP primary antibody (Roche, 11814460001) at 1:3000 dilution; Mouse anti-FLAG primary antibody (Sigma-Aldrich, F3165) at 1:5000 dilution; Mouse anti-RNA polymerase primary antibody (clone NT63; BioLegend #10019-878) at 1:3000 dilution). Blots were washed three times in TBST and incubated with Goat anti-mouse HRP-linked secondary antibody (Millipore Sigma, 12-349) at a dilution of 1:4000. Membranes were washed three times in TBST, incubated with ECL detection reagent (Cytiva RPN2232) for 1 min, and imaged on a Bio-Rad ChemiDoc imager.

### Protein expression and purification

Proteins were expressed in *E. coli* strain Rosetta 2 (DE3) pLysS (EMD Millipore). Cultures were grown in 1 L of 2XYT medium at 37 °C to A_600_ = 0.6, induced with 0.25 mM IPTG and moved to 20 °C for 16-18 h. Cells were harvested by centrifugation and resuspended in buffer A (25 mM Tris, pH 8.5, 10% glycerol, 300 mM NaCl, 5 mM MgCl_2_, and 1 mM NaN_3_) plus 5 mM imidazole and 5 mM β-mercaptoethanol and lysed by sonication (Branson sonifier). Lysates were clarified by centrifugation, then passed over Ni-NTA agarose (Qiagen) in resuspension buffer, washed in wash buffer (buffer A containing 20 mM imidazole), and eluted in elution buffer (buffer A containing 400 mM imidazole). Proteins were then concentrated by ultrafiltration (Amicon Ultra; EMD Millipore). All proteins were passed over a Superdex 200 Increase size exclusion column (Cytiva) in buffer A containing 1 mM DTT. Peak fractions were concentrated by ultrafiltration and stored at 4 °C.

### Crystallization and structure determination

For crystallization of *E. roggenkampii* CapS, purified protein at 9 mg/mL in crystallization buffer (25 mM Tris, pH 8.5, 0.2 M NaCl, 5 mM MgCl_2_, and 1 mM TCEP) was mixed 1:1 with well solution containing 0.2 M ammonium sulfate and 13% (w/v) PEG 4000 in hanging drop format. Crystals were collected into a cryoprotectant solution containing an additional 15% glycerol and frozen in liquid nitrogen. Diffraction data were collected at beamline 12-1 at the Stanford Synchrotron Light Source (SSRL), and processed with the autoPROC pipeline, which uses XDS (Kabsch, 2010) for indexing and integration, AIMLESS (Evans and Murshudov, 2013)for merging, and CTruncate (Evans and Murshudov, 2013) for conversion to structure factors. The structure was determined by molecular replacement in PHASER (McCoy et al, 2007) using an AlphaFold 3-predicted structure, manually rebuilt in COOT (Emsley et al, 2010), and refined in phenix.refine (Adams et al, 2010) using individual atomic position and isotropic B-factor refinement, plus TLS refinement (one TLS group per chain). Data collection and refinement statistics for all structures are in Appendix Table S1.

For crystallization of *V. cholerae* CapS (form 1), copurified CapK and CapS (S58A mutant) at 14 mg/mL plus 2 mM ATP in crystallization buffer (25 mM Tris pH 8.5, 0.2 M NaCl, 5 mM MgCl_2_, and 1 mM TCEP) was mixed 1:1 with well solution containing 0.2 M MgCl_2_, 0.1 M Bis-Tris pH 5.5, and 20% (w/v) PEG 3350 in hanging drop format. Crystals were collected into a cryoprotectant solution containing an additional 10% glycerol and frozen in liquid nitrogen. For crystallization of *V. cholerae* CapS (form 2), copurified CapK and CapS (S58A mutant) at 14 mg/mL plus 2 mM ATP in crystallization buffer (25 mM Tris pH 8.5, 0.2 M NaCl, 5 mM MgCl_2_, and 1 mM TCEP) was mixed 1:1 with well solution containing 0.2 M Ammonium sulfate, 0.1 M sodium acetate pH 4.6, and 17% (w/v) PEG 4000 in hanging drop format. Crystals were collected into a cryoprotectant solution containing an additional 10% glycerol and frozen in liquid nitrogen. For both form 1 and form 2, diffraction data were collected at SSRL beamline 9-2, processed with the autoPROC pipeline, and the structures were determined by molecular replacement in PHASER using AlphaFold 3-predicted structures. Models were manually rebuilt in COOT and refined in phenix.refine using individual atomic position and isotropic B-factor refinement, plus TLS refinement (one TLS group per chain).

For crystallization of the *Escherichia* VapS-VapC complex, copurified VapS + VapC at 15 mg/mL in crystallization buffer (25 mM Tris, pH 8.5, 0.2 M NaCl, 5 mM MgCl_2_, and 1 mM TCEP) was mixed 1:1 with a well solution containing 0.1 M HEPES, pH 7.5, 10% PEG 8000, and 0.2 M Calcium Acetate in sitting drop format. Crystals were collected into cryoprotectant solution containing an additional 20% glycerol and frozen in liquid nitrogen. Diffraction data were collected at the Advanced Photon Source at Argonne National Laboratory, on beamline 24ID-C. Data were indexed and integrated with XDS (Kabsch, 2010) in the RAPD2 pipeline (https://git.nec.aps.anl.gov/rapd/rapd). Because of high anisotropy, unmerged data were uploaded to the STARANISO web server (https://staraniso.globalphasing.org/) for application of an ellipsoidal resolution cutoffs (4.46 Å along a* and b*; 3.4 Å along c*) and anisotopy-corrected scaling. Values in Appendix Table S1 are after STARANISO processing and merging. Graphs showing merging statistics by resolution (completeness, multiplicity, I/σ, *R*_merge_/*R*_sym_/*R*_pim_, and CC_1/2_) are shown in Appendix Fig. S4. The structure was determined by molecular replacement in PHASER (McCoy et al, 2007) using an AlphaFold 3 model of a 2:2 VapS-VapC complex (2 heterotetramers placed). The N-terminal tag for two VapS protomers, plus Mg^2+^ ions in each VapC active site, were manually built in COOT (Emsley et al, 2010). The structure was refined to a resolution of 3.4 Å in phenix.refine (Adams et al, 2010) with reference model restraints (using the input AlphaFold 3 model of VapS-VapC), strict non-crystallographic symmetry constraints, and grouped B-factors (two groups per residue).

### SEC-MALS

For characterization of CapS and VapS-VapC oligomeric states by size exclusion chromatography coupled to multi-angle light scattering (SEC-MALS), 100 µL of purified protein at a concentration of 2 mg/mL was injected onto a size exclusion column (Superdex 200 Increase 10/300 GL, Cytiva) in SEC-MALS buffer (25 mM Tris pH 8.5, 300 mM NaCl, 5 mM MgCl_2_, 1 mM NaN_3_, 1 mM DTT, and 5% glycerol). Light scattering and differential refractive index (dRI) profiles were collected using miniDAWN TREOS and Optilab T-rEX detectors (Wyatt Technology). SEC-MALS data were analyzed using ASTRA software version 8 and visualized with Graphpad Prism v.10.1.1.

### DNA-binding assays

For characterization of DNA binding by fluorescence polarization assays, 24-base pair double-stranded DNAs were produced by annealing complementary oligos (sequences below), with the top strand 5’ 6-FAM labeled. FP reactions (30 µL) in buffer 25 mM Tris pH 8.5, 50 mM sodium glutamate, 5 mM MgCl_2_, 1 mM DTT, 5% glycerol, and 0.01% NP40 analog contained 50 nM DNA and the indicated protein concentration. FP was read using a Tecan Spark plate reader, and binding data were analyzed with Graphpad Prism v.10.1.1 using either a single-site binding model (Y = B_max_*X/(*K*_*d*_ + X) + Background) or a cooperative binding model (Y = B_max_*X^h^/(*K*_*d*_^h^ + X^h^)). Top-strand sequences for oligos: narrow palindrome (TCATCTCCTTAAATTAACCATAAG), narrow palindrome half-site mutant (TCATCTCCCCGGGTTAACCATAAG), wide palindrome (TGGTTAAACAGCTTTATTTAATGA), wide palindrome half-site mutant (TGGCCGGGCAGCTTTATTTAATGA).

For electromobility shift DNA-binding assays (EMSA), 36-base pair double-stranded DNAs were produced by annealing complementary oligos (sequences below), with the top strand 5’ 6-FAM labeled. About 12 µL reactions were assembled in binding buffer (25 mM Tris, pH 8.5, 50 mM NaCl, 1 mM DTT, 5 mM MgCl_2_, and 5% glycerol) with 50 nM DNA and the indicated concentration of protein, and incubated for 30 min at room temperature. Reactions were loaded onto 10% TBE-PAGE gels in 1x TBE pH 8.5 running buffer, run for 1.5 h at 4 °C, and imaged on a Bio-Rad ChemiDoc imager. Top-strand sequences for oligos: wide (ACACCTGGTTAAACAGCTTTATTTAATGAGATTCTG), narrow (TAGTATCATCTCCTTAAATTAACCATAAGAAGCAGG).

### CapK kinase assays

For detecting phosphorylation of CapS by CapK in cells, DNA damage assays were performed with 10 µg/mL mitomycin C and the *E. roggenkampii* GFP reporter system with a FLAG-tag fused to the N-terminus of CapS. The resuspended cell pellet in 2x SDS-PAGE loading buffer was boiled for 3 min, and 5 µL of the sample was loaded onto a 10% SDS-PAGE gel containing 50 uM of Phos binding reagent (APExBIO F4002). Gels were run at 180 V for 40 min at 4 °C and washed twice with 5 mM EDTA for 10 min before western blotting.

For detection of CapK kinase activity in vitro, 20 µL reactions containing 10 µM purified *E. roggenkampii* CapK and CapS, 2 mM ATP, and 10 uM ssDNA in reaction buffer (20 mM HEPES, pH 7.5, 100 mM NaCl, 20 mM MgCl_2_, 1 mM DTT) were incubated 1 h at 37 °C, then added to 20 µL 2x SDS sample buffer. About 10 µL of each sample was loaded onto a 10% SDS-PAGE gel containing 50 uM of Phos binding reagent (APExBIO F4002) and Coomassie-stained for visualization.

### Chromatin immunoprecipitation (ChIP) and sequencing

For chromatin immunoprecipitation experiments, *E. coli* MG1655 cells transformed with the *capK-capS* region plasmid (wild-type or *capS*-S54E) were grown from fresh streaks in LB medium with ampicillin, inoculating 10 mL secondary cultures with 100 μL overnight culture and growing to an OD₆₀₀ of ~0.3. Where indicated, DNA damage was induced by the addition of mitomycin C to a final concentration of 5 μg/mL, followed by incubation for 1 h; untreated controls were processed in parallel. Cultures were crosslinked by the addition of molecular-grade formaldehyde to a final concentration of ~1% and incubated for 10 min at 37 °C with shaking, followed by quenching with glycine (final concentration 125 mM) for 10 min. Cells were pelleted, washed twice with ice-cold PBS, and stored at −80 °C until processing.

Frozen pellets were thawed on ice and lysed in cell lysis buffer (50 mM HEPES pH 8.0, 1 mM EDTA, 85 mM KCl, and 0.5% NP40) supplemented with protease inhibitors. Chromatin was transferred to Covaris microTUBEs and sheared to an average fragment size of ~200 bp. After sonication, samples were clarified by centrifugation to remove debris, and an aliquot of input chromatin was de-crosslinked, proteinase K-treated, and purified to assess fragment size using an Agilent TapeStation (D1000). For immunoprecipitation, chromatin was diluted in dilution buffer (0.1% SDS, 1.1% Triton X-100, 1.2 mM EDTA, 165 mM NaCl, 16.7 mM Tris-HCl, pH 8.1) and incubated with antibody-conjugated magnetic beads for 2 h at 4 °C with rotation; mock IPs were performed using non-specific beads. Beads were sequentially washed with low-salt buffer (Tris-HCl 50 mM, pH 8.0, 150 mM NaCl, 0.1% SDS, 1% NP40, 1 mM EDTA, and 0.5% Deoxycholate Na), high-salt buffer (Tris•HCl 50 mM, pH 8.0, 500 mM NaCl, 0.1% SDS, 1% NP40, 1 mM EDTA, and 0.5% Deoxycholate Na), LiCl buffer (Tris-HCl 50 mM, pH 8.0, 250 mM LiCl, 0.1% SDS, 1% NP40, 1 mM EDTA, and 0.5% Deoxycholate Na), and TE buffer (Tris•HCl 10 mM, pH 8.0, and 0.25 mM EDTA), followed by elution in 1% SDS, 100 mM NaHCO₃ at 65 °C. Crosslinks were reversed by overnight incubation at 65 °C, samples were treated with proteinase K, and DNA was purified using Zymo columns (D4004).

ChIP and Mock DNA were converted into sequencing libraries using NEBNext Ultra II DNA Library Prep reagents with indexed adapters, followed by PCR amplification. Library quality and fragment size distributions were assessed on an Agilent TapeStation 4150. Libraries were pooled and sequenced on an Illumina NovaSeq platform using paired-end 150 bp reads, yielding >20 million reads per sample. Sequencing quality was evaluated using FastQC prior to downstream analysis.

Three independent biological replicates for each sample were sequenced. High-quality reads were selected for further analysis. The reads were aligned to the plasmid sequence using bowtie 2/2.5.0 (Langmead et al, 2009; Langmead and Salzberg, 2012). PCR duplicates were removed following best practices for paired-end data using samtools fixmate and markdup (Danecek et al, 2021; Li et al, 2009). Only primary, properly mapped read pairs were retained for downstream analyses. Genome-wide coverage tracks were generated from deduplicated BAM files using bedtools genomecov, normalized to reads per million mapped reads (RPM), and subsequently aggregated into fixed-width bins (10–20 bp) to reduce local noise on the small plasmid genome. Because the ChIP-seq signal is strand-independent, all coverage tracks were generated without strand filtering. Enrichment was calculated as the log2 ratio of IP over Mock RPM-normalized coverage, with the addition of a small pseudocount to avoid division by zero. For each replicate, putative binding regions were identified using a percentile-based approach, defining enriched bins as those within the top 0.5–1% of genome-wide log2 enrichment values, rather than applying a fixed fold-enrichment cutoff. Adjacent enriched bins were merged if separated by ≤40 bp, and only regions ≥40 bp in length were retained. To ensure robustness, enrichment profiles were assessed independently for each biological replicate, and only regions reproducibly detected in at least two replicates per condition were considered high-confidence binding sites. All coverage and enrichment tracks, as well as reproducible peak intervals, were visualized in the Integrative Genomics Viewer (IGV) using consistent genomic coordinates and fixed y-axis scales to enable direct comparison across conditions.

### Toxicity assays

For bacterial growth curves, overnight cultures of *E. coli* strain SoluBL21 (Genlantis) with plasmids of interest were grown at 37 °C in LB plus appropriate antibiotics. Cultures were diluted OD_600_ = 0.1 in fresh LB with antibiotics and grown at 37 °C until OD_600_ = 0.5. The cells were diluted back to OD_600_ = 0.1 with LB plus antibiotics and the inducers (0.2% arabinose and 50 μM IPTG). 100 μL of diluted cultures were plated to a standard clear 96-well plate with lid (Corning) and incubated in a plate reader (Tecan Spark) at 37 °C. The OD600 of each well was measured every 3 min for 8 h total with medium intensity shaking. Each sample included three independent replicates on a single plate.

For toxicity spotting assays, overnight cultures of *E. coli* strain MG1655 with plasmids of interest were grown at 37 °C in LB plus appropriate antibiotics. Cultures were diluted OD_600_ = 0.1 in fresh LB with antibiotics supplemented with 0.2% glucose to prevent toxicity and grown at 37 °C until OD_600_ = 0.5. Tenfold serial dilutions of these cultures were prepared in LB broth, and 3 μl of these dilutions was spotted onto LB agar plates containing the appropriate antibiotic and inducer (0.2% arabinose) or suppressor (0.2% glucose). The plates were incubated overnight at 37 °C and were imaged the next day using a ChemiDoc Imaging System (Bio-Rad).

### Bacteriophage infectivity

Plaque-Forming Unit (PFU) estimations were used as a measure of phage infections. Briefly, M9 minimal media agar plates were prepared with 10 μM IPTG and left to dry overnight at room temperature. Five overnight starter cultures of *E. coli* ΔRM (gift of Alexander Harms) (Maffei et al, 2021) harboring either the pTRC99a empty vector or *vapKSC* in pTRC99a (wildtype and indicated mutants) were diluted in M9 minimal media + ampicillin and grown at 37 °C until the OD_600_ reached 0.6-0.8. Next, 0.1 mL of this culture was mixed with 5 mL of preheated 0.35% top agar (composed of M9 minimal media plus 0.7% agar at a 1:1 ratio) at 55 °C, and the mixture was overlaid onto M9+ampicillin agar plates. Tenfold dilutions of the Bas42 phage stock was prepared in the BASEL phage buffer (0.05 M Tris, pH 7.5, 0.1 M NaCl, and 10 mM MgSO_4_) as the diluent. After 1 h of air drying of plates, 3 μL volume of tenfold phage dilutions were spotted on these plates and allowed to absorb into the top agar for 30 min, followed by overnight incubation at 37 °C. The plaques were quantified the next day by counting individual plaques in the highest dilution at which individual plaques were visible, and the reported values are the mean plus standard deviation of three biological replicates. Images were taken using an Epson flatbed scanner.

## Supplementary information

Appendix

Peer Review File

Dataset EV1

Dataset EV2

Dataset EV3

Structures

Source data Fig. 1

Source data Fig. 2

Source data Fig. 3

Source data Fig. 4

Source data Fig. 5

Source data Fig. 6

Source Data for Expanded View and Appendix Figures

Expanded View Figures

## Data Availability

The datasets produced in this study are available in the following databases: Model and maps for *E. roggenkampii* CapS: Protein Data Bank 9Z71 (10.2210/pdb9Z71/pdb). Model and maps for *V. cholerae* CapS S58A form 1: Protein Data Bank 9Z72 (10.2210/pdb9Z72/pdb). Model and maps for *V. cholerae* CapS S58A form 2: Protein Data Bank 9Z73 (10.2210/pdb9Z73/pdb). Model and maps for *Escherichia* VapS-VapC: Protein Data Bank 9Z7O (10.2210/pdb9Z7O/pdb). Diffraction data for *E. roggenkampii* CapS: SBGrid Data Bank 1238 (https://data.sbgrid.org/dataset/1238/). Diffraction data for *V. cholerae* CapS S58A form 1: SBGrid Data Bank 1239 (https://data.sbgrid.org/dataset/1239/). Diffraction data for *V. cholerae* CapS S58A form 2: SBGrid Data Bank 1240 (https://data.sbgrid.org/dataset/1240/). Diffraction data for *Escherichia* VapS-VapC: SBGrid Data Bank 1241 (https://data.sbgrid.org/dataset/1241/). ChIP-Seq Data: NCBI SRA BioProject PRJNA1415371 (http://www.ncbi.nlm.nih.gov/bioproject/1415371). The source data of this paper are collected in the following database record: biostudies:S-SCDT-10_1038-S44318-026-00831-y.
