# Supplementary material for: A DNA damage-activated kinase phosphorylates a transcriptional repressor to control bacterial immune pathway expression
Source: EMBO J. 2026 Jun 9;45(14):5079–100. doi: 10.1038/s44318-026-00831-y (PMC13373217; doi:10.1038/s44318-026-00831-y)
Supplement: Supplementary file 1 — Appendix [file 44318_2026_831_MOESM1_ESM.pdf]

Appendix for

A DNA damage-activated kinase phosphorylates a transcriptional repressor to control bacterial immune pathway expression

Table of Contents

**Appendix Figure S1**	CapK and CapS resemble bacterial anti-sigma factors and their antagonists	page 2
**Appendix Figure S2**	AlphaFold 3 predicted model of the *E. roggenkampii* CapK-ssDNA complex	page 3
**Appendix Figure S3**	SEC-MALS of *Escherichia* VapS-VapC	page 4
**Appendix Figure S4**	Anisotropic processing of *Escherichia* VapS-VapC crystallographic data	page 5
**Appendix Figure S5**	AlphaFold 3 predicted model of the *Escherichia* VapK-VapS complex	page 6
**Appendix Figure S6**	AlphaFold 3 predicted model of the *Escherichia* VapK-ssDNA complex	page 7
**Appendix Table S1**	Crystallographic data collection and refinement statistics	page 8
**Appendix Table S2**	Proteins used in this study	page 9

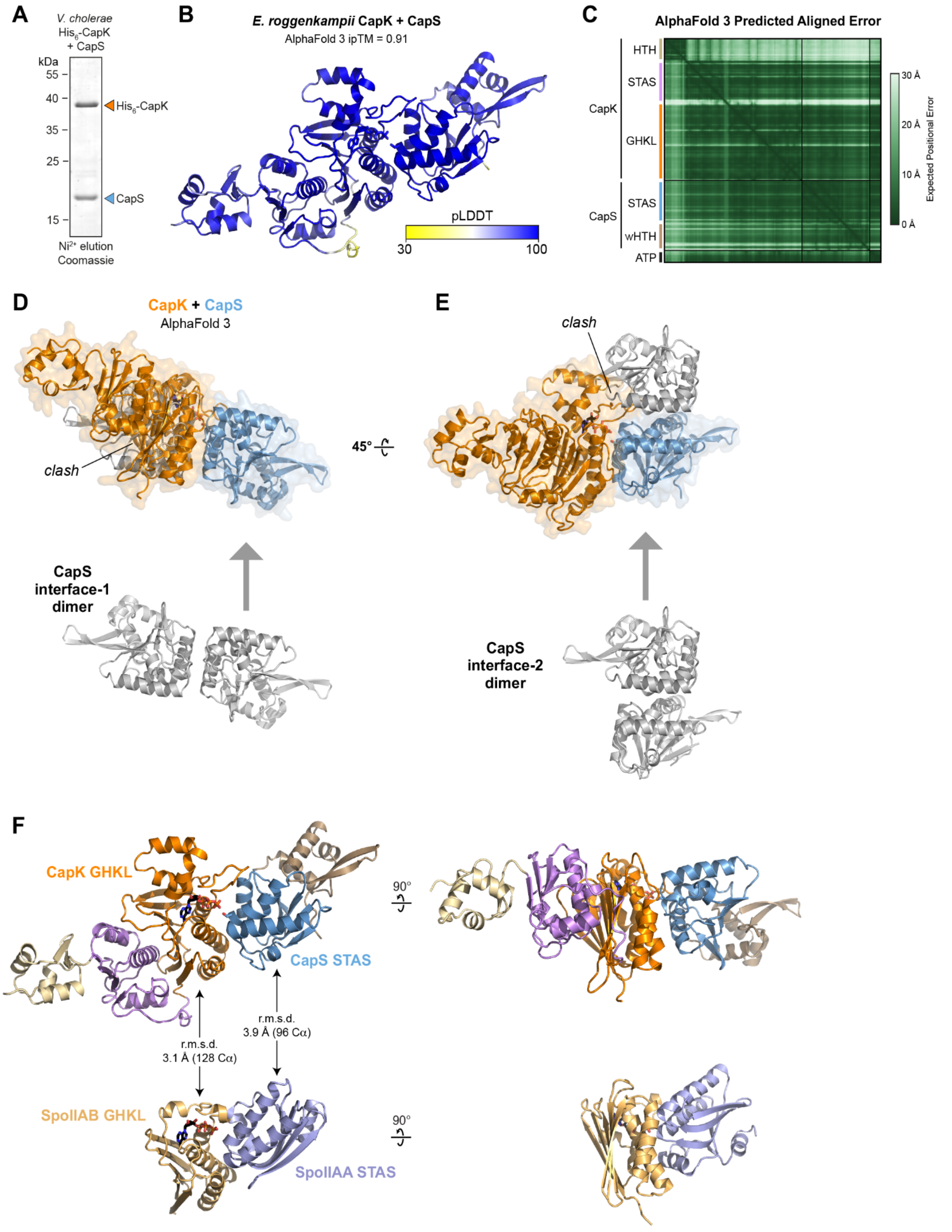

Appendix Figure S1 - CapK and CapS resemble bacterial anti-sigma factors and their antagonists.

(A) Ni^{2+} elution of co-expressed *V. cholerae* His_6-CapK and untagged CapS. Data presented in panels (C)-(D) is representative of three independent trials. **(B)** AlphaFold 3 predicted structure of *E. roggenkampii* CapK bound to CapS, colored by confidence (pLDDT). **(C)** Predicted Aligned Error (PAE) plot for the model shown in panel (A). **(D)** *Top:* Two views of the AlphaFold 3 predicted structure of *E. roggenkampii* CapK bound to CapS, colored by domain as in **Figure 4A**. ATP bound to the CapK GHKL domain is shown in sticks. *Bottom:* Two views of the *Bacillus* SpoIIAB-SpoIIAA complex (PDB ID 1TID) (Masuda *et al*, 2004), aligned to CapK-CapS. ATP bound to the SpoIIAB GHKL domain is shown in sticks.

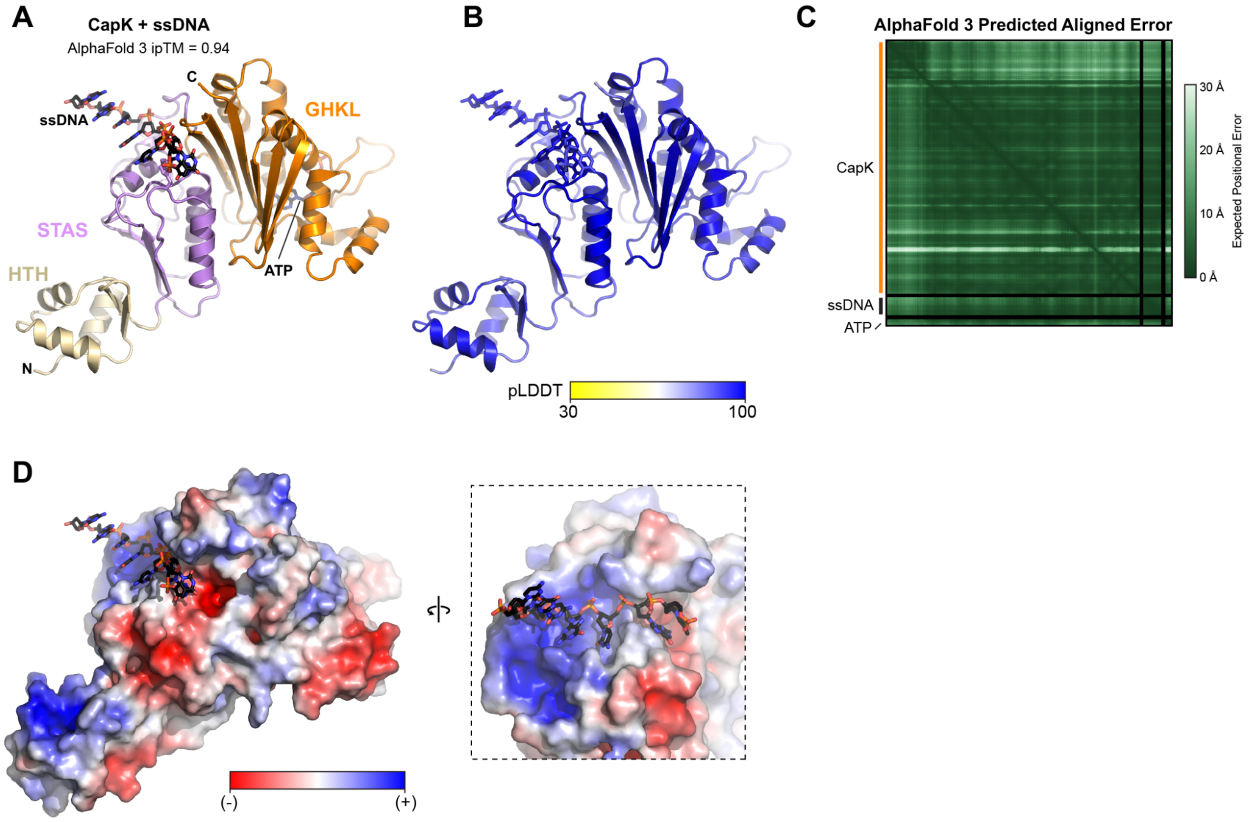

Appendix Figure S2 - AlphaFold 3 predicted model of the *E. roggenkampii* CapK-ssDNA complex.

(A) AlphaFold 3 predicted model of a complex of *E. roggenkampii* CapK (HTH domain yellow, STAS domain pink, GHKL domain orange), poly-T ssDNA (black), ATP (black), and Mg^{2+} (gray; not visible). CapK Trp68 is shown as sticks. **(B)** View as in panel (A), colored by confidence (pLDDT). **(C)** Predicted Aligned Error (PAE) plot for the model shown in panel (A). **(D)** Two views the predicted CapK-ssDNA structure, with CapK shown as a molecular surface and colored by charge (negative charge red, positive charge blue).

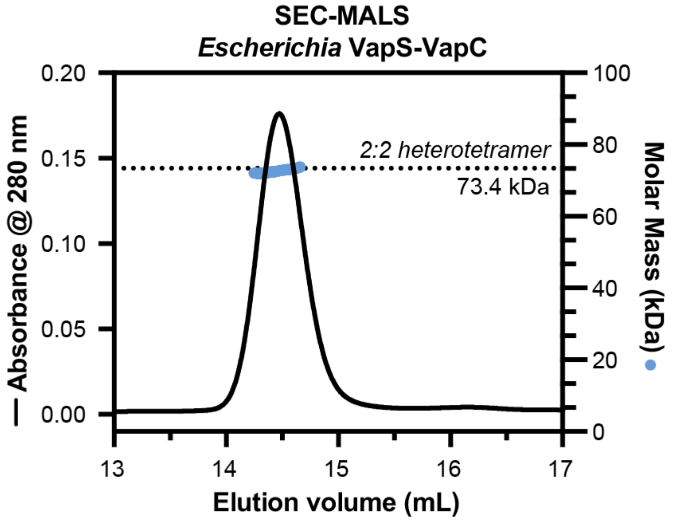

Appendix Figure S3 - SEC-MALS of *Escherichia* VapS-VapC.

Size exclusion chromatography coupled to multi-angle light scattering (SEC-MALS) of the Escherichia VapS-VapC complex. Black line indicates absorbance at 280 nm, and blue circles indicate measured molecular weight. A dotted line indicates the molecular weight of a 2:2 heterotetramer of VapS and VapC. This experiment was performed once.

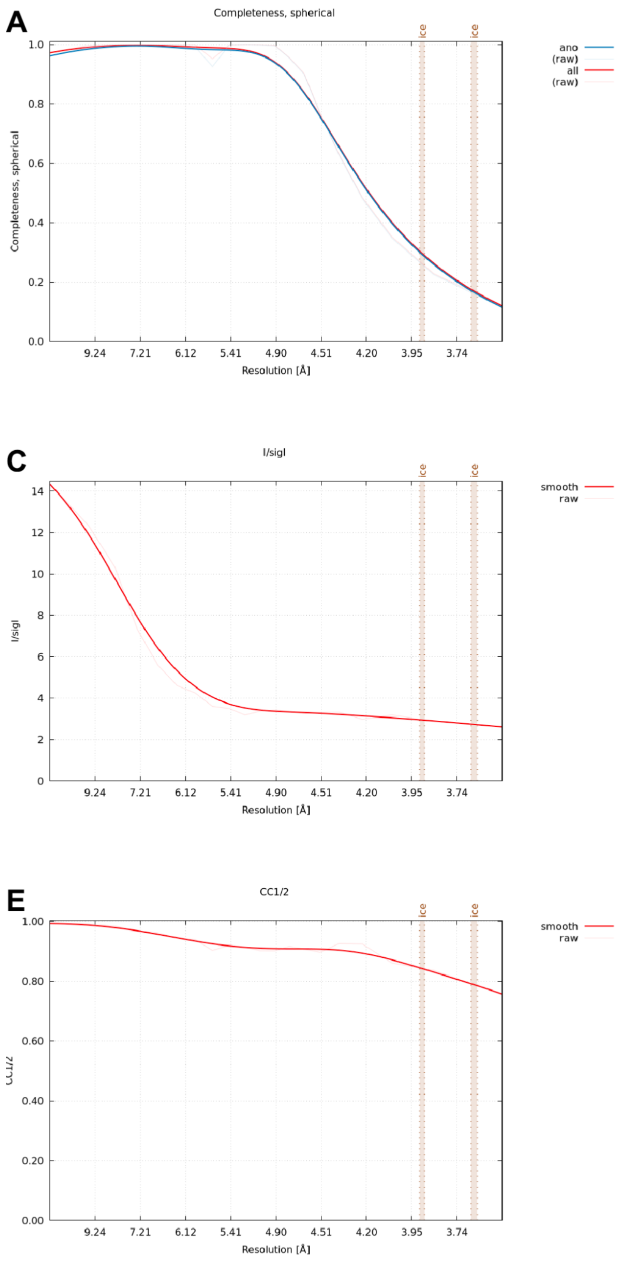

Appendix Figure S4 - Anisotropic processing of *Escherichia* VapS-VapC crystallographic data.

(A) Graph of completeness versus resolution for the STARANISO-processed X-ray diffraction dataset of *Escherichia* VapS-VapC. Red line indicates native completeness; blue line indicates anomalous completeness. Brown vertical bars indicate resolution ranges typical of ice crystal diffraction. **(B)** Graph of multiplicity versus resolution for the STARANISO-processed X-ray diffraction dataset of *Escherichia* VapS-VapC. **(C)** Graph of intensity (I/σI) versus resolution for the STARANISO-processed X-ray diffraction dataset of *Escherichia* VapS-VapC. **(D)** Graph of *R* values versus resolution for the STARANISO-processed X-ray diffraction dataset of *Escherichia* VapS-VapC. Red line indicates R_{merge}; blue line indicates R_{meas}, and green line indicates R_{pim}. **(E)** Graph of $CC_{1/2}$ versus resolution for the STARANISO-processed X-ray diffraction dataset of *Escherichia* VapS-VapC.

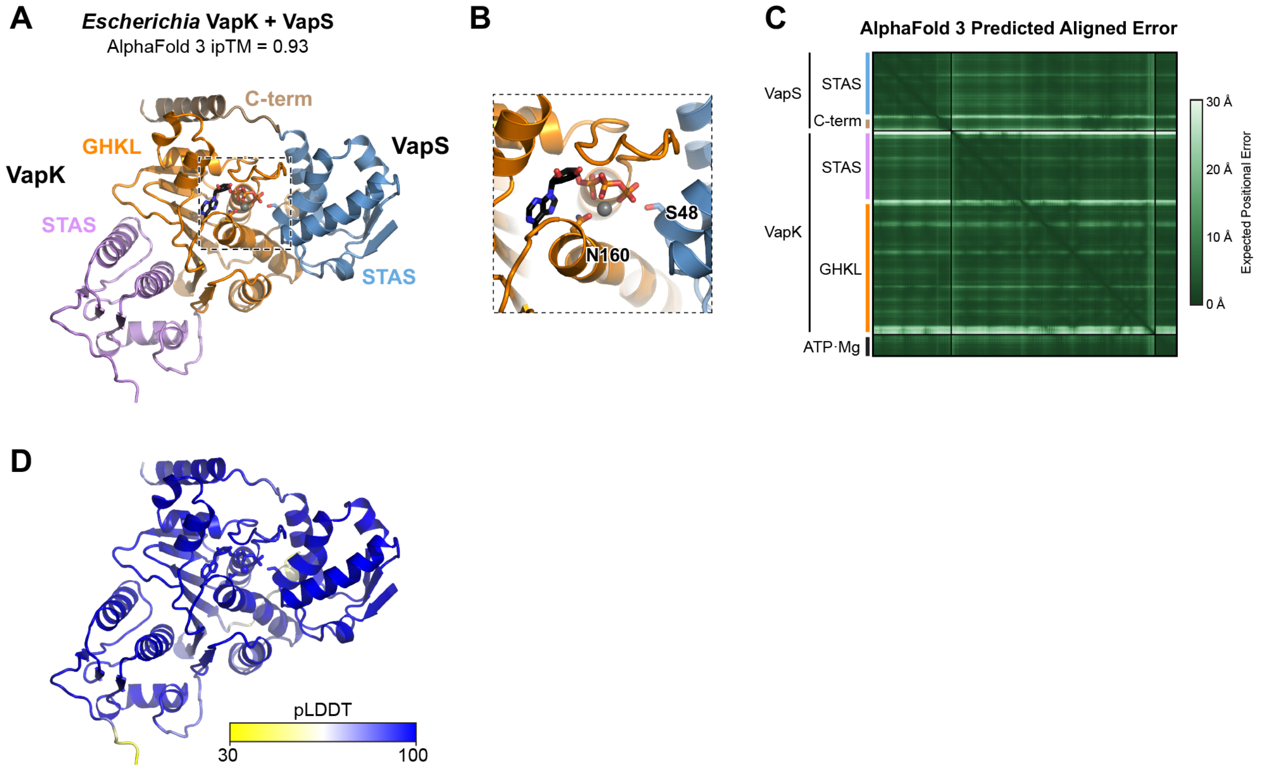

Appendix Figure S5 - AlphaFold 3 predicted model of the *Escherichia* VapK-VapS complex.

(A) AlphaFold 3 predicted model of a complex of *Escherichia* VapK (STAS domain pink, GHKL domain orange), VapS (STAS domain blue, C-terminal region brown), ATP (black), and Mg^{2+} (gray). **(B)** Closeup of the VapK GHKL kinase active site, with VapK Asn160 and VapS Ser48 shown as sticks. **(C)** AlphaFold 3 predicted aligned error (PAE) plot for the prediction shown in panel (A). **(D)** View as in panel (A), colored by confidence (pLDDT).

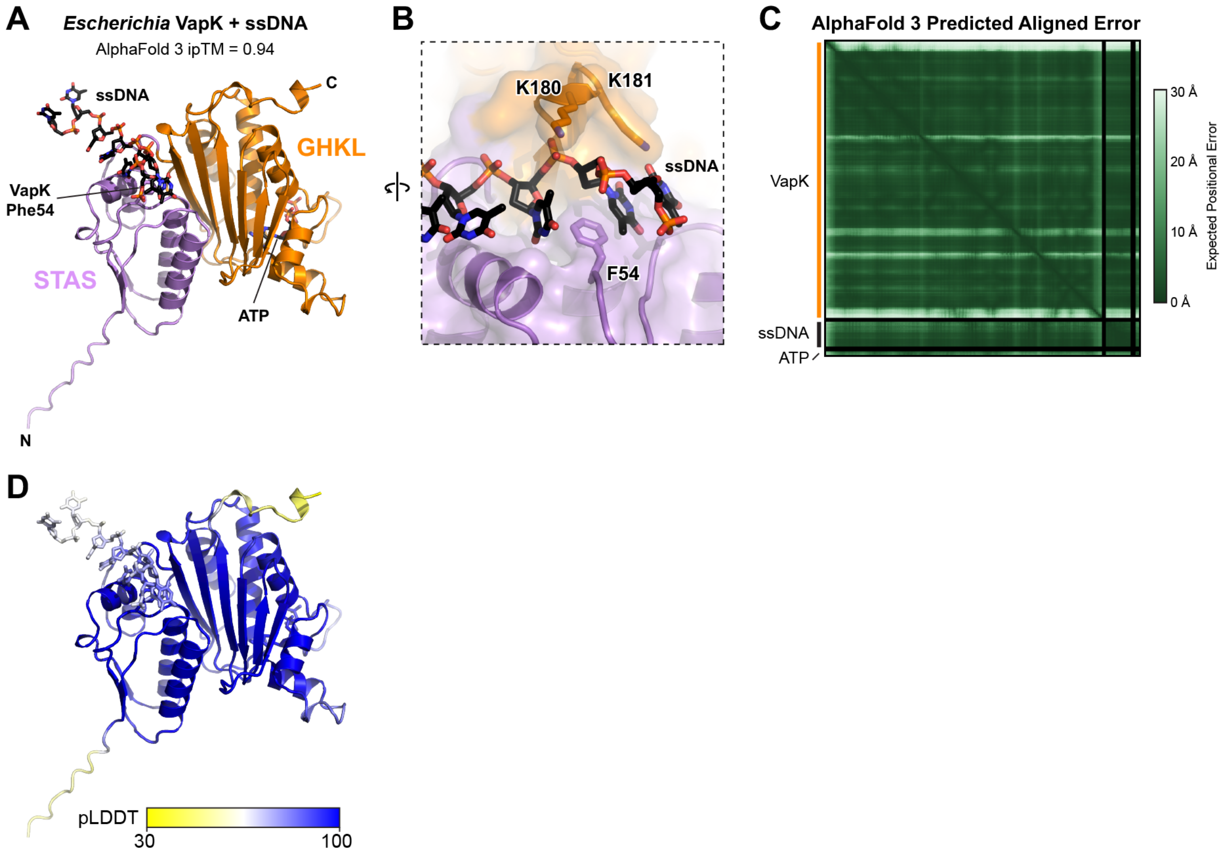

Appendix Figure S6 - AlphaFold 3 predicted model of the *Escherichia* VapK-ssDNA complex.

(A) AlphaFold 3 predicted model of a complex of *Escherichia* VapK (STAS domain pink, GHKL domain orange), a 7mer poly-T ssDNA (black), ATP (black), and Mg^{2+} (gray; not visible). VapK Phe54 is shown as sticks. **(B)** Closeup of ssDNA binding by VapK, with Phe54, Lys180, and Lys181 shown as sticks and labeled. **(C)** Predicted Aligned Error (PAE) plot for the model shown in panel (A). **(D)** View as in panel (A), colored by confidence (pLDDT).

Appendix Table S1. Crystallographic data collection and refinement statistics

	***E. roggenkampii* CapS**	***V. cholerae* CapS S58A form 1**	***V. cholerae* CapS S58A form 2**	***Escherichia* VapS-VapC**
**Data Collection**				
Synchrotron/Beamline	SSRL 12-1	SSRL 9-2	SSRL 9-2	APS 24ID-C
Date collected	5-29-2024	6-19-2024	6-19-2024	10-5-2025
Wavelength (Å)	0.97946	0.97946	0.97946	0.97905
Space Group	$P6_522$	$P3_121$	$C222_1$	H3
Unit Cell Dimensions (a,b,c) Å	55.83, 55.83, 184.51	59.17, 59.17, 426.20	79.28, 98.19, 279.00	159.08, 159.08, 176.20
Unit cell Angles (α,β,γ)°	90, 90, 120	90, 90, 120	90, 90, 90	90, 90, 120
Resolution (Å)*	38.01-1.55 (1.58-1.55)	51.25-2.38 (2.41-2.38)	61.68-1.84 (1.88-1.84)	74.22-3.40 (3.73-3.40)
I/σ*	17.6 (1.6)	4.8 (1.6)	12.3 (1.0)	5.0 (2.6)
R_{merge}*	0.101 (1.169)	0.157 (1.654)	0.092 (2.572)	0.352 (1.119)
R_{meas}*	0.107 (1.35)	0.173 (1.724)	0.099 (2.772)	0.386 (1.246)
$CC_{1/2}$*	0.999 (0.660)	0.994 (0.606)	0.995 (0.335)	0.985 (0.757)
Completeness %	99.9 (99.3)	99.8 (100.0)	99.9 (100.0)	57.5 (11.9)
No. of unique reflections*	25795 (1209)	36315 (1731)	93914 (4570)	13126 (655)
Redundancy*	17.1 (7.6)	4.7 (4.8)	7.0 (7.2)	5.9 (5.3)
**Refinement**				
Resolution (Å)	29.33-1.55	51.25-2.38	41.38-1.84	74.22-3.40
No. of reflections	25,705	36,296	93,496	13,104
working	24,405	34,468	88,812	12,457
free	1,300	1,828	4,684	647
R_{work} (%)	16.66 (28.22)	21.10 (27.23)	18.83 (34.67)	23.97 (34.58)
R_{free} (%)	19.02 (30.53)	26.93 (32.88)	21.70 (35.51)	26.41 (39.75)
No. of atoms				
total	3,017	14,256	14,794	10394
solvent	203	274	740	0
hydrogen	1,418	7,049	7,075	0
ligand	0	0	0	4 (Mg^{2+})
r.m.s.d. bond lengths (Å)	0.0063	0.0023	0.0016	0.0198
r.m.s.d. bond angles (°)	0.82	0.58	0.53	2.13
Ramachandran				
favored (%)	98.33	97.32	97.30	98.70
allowed (%)	1.67	2.68	2.70	1.30
disallowed (%)	0	0	0	0
MolProbity Score	1.18	1.15	1.03	1.65
MolProbity ClashScore	3.91	2.43	1.57	13.78
SBGrid Data Bank ID	1238	1239	1240	1241
Protein Data Bank ID	9Z71	9Z72	9Z73	9Z7O

*Values in parentheses are for highest-resolution shell.

Appendix Table S2. Proteins used in this study

Protein	Database and accession number	Sequence
E. roggenkampii CapK	NCBI WP_001567866.1	MLFDTEKIKELLRESPGLTGKQIAKTLGYADKSALNSFLYSHAEGLKRVEWKWYVDDEYVLVLDGDAWIDENIFEANLSASGCLLSASARRCSISFPESCSILLAAGARIIALANQAAYSGKSIELDFSKCPSAKNYLNRLGFFDHLHPDVLVKPERPTNSRAQRYQGNSDNLVEIASIDLDDFDNSIPVKLTKKFVVHAGQKYYMAVFTIFSELIGNVRDHSESPIPGFAALQLYKGKRRHIQTVISDSGLGIATTLKRNLKKHYPEIFEELESSGEDADLFLVTHALKNGGLSQFGSAPDEAARGLGLKRSQDLAAKYDAVVLVRQPDFELRITYKNGVITEITSKKRLALIKGTQVCFDFFLGYD
E. roggenkampii CapS	NCBI WP_001567865.1	MKVKLLELTENNNHPFGNVLGREVFKRLQNVVDSNPGCKSFEISLEGIVATDSSFPRESVIALAKQLCGEKYFFITDVSSTDLIDNWDYAAIAKQQSMIVVLAGGVRVIGPEAKSSTKALLDVVLGRNGVSTANVAKSLNISVQNASTRLKKLSSEGVIMRSEVSSPTGGIEFIYSGPNISA
V. cholerae CapK	NCBI WP_160230705.1	MLKAISELLDESPGLKGRQIAKELGLDKSQVNSFLHKNQDTFVKNSNHEWCLIRAQHVEIDFATGWIDDKAFESAIGKLAYQKDANKITFRFGIDCKFLLIALARFLALANQLAANGKDVVMDTTACPNTRGFFSRNGFFDYLNQSVTCLPERPVLSAAKTYRDNSDTLVELGEIAQPTQNKELVIRLGDRFVEHSSASYFLAAKTVFSELVGNVTDHSESKIPGLAGLQVYRPYNKPKHIQTVISDSGLGIAATLRTTLQSEHPKLYAQFSAETVENDIALVQKAFTSGEVSRFGKGRGLGFKSSREHASKEKVIIVIRQLTFSLALEYAKGQLINVSEDRGLVPITGTHICFDFYVDNF
V. cholerae CapS	NCBI WP_046127252.1	MKQLTHKILLSEVVGSDHAFGNDEGSEAYVKIKKIVDGHPSCDIFAISLEGIRFTDASFPRESVISLAKALKGEKGFYLSNVPSRDLLDNWSYGATAKDQPLLVKSDSGYEVLGVKLSATVKELLDFVIAKKTVTSSHVSKHFDISAQNASGRLKKLHATGLVLGQKEVAESGGLEFVYRSIL
Escherichia VapK	NCBI WP_135559716.1	MFNTKTGLTIMIPTLNDGEGDFMRLFMIYKQVMETDAPQITFIFTHCRFLRPNAVAFLGGTIRSLQKKGVTVFVDWKSIPSAVMASLKQNTFCSKLGYSSHTNPGHAIPYREDPKEDANSILDYLTQNWIGKGWVKVSEPLRDAIAGKVWEIYANSFEHSKSKIGVFSCGQHFIKKNELVLSVVDFGVGIPHNVREFLSSDARAASLSAESCLKWACQSGNTTATANGVPRGLGLHLLKELVRVNNGKLELYSHNGYVKMSSQGEEYKTQPFYFEGTILNITLLCDEKYYRLTSELG
Escherichia VapS	NCBI WP_135559717.1	MKILIKDFIGTRCILKEDGQKLFEEISRHLESKDEVILDFSNVKMFASPFFNYSIGQLFNKFSENEIRNNLHLDNLEVVGHSIIERVVENASRFKSDLDYKKIVDEILEQQARESD
Escherichia VapC	NCBI WP_135559718.1	MAFSGVILANVFNIGNYTPTKDDKFLVDTNVWYWMTYTKGIPSNRQYLNTYIKFISDCISKESQLFHSGLSLAELAHIIESTEREIHEATIKSRIMTKEFRYKYAKERQLAMKEVEVSWAQVKQIAPQVDLTICQDLTDSCASKMTSNTLDGYDLMIHETMLKHGITNIITDDGDYTSVPGINVFTINKNVIVSAATQKKLMN
