# Supplementary figures and images for: A DNA damage-activated kinase phosphorylates a transcriptional repressor to control bacterial immune pathway expression
Source: EMBO J. 2026 Jun 9;45(14):5079–100. doi: 10.1038/s44318-026-00831-y (PMC13373217; doi:10.1038/s44318-026-00831-y)

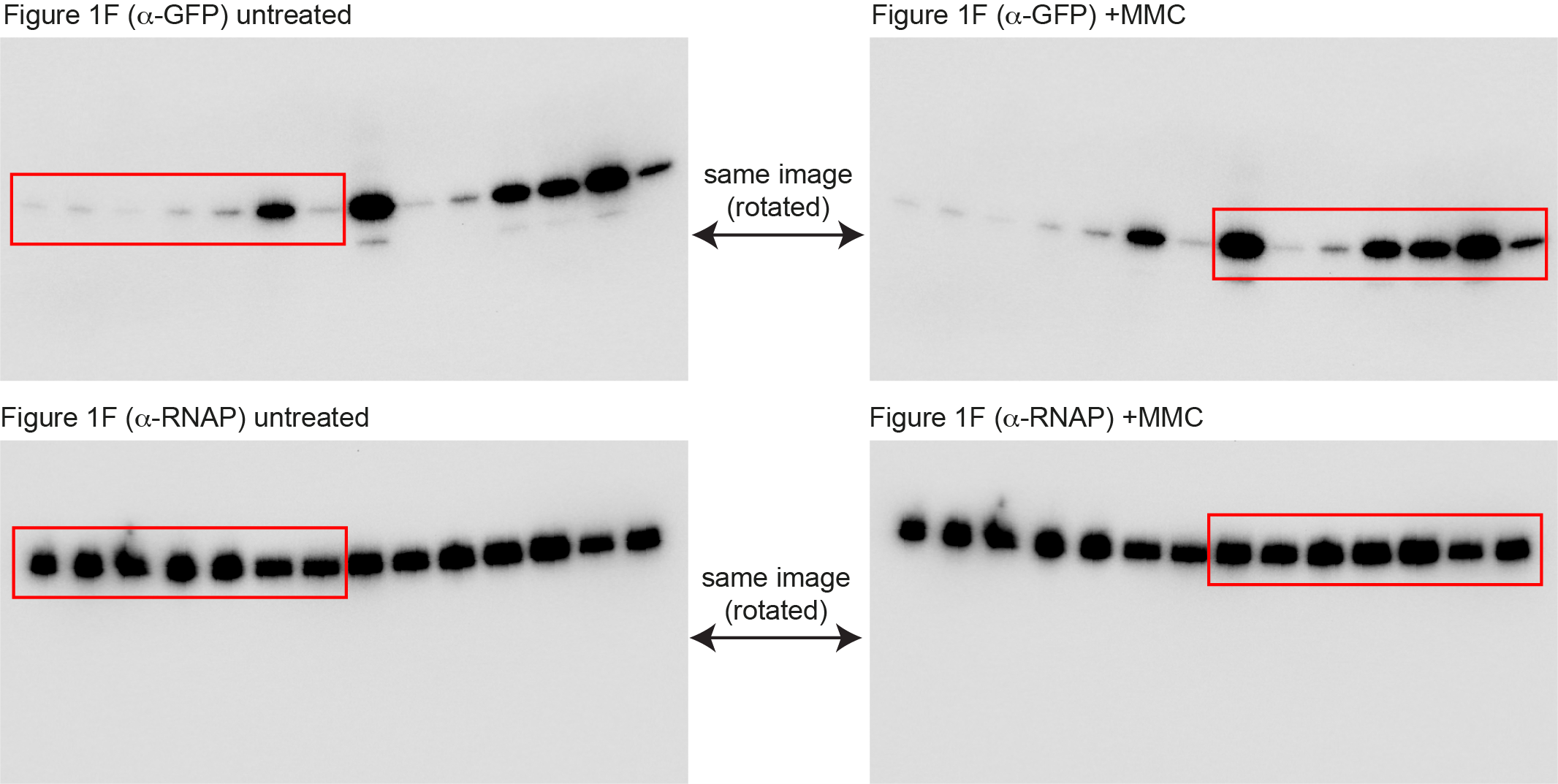

Supplement: Supplementary file 7 — Source data Fig. 1 [file 44318_2026_831_MOESM7_ESM.zip › Figure1/1F/1F_blots.png]

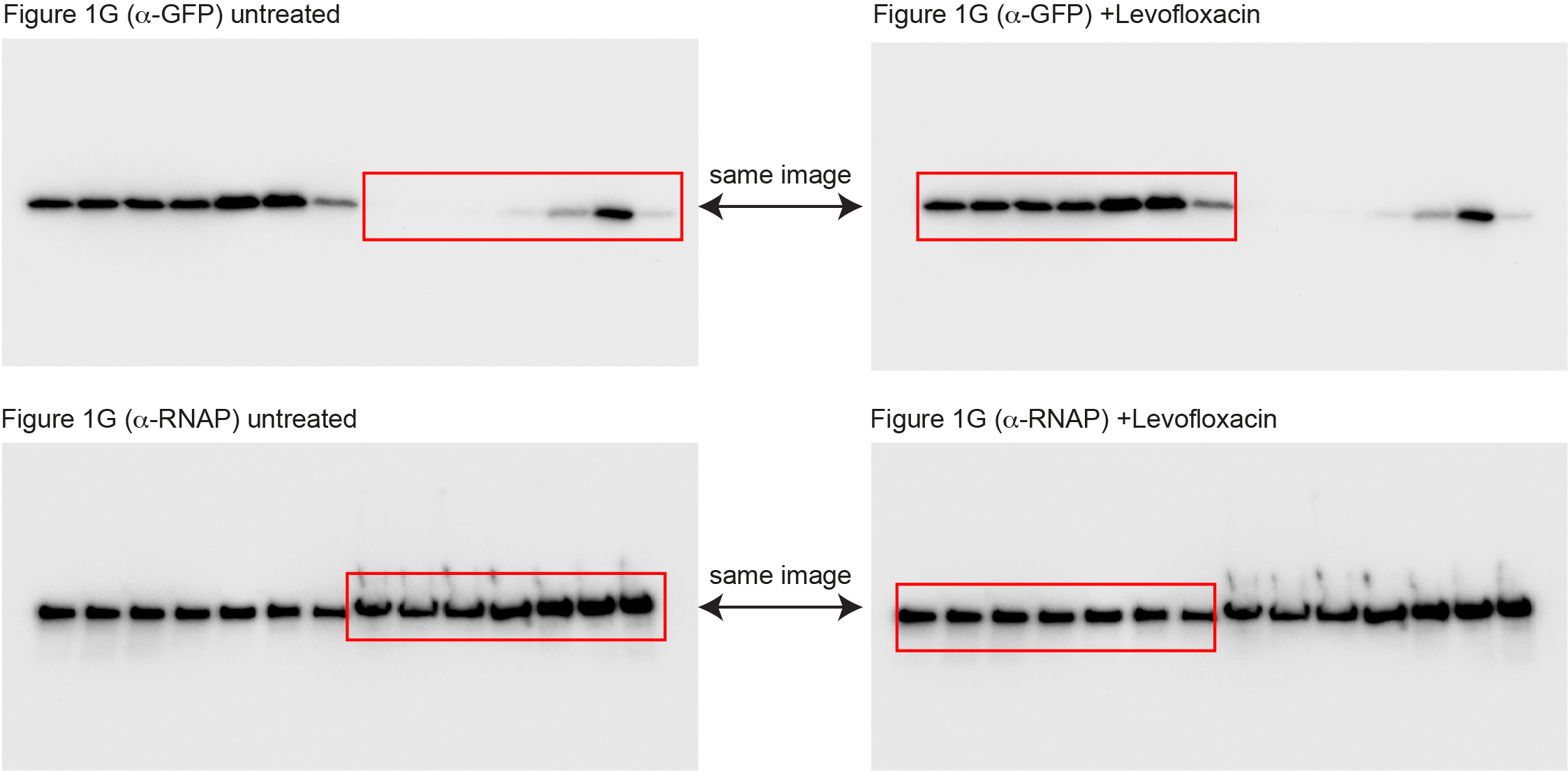

Supplement: Supplementary file 7 — Source data Fig. 1 [file 44318_2026_831_MOESM7_ESM.zip › Figure1/1G/1G_blots.png]

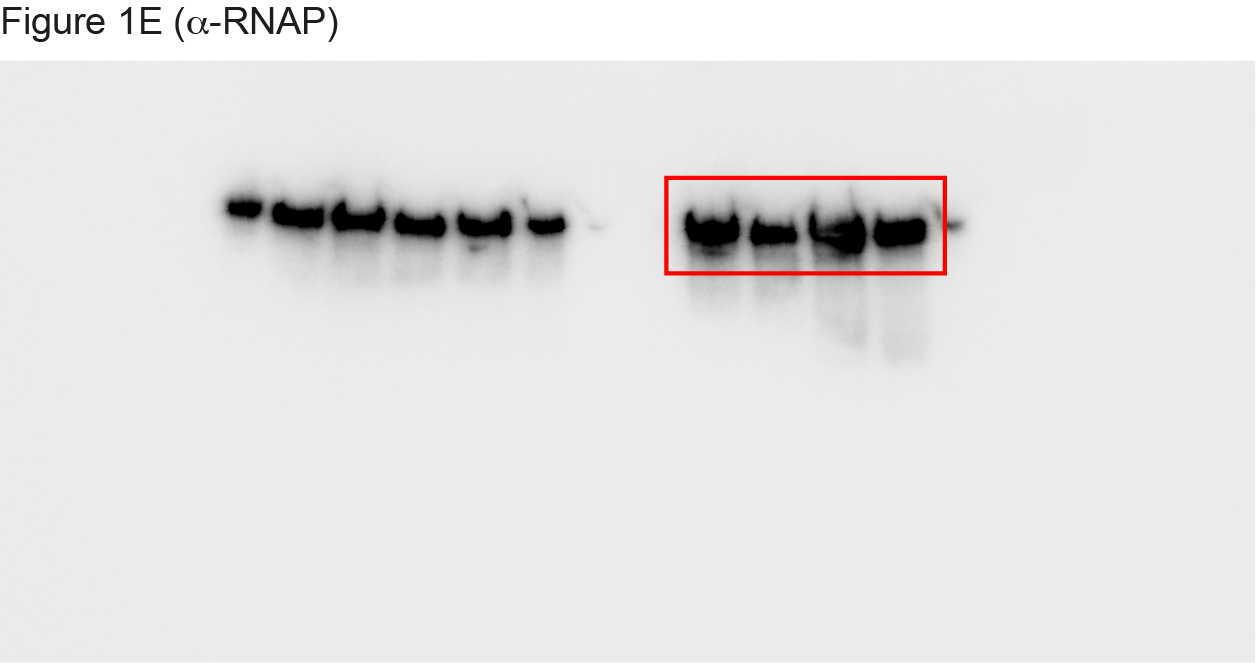

Supplement: Supplementary file 7 — Source data Fig. 1 [file 44318_2026_831_MOESM7_ESM.zip › Figure1/1E/1E_anti-RNAP.png]

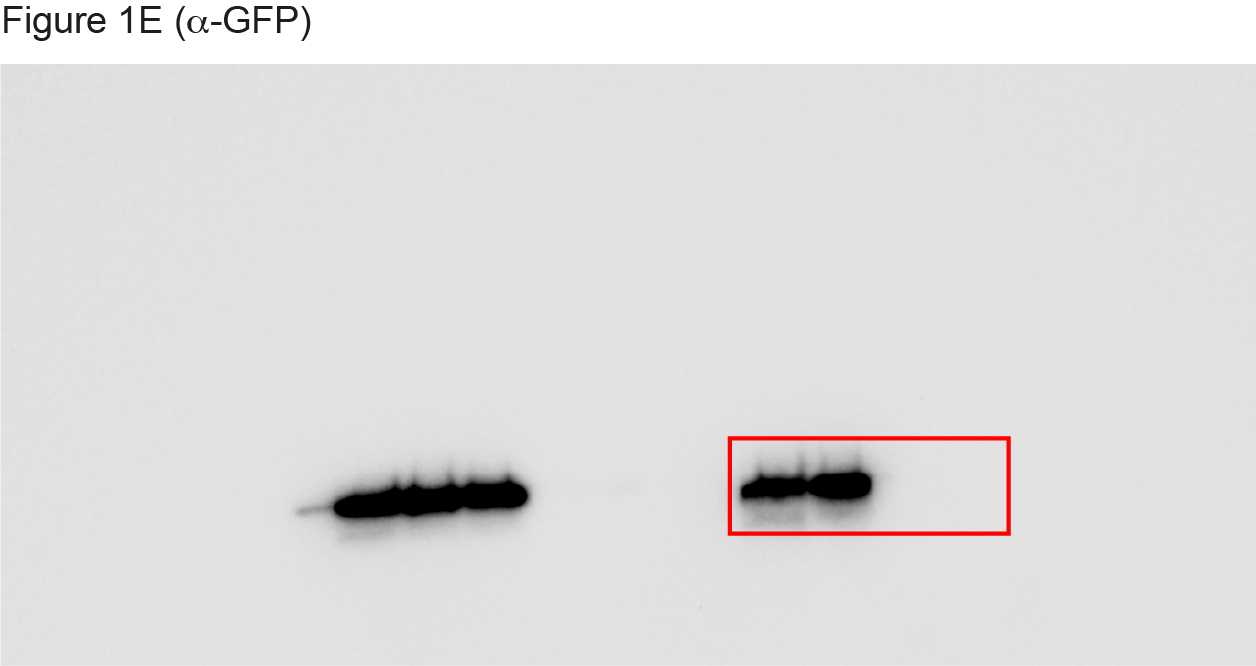

Supplement: Supplementary file 7 — Source data Fig. 1 [file 44318_2026_831_MOESM7_ESM.zip › Figure1/1E/1E_anti-GFP.png]

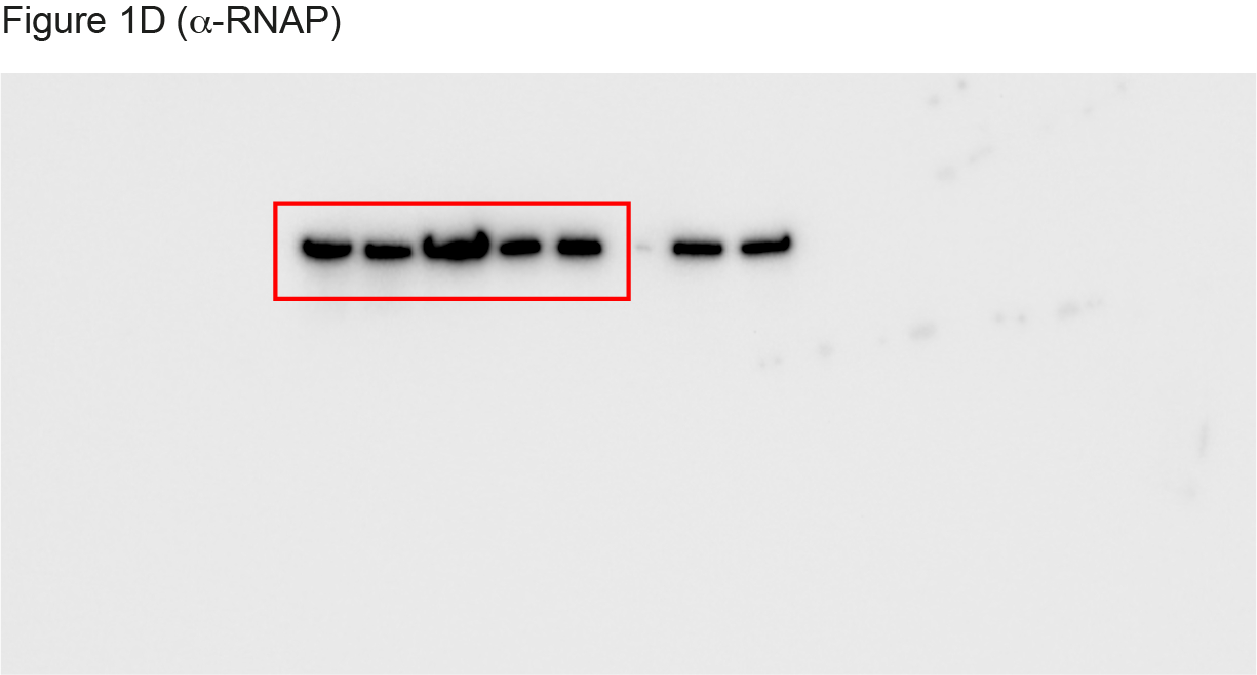

Supplement: Supplementary file 7 — Source data Fig. 1 [file 44318_2026_831_MOESM7_ESM.zip › Figure1/1D/1D_anti-RNAP.png]

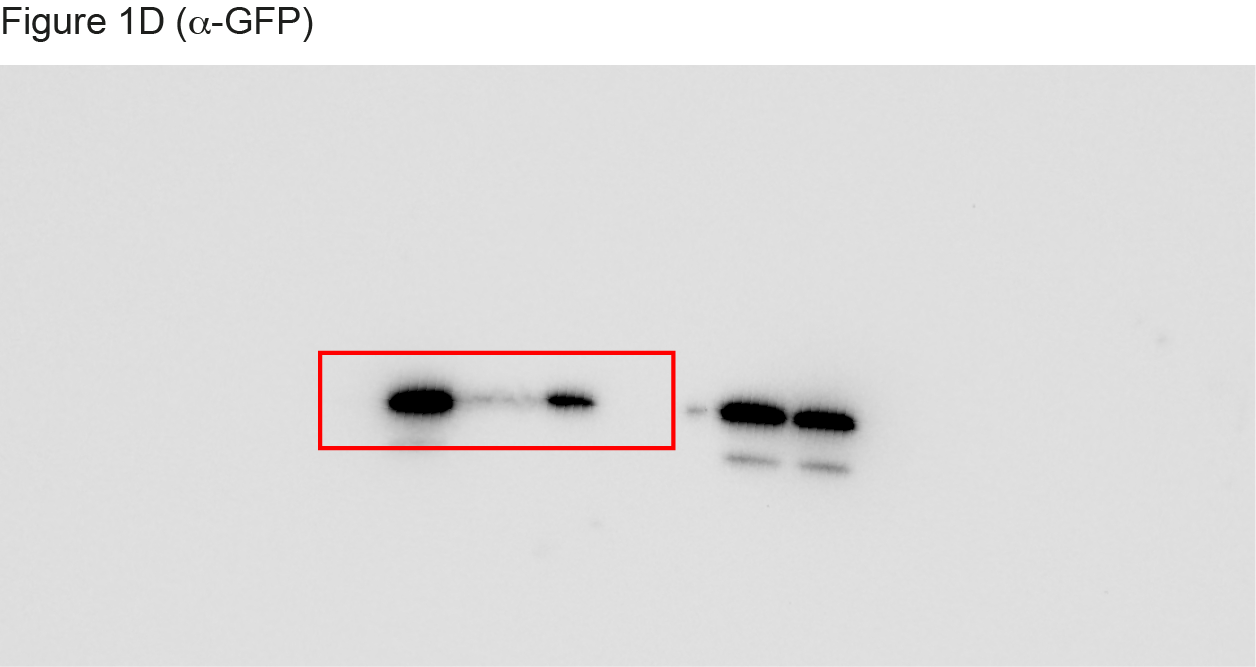

Supplement: Supplementary file 7 — Source data Fig. 1 [file 44318_2026_831_MOESM7_ESM.zip › Figure1/1D/1D_anti-GFP.png]

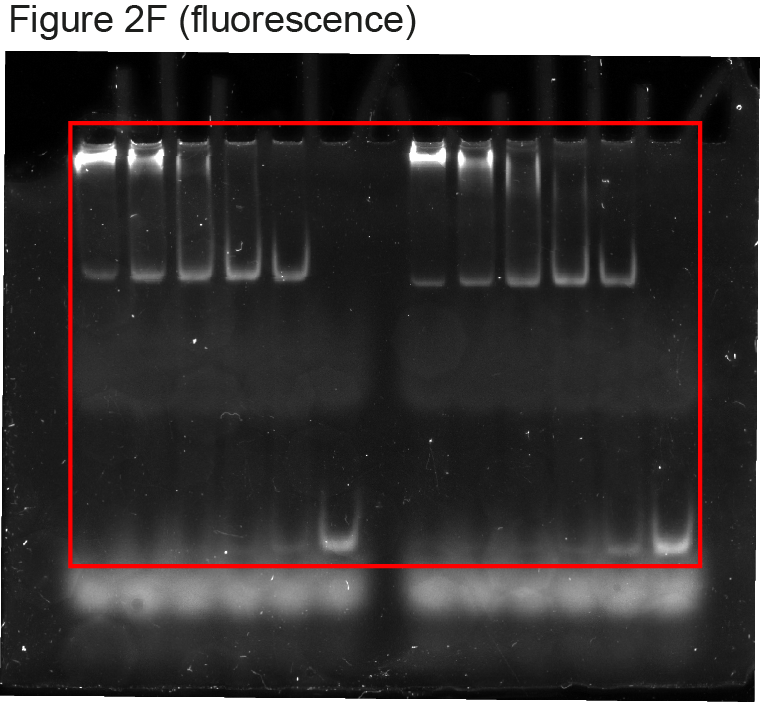

Supplement: Supplementary file 8 — Source data Fig. 2 [file 44318_2026_831_MOESM8_ESM.zip › Figure2/2F/2F_fluorescence-gel.png]

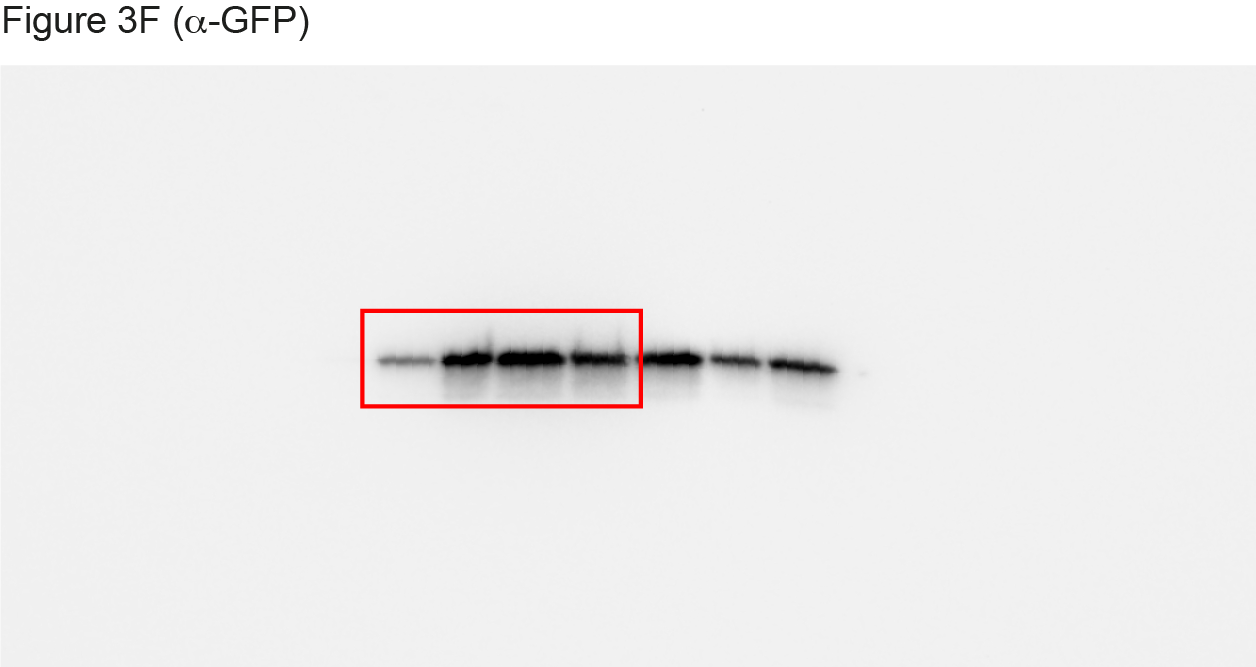

Supplement: Supplementary file 9 — Source data Fig. 3 [file 44318_2026_831_MOESM9_ESM.zip › Figure3/3F/3F_anti-GFP.png]

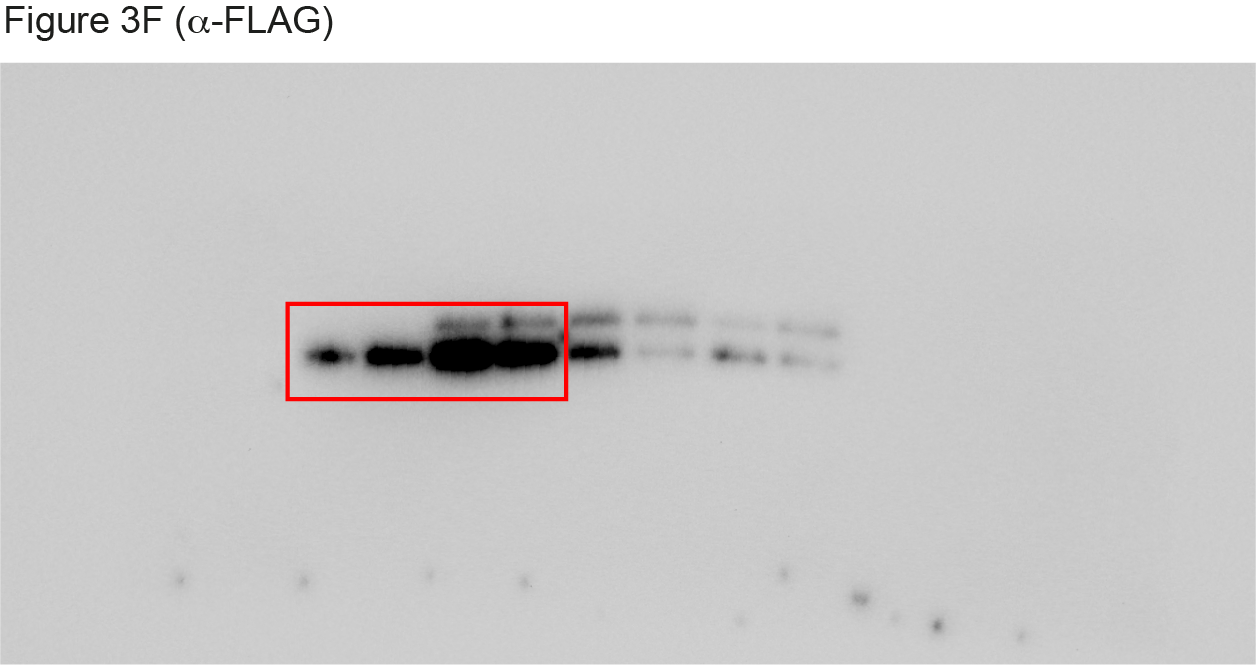

Supplement: Supplementary file 9 — Source data Fig. 3 [file 44318_2026_831_MOESM9_ESM.zip › Figure3/3F/3F_anti-FLAG.png]

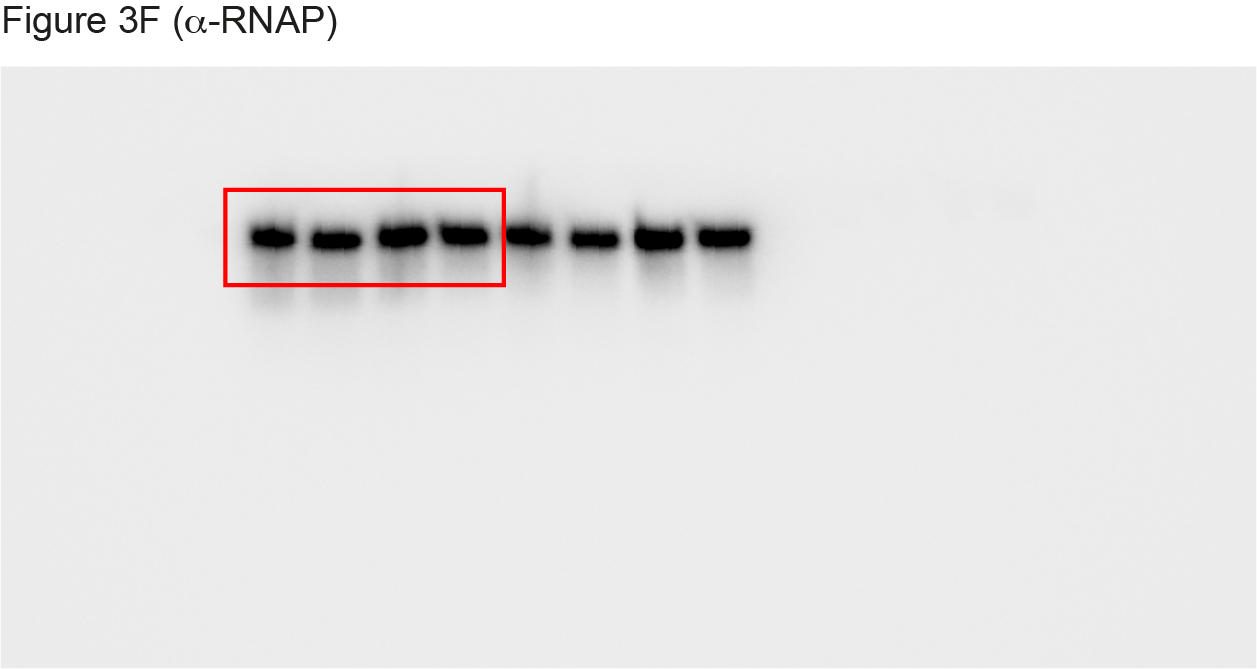

Supplement: Supplementary file 9 — Source data Fig. 3 [file 44318_2026_831_MOESM9_ESM.zip › Figure3/3F/3F_anti-RNAP.png]

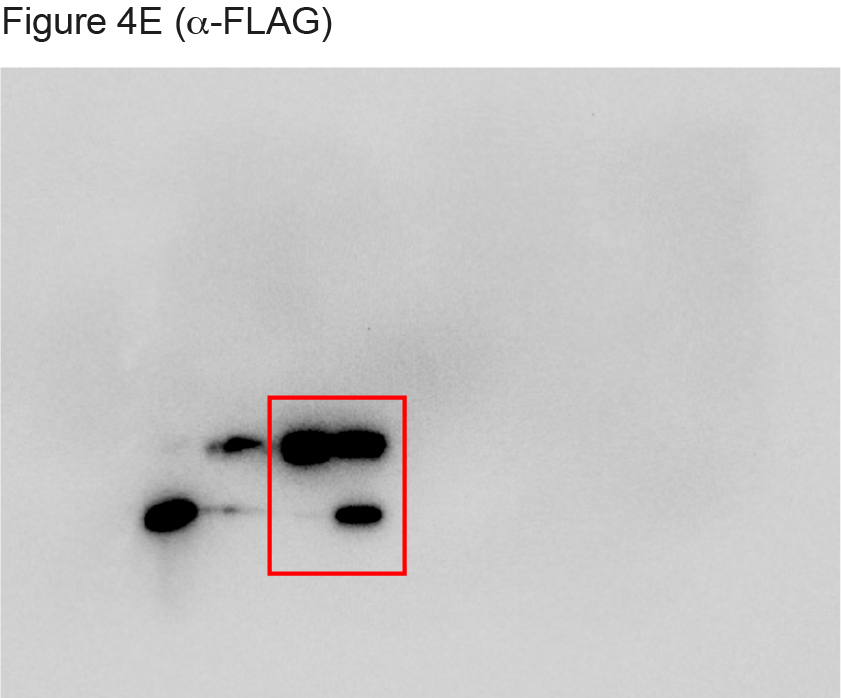

Supplement: Supplementary file 10 — Source data Fig. 4 [file 44318_2026_831_MOESM10_ESM.zip › Figure4/4E/4E_anti-FLAG.png]

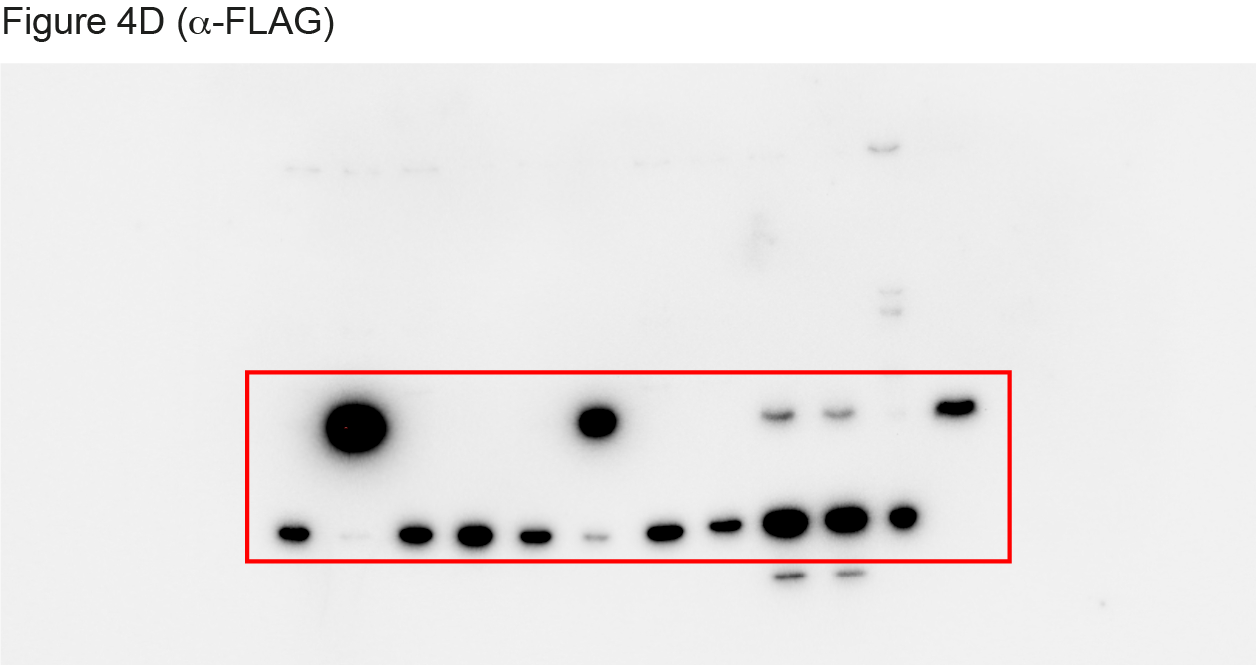

Supplement: Supplementary file 10 — Source data Fig. 4 [file 44318_2026_831_MOESM10_ESM.zip › Figure4/4D/4D_anti-FLAG.png]

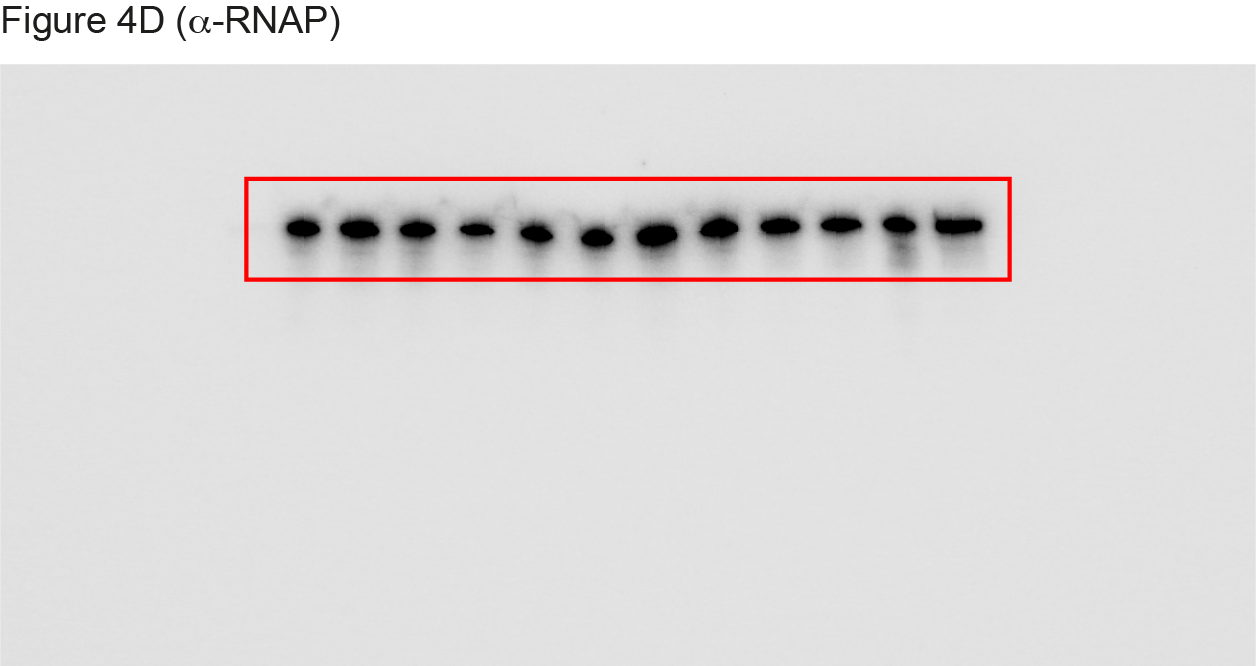

Supplement: Supplementary file 10 — Source data Fig. 4 [file 44318_2026_831_MOESM10_ESM.zip › Figure4/4D/4D_anti-RNAP.png]

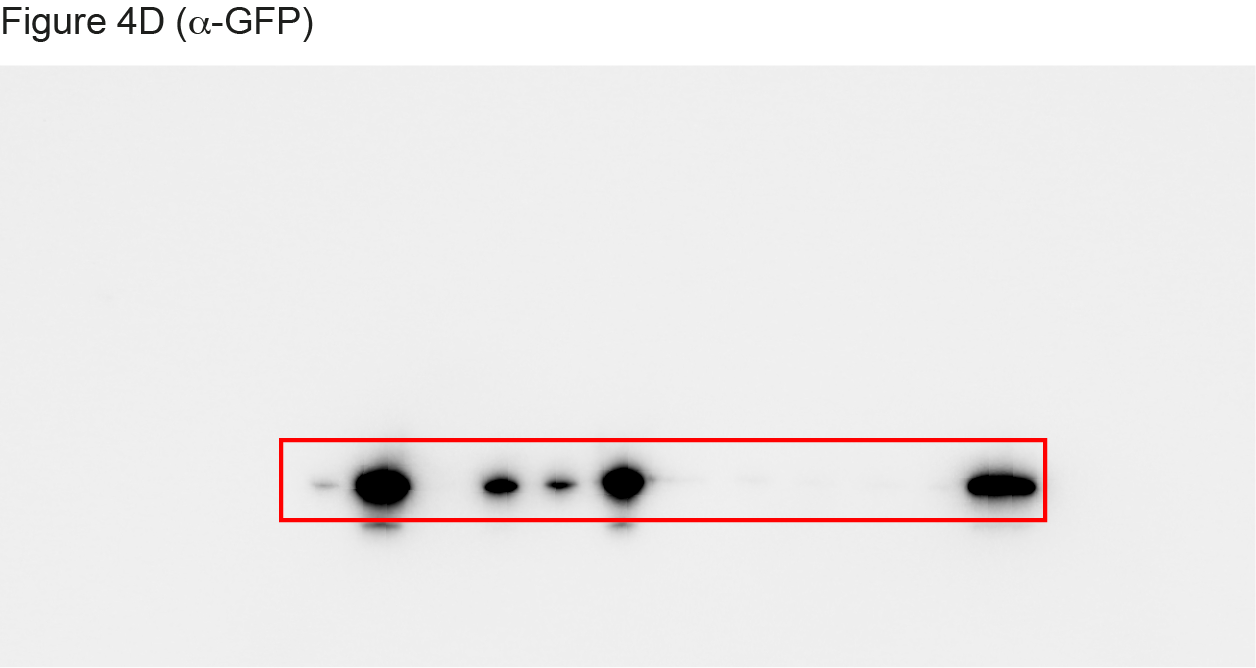

Supplement: Supplementary file 10 — Source data Fig. 4 [file 44318_2026_831_MOESM10_ESM.zip › Figure4/4D/4D_anti-GFP.png]

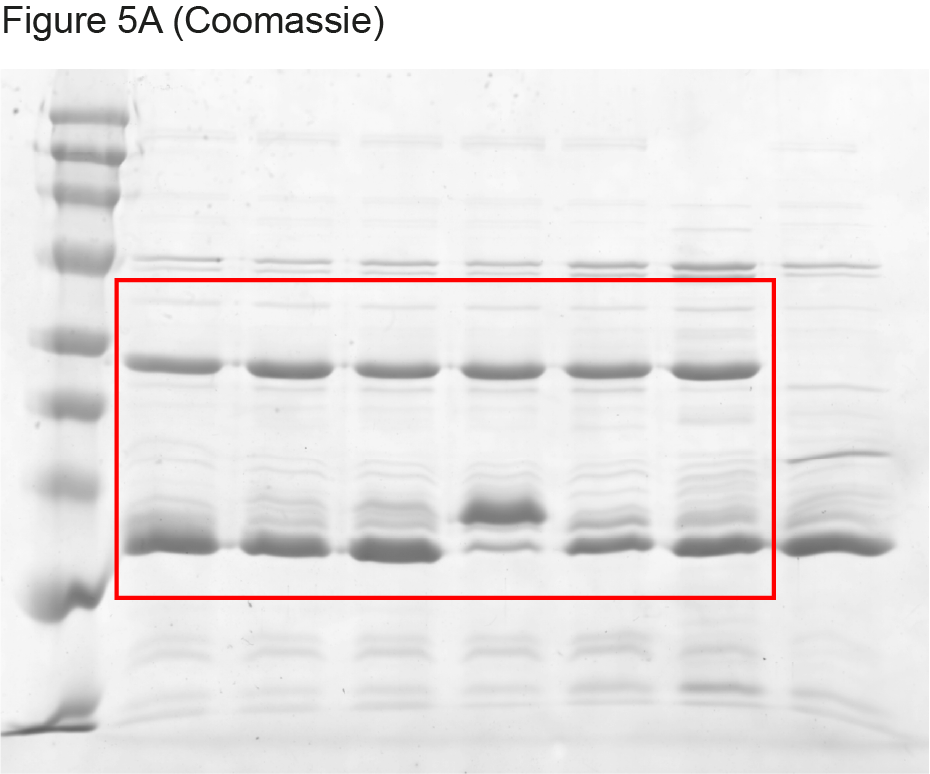

Supplement: Supplementary file 11 — Source data Fig. 5 [file 44318_2026_831_MOESM11_ESM.zip › Figure5/5A/5A_Coomassie.png]

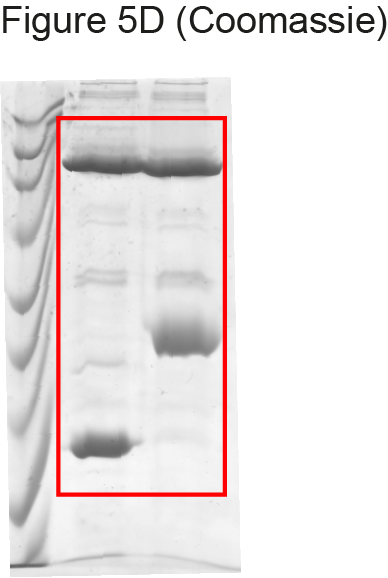

Supplement: Supplementary file 11 — Source data Fig. 5 [file 44318_2026_831_MOESM11_ESM.zip › Figure5/5D/5D_coomassie.png]

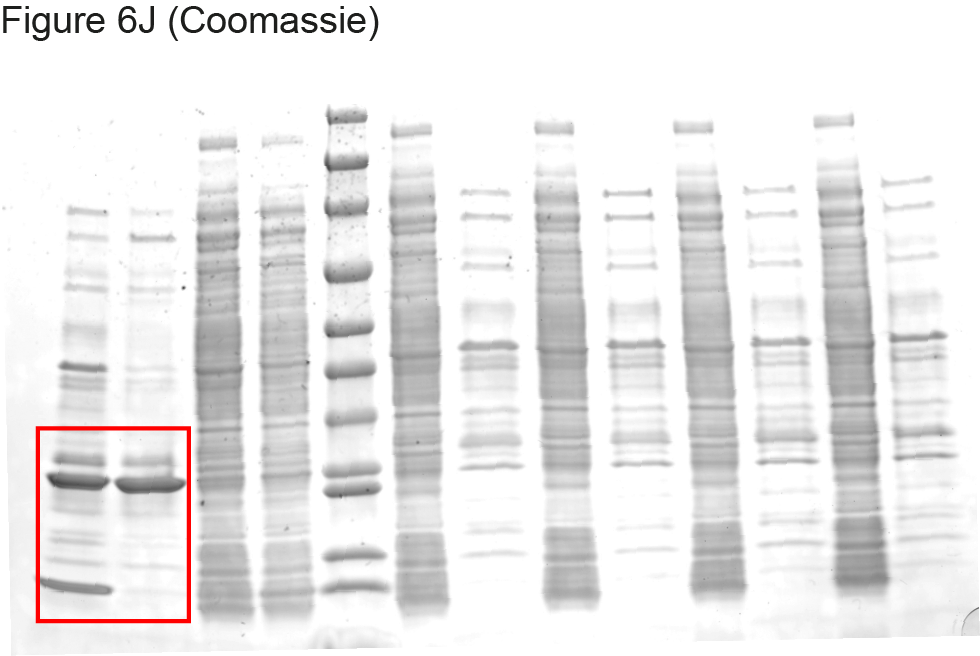

Supplement: Supplementary file 12 — Source data Fig. 6 [file 44318_2026_831_MOESM12_ESM.zip › Figure6/6J/6J_Coomassie.png]

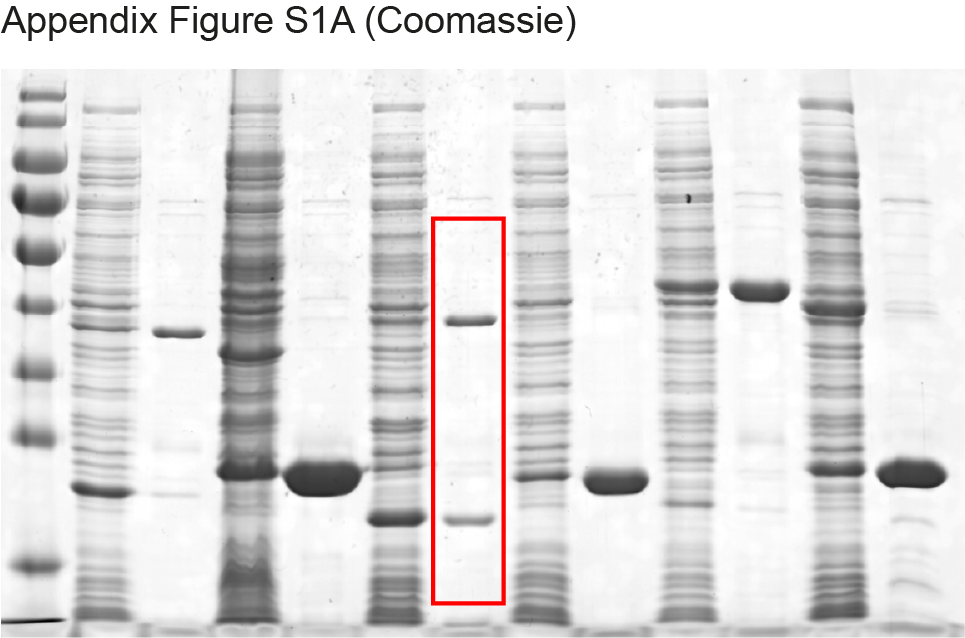

Supplement: Supplementary file 13 — Source Data for Expanded View and Appendix Figures [file 44318_2026_831_MOESM13_ESM.zip › Source_Data_for_Expanded_View_and_Appendix/AppendixFigureS1/S1A/S1A_gel.png]

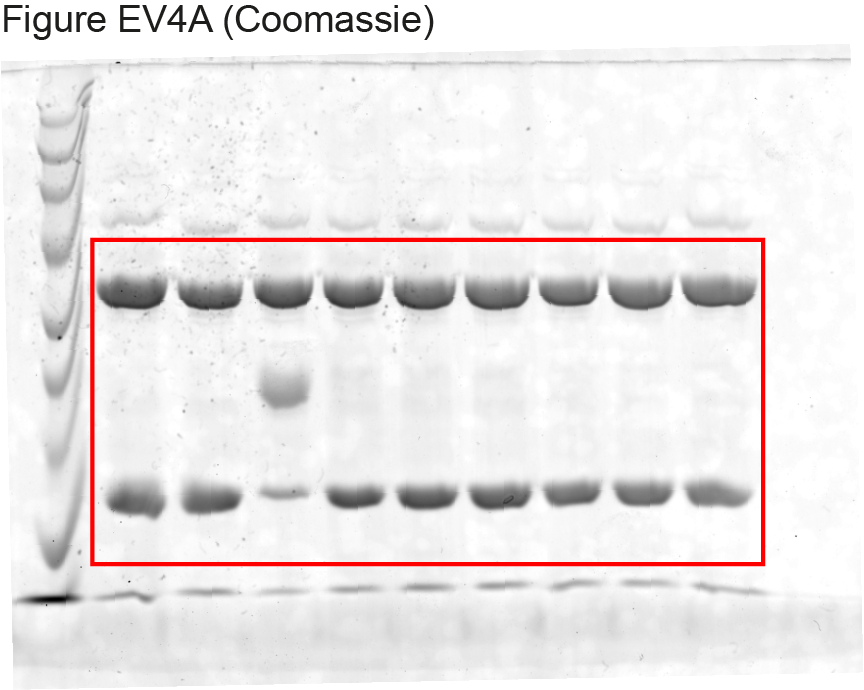

Supplement: Supplementary file 13 — Source Data for Expanded View and Appendix Figures [file 44318_2026_831_MOESM13_ESM.zip › Source_Data_for_Expanded_View_and_Appendix/FigureEV4/EV4A/EV4A_coomassie.png]
