# Supplementary material for: A DNA damage-activated kinase phosphorylates a transcriptional repressor to control bacterial immune pathway expression
Source: EMBO J. 2026 Jun 9;45(14):5079–100. doi: 10.1038/s44318-026-00831-y (PMC13373217; doi:10.1038/s44318-026-00831-y)
Supplement: Supplementary file 14 — Expanded View Figures [file 44318_2026_831_MOESM14_ESM.pdf]

Expanded View Figures

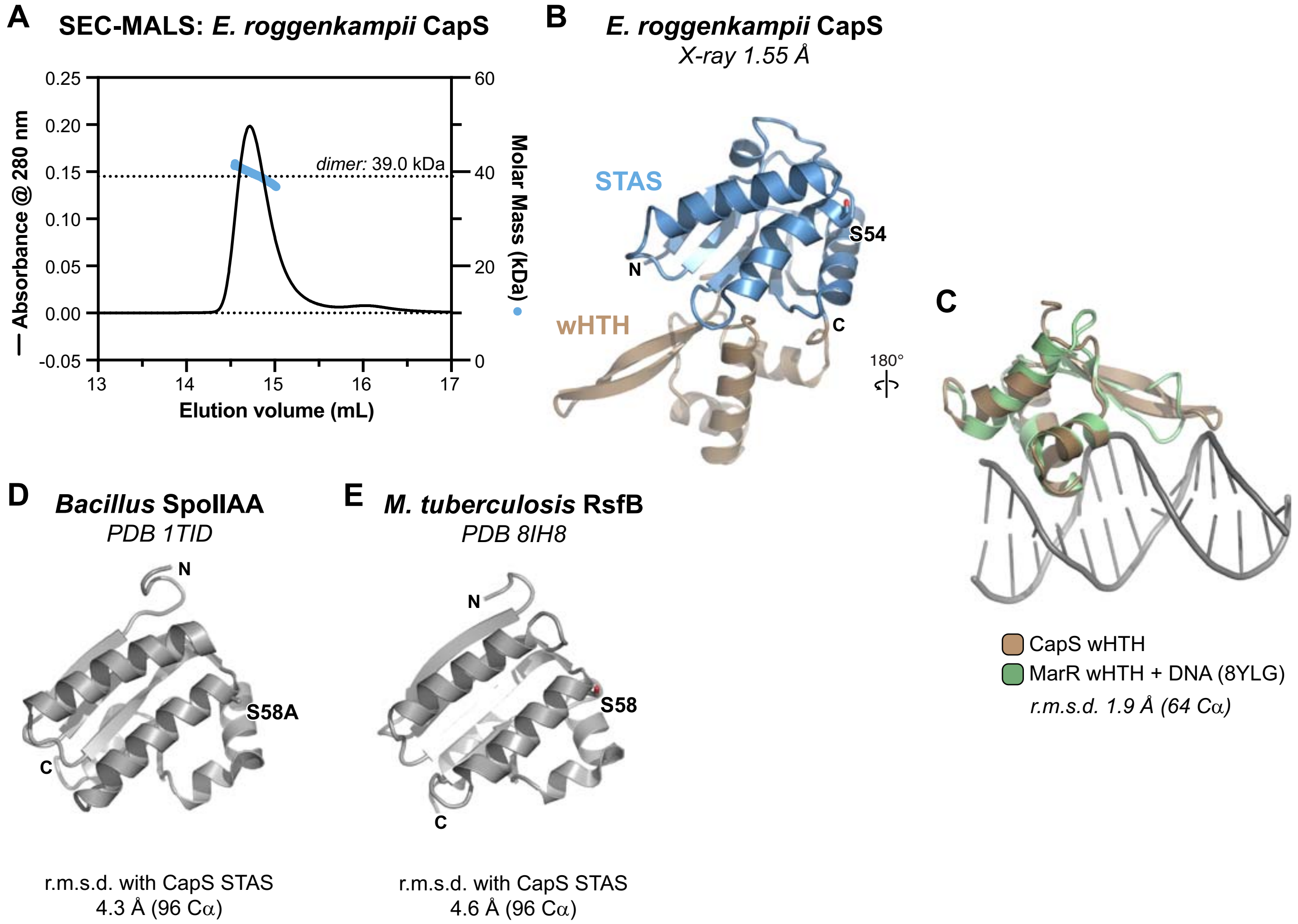

Figure EV1. Structure of *E. roggenkampii* CapS.

(**A**) Size exclusion chromatography coupled to multi-angle light scattering (SEC-MALS) analysis of purified *E. roggenkampii* CapS. This experiment was performed once. (**B**) Structure of *E. roggenkampii* CapS, with N-terminal STAS domain blue and C-terminal wHTH domain brown. The N- and C-termini of the STAS domain are labeled, and Ser54 is shown as sticks and labeled. (**C**) Overlay of *E. roggenkampii* CapS (brown) with a crystal structure of MarR bound to DNA (green; PDB ID 8YLG) (Song et al, 2024); only the wHTH domains of both proteins are shown. (**D**) Structure of *Geobacillus stearothermophilus* SpoIIAA (from PDB ID 1TID) (Masuda et al, 2004), with Ser58 (mutated to alanine) shown as sticks and labeled. The overall Cα r.m.s.d. (root mean squared deviation) with CapS is 4.3 Å over 96 residues. (**E**) Structure of *Mycobacterium tuberculosis* RsfB (from PDB ID 8IH8; unpublished), with Ser58 shown as sticks and labeled. The overall Cα r.m.s.d. with CapS is 4.6 Å over 96 residues.Source data are available online for this figure.

© The Author(s)

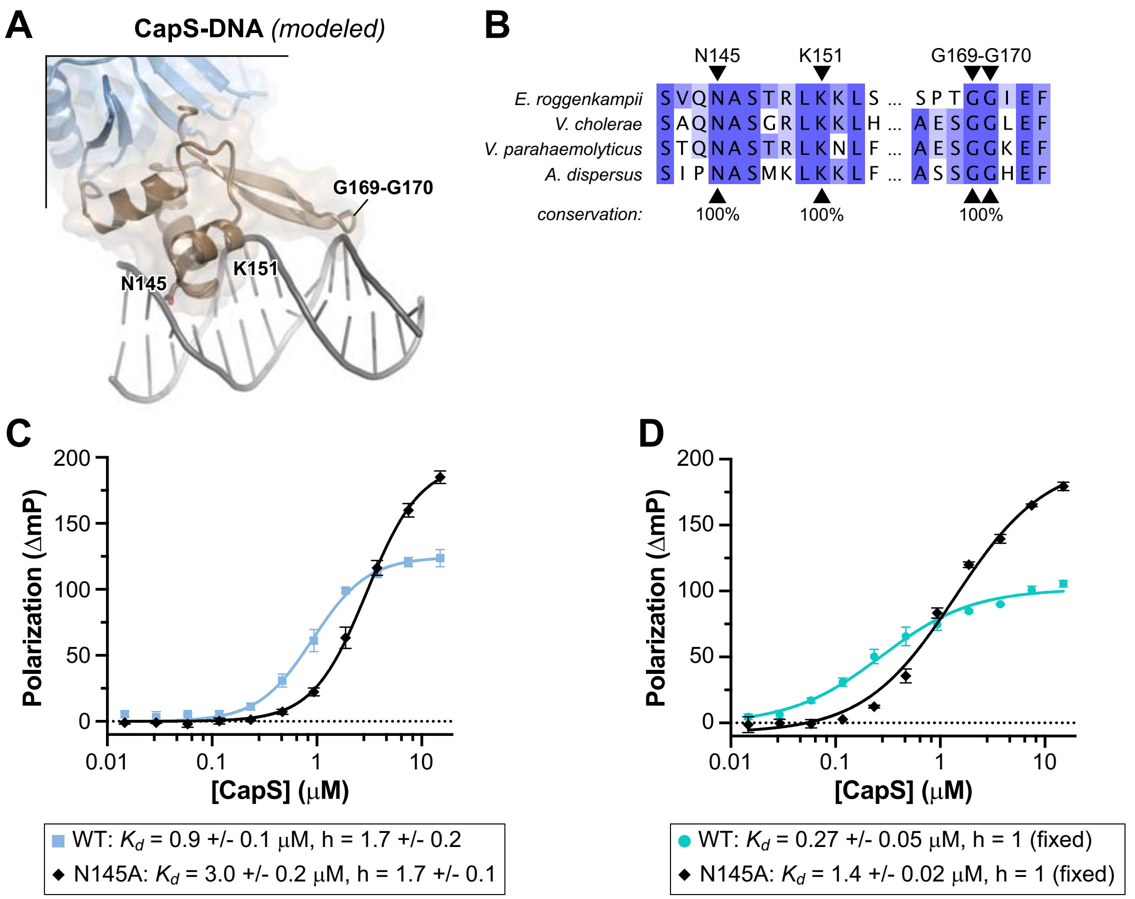

Figure EV2. DNA binding by CapS.

(**A**) Model of the CapS wHTH domain (brown) bound to DNA (gray), generated by overlaying the CapS wHTH domain with a DNA-bound structure of MarR (PDB ID 8YLG; Fig. EV1C) (Song et al, 2024). Conserved residues predicted to bind DNA (N145, K151, G169, and G170) are shown as sticks and labeled. (**B**) Section of sequence alignment of the four CapS proteins shown in Fig. 1A (*E. roggenkampii* (NCBI WP_001567865.1), *V. cholerae* (NCBI WP_046127252.1), *V. parahaemolyticus* (IMG 2662856252), showing conservation of predicted DNA-binding residues. (**C**) Fluorescence polarization DNA-binding assay with *E. roggenkampii* CapS (wild-type shown in light blue squares, N145A shown in black diamonds) binding a 24-bp narrow palindrome DNA. Each datapoint is an average of three technical replicates, and arrow bars indicate the mean ± standard deviation (error bars not shown if they are smaller than the datapoint itself). DNA-binding curves were fit with a cooperative binding model (h: Hill coefficient). The data presented in panels (**C**, **D**) were representative of three independent trials. (**D**) Fluorescence polarization DNA-binding assay with *E. roggenkampii* CapS (wild-type shown in light green circles, N145A shown in black diamonds) binding a 24-bp wide palindrome DNA. Each datapoint is an average of three technical replicates, and arrow bars indicate the mean ± standard deviation (error bars not shown if they are smaller than the datapoint itself). DNA-binding curves were fit with a single-site binding model.Source data are available online for this figure.

 © The Author(s)

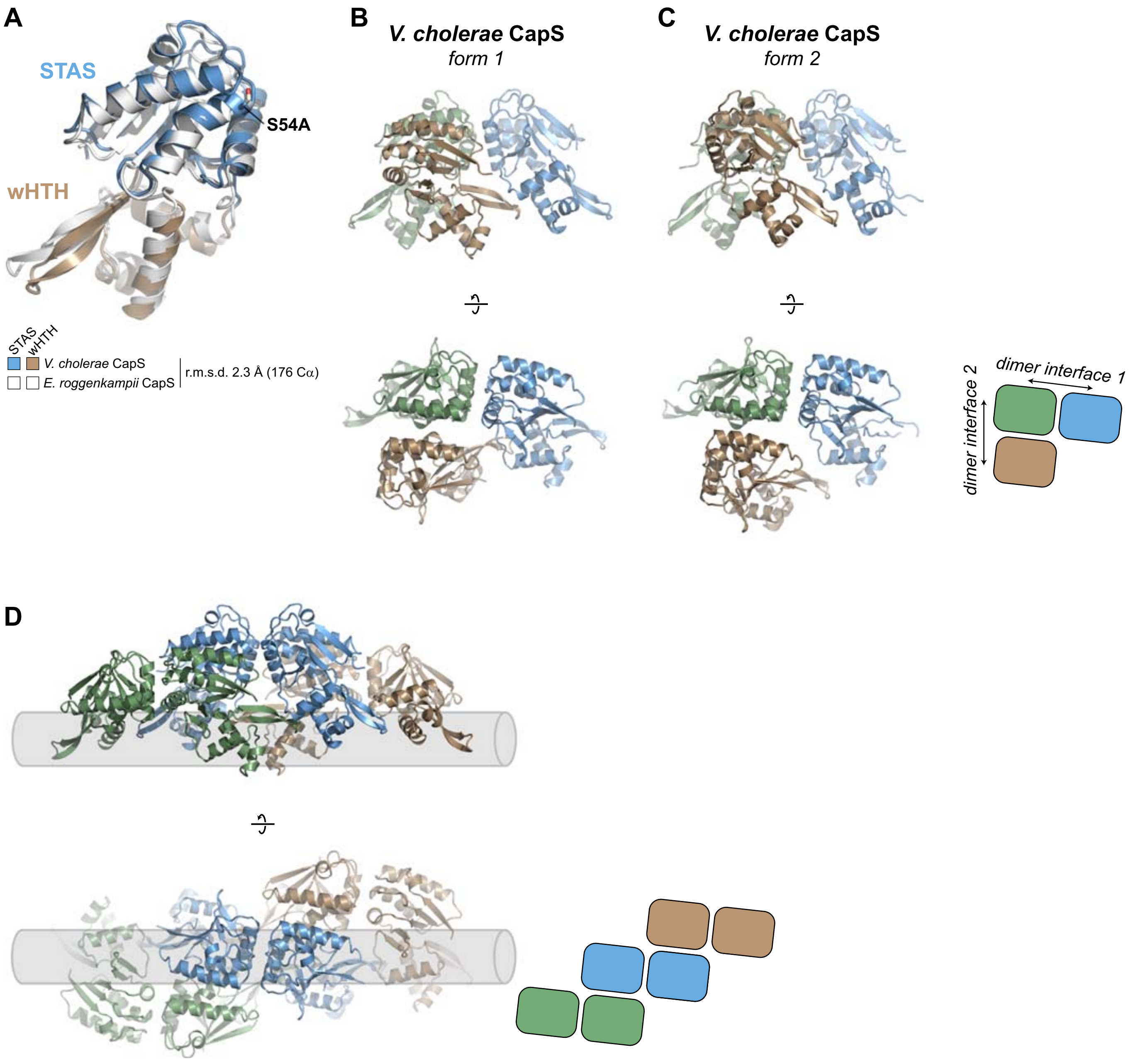

Figure EV3. Structure of *V. cholerae* CapS (S58A).

(**A**) Overlay of *V. cholerae* CapS (STAS domain blue; wHTH domain brown) from crystal form 1 with *E. roggenkampii* CapS (Cα r.m.s.d. 2.3 Å over 176 residues). (**B**) Two views of three non-crystallographic symmetry-related *V. cholerae* CapS protomers in crystal form 1, showing dimer interface 1 (green-blue) and dimer interface 2 (green-brown). (**C**) Two views of three non-crystallographic symmetry-related *V. cholerae* CapS protomers in crystal form 2, showing dimer interface 1 (green-blue) and dimer interface 2 (green-brown). (**D**) Two views of a *V. cholerae* CapS filament (six protomers) assembled from a combination of crystallographic and non-crystallographic symmetry. Three dimers assembled via interface 1 are colored green, blue, and brown, respectively. Each of these dimers associates with other dimers through interface 2. Shown in gray is a theoretical DNA duplex positioned such that all six CapS wHTH domains could bind it.

© The Author(s)

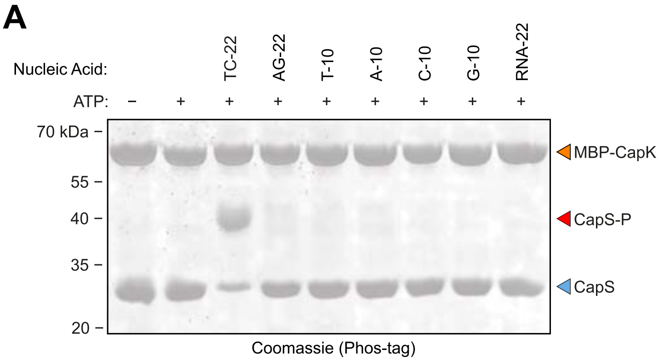

B

```
TC-22:  TCTTTTTCTCCCCCTCTTTCCC
AG-22:  GGAGAAGAAGGAGAAGGAGGGA
T-10:   TTTTTTTTTT
C-10:   CCCCCCCCCC
A-10:   AAAAAAAAAA
G-10:   GGGGGGGGGG
RNA-22: AGACUGCAAUCGAUUACAUCUU
```

Figure EV4. Single-stranded DNA activates CapK.

(**A**) Phos-tag SDS-PAGE gel analysis of phosphorylation of CapS by MBP-tagged CapK, in the presence or absence of ATP and selected single-stranded nucleic acids. The data presented were representative of three independent trials. (**B**) Key showing the sequences of all nucleic acids used in panel (**A**). All are DNA except for “RNA-ss” which is RNA.Source data are available online for this figure.

 © The Author(s)
