# Supplementary material for: A DNA damage-activated kinase phosphorylates a transcriptional repressor to control bacterial immune pathway expression
Source: EMBO J. 2026 Jun 9;45(14):5079–100. doi: 10.1038/s44318-026-00831-y (PMC13373217; doi:10.1038/s44318-026-00831-y)
Supplement: Supplementary file 2 — Peer Review File [file 44318_2026_831_MOESM2_ESM.pdf]

THE EMBO JOURNAL

A DNA damage-activated kinase phosphorylates a transcriptional repressor to control bacterial immune pathway expression

Lydia Chambers, Phoolwanti Rani, Ryan Min, Elizabeth Villa, and Kevin Corbett

Corresponding author(s): Kevin Corbett (kcorbett@ucsd.edu)

Review Timeline:

Submission Date:	31st Jan 26
Editorial Decision:	9th Mar 26
Revision Received:	29th Apr 26
Editorial Decision:	22nd May 26
Revision Received:	23rd May 26
Accepted:	27th May 26

Editor: Hartmut Vodermaier

Transaction Report:

(Note: With the exception of the correction of typographical or spelling errors that could be a source of ambiguity, letters and reports are not edited. Depending on transfer agreements, referee reports obtained elsewhere may or may not be included in this compilation. Referee reports are anonymous unless the Referee chooses to sign their reports.)

Dr. Kevin D Corbett
University of California at San Diego (UCSD)
Cellular and Molecular Medicine
9500 Gilman Drive
La Jolla, CA 92093

9th Mar 2026

Re: EMBOJ-2026-123749
A DNA damage-activated kinase controls bacterial immune pathway expression

Dear Kevin,

Thank you for submitting your study on a new DNA damage-activated transcriptional system in control of bacterial immunity operons for our consideration. We sent it to three expert referees, who have now provided the below-copied reports. Since all referees acknowledge the interest and importance of the work as well as its overall quality, we would be interested in pursuing a revised version further for EMBO Journal publication. Nevertheless, each referee brings up a few substantive concerns, whose clarification should greatly strengthen the conclusiveness and overall significance of the work. I appreciate that not all of them may be equally important within the scope of the current submission, but would nevertheless encourage you to contact me with a tentative point-by-point response and revision plan early on, so that we could discuss the feasibility of the proposed additions and better define the key revision requirements here. We would also be open to extending the revision deadline if that should be helpful - as always, our scooping protection would remain valid throughout the full revision period.

Detailed information on preparing, formatting and uploading a revised manuscript can be found below and in our Guide to Authors. Thank you again for the opportunity to consider this work for The EMBO Journal, and I look forward to hearing from you in due time.

With kind regards,

Hartmut

Hartmut Vodermaier, PhD
Senior Editor, The EMBO Journal
h.vodermaier@embojournal.org

*** PLEASE NOTE: All revised manuscript are subject to initial checks for completeness and adherence to our formatting guidelines. Revisions may be returned to the authors and delayed in their editorial re-evaluation if they fail to comply to the following requirements. As a first step please read our guidelines for revised submissions:
https://link.springer.com/journal/44318/submission-guidelines#cms-Revised-submissions

1) Every manuscript requires a Data Availability section (even if only stating that no deposited datasets are included). Primary datasets or computer code produced in the current study have to be deposited in appropriate public repositories prior to resubmission, and reviewer access details provided in case that public access is not yet allowed.

2) Each figure legend must specify
- size of the scale bars that are mandatory for all micrograph panels
- the statistical test used to generate error bars and P-values
- the type error bars (e.g., S.E.M., S.D.)
- the number (n) and nature (biological or technical replicate) of independent experiments underlying each data point
- Figures may not include error bars for experiments with n<3; scatter plots showing individual data points should be used instead.

3) Revised manuscript text (including main tables, and figure legends for main and EV figures) has to be submitted as editable text file (e.g., .docx format). We encourage highlighting of changes (e.g., via text color) for the referees' reference.

4) Each main and each Expanded View (EV) figure should be uploaded as individual production-quality files (preferably in .eps, .tif, .jpg formats). For suggestions on figure preparation/layout, please refer to our Figure Preparation Guidelines:
https://media.springernature.com/original/springer-cms/rest/v1/content/27825798/data/v1

5) Point-by-point response letters should include the original referee comments in full together with your detailed responses to them (and to specific editor requests if applicable), and also be uploaded as editable (e.g., .docx) text files.

6) Please complete our Author Checklist, and make sure that information entered into the checklist is also reflected in the manuscript; the checklist will be available to readers as part of the Review Process File.

7) All authors listed as (co-)corresponding need to deposit, in their respective author profiles in our submission system, a unique ORCiD identifier linked to their name. Please see our Guide to Authors for detailed instructions.

8) Please note that supplementary information at EMBO Press has been superseded by the 'Expanded View' for inclusion of additional figures, tables, movies or datasets; with up to five EV Figures being typeset and directly accessible in the HTML version of the article.

9) To facilitate reproducibilty and cross-laboratory adoption of methodologies, please structure the Materials & Methods section as outlined in our guide to authors, including a completed Reagents and Tools Table.

10) Digital image enhancement is acceptable practice, as long as it accurately represents the original data and conforms to community standards. If a figure has been subjected to significant electronic manipulation, this must be clearly noted in the figure legend and/or the 'Materials and Methods' section. The editors reserve the right to request original versions of figures and the original images that were used to assemble the figure. Finally, we generally encourage uploading of numerical as well as gel/blot image source data.

At EMBO Press we ask authors to provide source data for the main manuscript figures. You will receive a separate email with instructions for providing source data with your revised manuscript, including how to upload and organize the files.

In the interest of ensuring the conceptual advance provided by the work, we recommend submitting a revision within 3 months (7th Jun 2026). Please discuss the revision progress ahead of this time with the editor if you require more time to complete the revisions. Use the link below to submit your revision:

Link Not Available

Referee #1:

The manuscript by Chambers et al identifies a widespread two-gene module that is associated with (for the majority) phage defence systems. The authors distinguish this CapKS as a third transcriptional regulator after BrxR/CapW and CapHP. CapS is a transcriptional repressor. Phosphorylation of CapS by CapK shifts CapS to a monomer that cannot repress. CapK is itself activated by ssDNA.

This paradigm is then further demonstrated in a VapKSC system, newly discovered and named, wherein VapS binds and inhibits VapC until p'lation of VapS by VapK again likely due to ssDNA detection. VapKSC did provide moderate phage defence.

Overall this is an exciting new addition to the field and certainly merits a broad journal such as EMBO J. On the whole, the experiments are thorough and convincing. The data are well presented and the article was a pleasure to read. Well done to the authors!

Major comment:

Bottom of page 7 - you suggest narrow palindromes might be bound by two dimers. This could easily be tested by EMSA, likely observing a "double-shift" in comparison to a wide palindrome? I would say this isn't a firm requirement, but it would certianly help to flesh out your model.

Top of page 8 - your AFold model of CapK-CapS shows monomeric CapS. All your data, including 5E, point to interaction with CapK and dimeric CapS, so this AFold model with monomeric CapS perhaps is not a good representation for your model. Please generate a CapK-CapS dimer model and discuss Ser54 accessibility.

Second paragraph page 8 - Please add a line explaining what is happening with the N145A mutant. If N154A cannot bind DNA then surely even without MMC induction the GFP should be fully de-repressed? Also please comment on the CapK deltaHTH, as the data are shown but I do not think they are mentioned.

Minor comments:

(please put line numbers in!)

Top of page 5 - what vector for reporter, what host strain?
Top of page 5 - typo log-phage should be log phase
Fig 2A - maybe label the capS gene as FLAG-capS?
Fig 2B - comes a lot later out of order. I know this was done to keep the CHIP-seq data together, but I think just pull out 2B and add it as 3G to stop the cognitive disruption in the flow.
Fig 2D - what about probes with just the TTAA randomised, to show it is these sequence of importance?
Table S5 - the Rama allowed/outlier row seems odd, with every allowed value at 100% - do you mean the remainder from favoured adds up to 100%? Please put the allowed value and also a row stating rama outliers (hopefully zero)
Figure 3D - you showed impact on wide binding, what about on narrow sequence binding?
Top of page 10 - say "proposed" RNA target. Though likely (as you point out in Discussion), it is not confirmed.

Referee #2:

Numerous studies have demonstrated that bacteria encode a wide variety of anti-phage defense systems, which can be classified into early and late acting defense mechanisms. Many late-stage defense systems function through abortive infection mechanisms resulting in cell death of individual cells. Therefore, a fine-tuned and tightly coordinated regulation of these systems is essential to ensure effective protection while preventing intoxication of the cell in absence of infection. In the present study, the authors provide insights into a new family of DNA damage activated transcriptional regulators, directly controlling the gene expression of downstream immune pathways. To characterize the transcriptional regulators, they combined a broad variety of methods, including bioinformatic predictions, crystal structure analysis, biochemical interaction/ binding assays, and GFP reporter assays. The authors propose a regulatory mechanism in which the homodimeric transcriptional regulator CapS repress expression of downstream immune pathway genes by binding to the cognate promoter region. After binding ssDNA as the result of DNA-damage, the kinase CapK phosphorylates CapS which disrupts its homodimer structure and results in a loss of binding and thus, the induction of downstream gene expression. In addition, the authors identified CapK/CapS-like proteins that form together with the nuclease VapC, a toxin-antitoxin system which is regulated in a similar manner by DNA damage.

The authors provide detailed insights into the mechanism of the newly identified regulatory regulators CapK and CapS. Especially the combination of crystallographic analysis, protein modeling and experimental validation is well integrated and highly convincing. Overall, this is a very good study, and we suggest below experiments that could help strengthen it,but are not required for publication.

• While the study presents robust and comprehensive data on the general mechanism, the link to phage defense is not experimentally shown. It would be interesting to show how antiphage phenotype is impacted by this transcriptional regulation.
• In several experiments, the authors were able to convincingly show that phosphorylation of CapS disrupts its homodimer form, resulting in no longer binding to the target promoter region and thus, to increased expression of the downstream operons. In contrast, the ChIP seq based binding assay clearly demonstrated that CapS still binds to the target DNA even after treatment with MMC (Fig 2A). As this clearly contradicts expectations and the other results of the study, the authors argue that CapS might not be completely removed from the DNA and/or that CapS-DNA binding was already restored one hour after treatment. However, this explanation is not consistent with other findings of the study, as the authors were able to show that even one hour after treatment with MMC, the majority of CapS is still in its phosphorylated state (Fig. 4D). Since the effect of phosphorylated vs not phosphorylated CapS is one of the major findings of this study, the authors should address/discuss this discrepancy further.
• The authors mentioned that the S54E mutation of CapS results in phosphorylation of the protein. As this mutant is never used in one of the functional assays, it would be interesting to include this mutation as a positive control in the GFP-expression and CapS mobility shift assay (Fig 4D). In addition, the authors used a ΔHTH CapK mutant in which the N-terminal HTH domain was truncated (Fig 4D). If this mutant is an important control for the shown experiment, it would be useful to explain why this mutant was used as a control and how the results can be interpreted.
• The authors state several times that CapS controls its own expression by binding to its promoter region (Fig 2 & 4D). However, while promoter binding indeed suggests a possible regulatory role, it does not automatically demonstrate functional autoregulation. To support this conclusion, a functional capS promoter assay could be performed. Such an assay would include cloning of the predicted capS promoter region upstream of a suitable reporter gene (e.g., gfp) and measuring the reporter activity in the wildtype and a capS mutant. A significant change in the promoter activity would further strengthen the hypothesis of autoregulation. Since the potential autoregulation of CapS is not the main objective of the paper, it is up to the authors if they want to further address this question.

• It would be important to provide the source data, notably for all the gels

Minor comments:
• Fig 1A: labelling of the figure legend is wrong, as bacterial immune pathways are displayed in grey and not pink
• Numbers of the associated downstream operons of CapK+CapS displayed in Fig 1B do not match with written numbers in the text
• Fig 2A: one of the no treatment controls are missing
• Fig 2B: the authors clearly show that mimicking phosphorylation of CapS by S54 to glutamate prevents binding to the target DNA. However, the authors would improve the structure and the comprehension of the paper if they moved this result to Figure 3, as that is where the corresponding mutation and the effect is discussed
• Fig 6E and F: only two replicates are shown for induction assay in Fig 6E (induced sample) and 6F (uninduced sample). Given that both experiments are presented in the main text and that the VapS/VapC induction assay represents a relatively moderate experimental effort, the authors are encouraged to repeat the missing replicates to provide more robust and

Referee #3:

In this study, Chambers et al. investigate how bacteria regulate the expression of immune and stress-response pathways that can be deleterious to the host cell if inappropriately activated. Specifically, the authors address the important question of how DNA damage is sensed and transduced into transcriptional activation of adjacent immune operons, many of which encode abortive infection or toxin-like functions.
The authors identify a conserved regulatory module composed of a kinase (CapK) and a DNA-binding repressor (CapS) that is positioned upstream of diverse immune pathways. They demonstrate that CapS functions as a transcriptional repressor whose activity is relieved upon phosphorylation by CapK. Importantly, CapK kinase activity is directly stimulated by single-stranded DNA (ssDNA) generated during DNA repair, providing a mechanistic link between genome damage and immune pathway activation. The study further shows that this regulatory logic is modular and has been repurposed in a toxin-antitoxin context, where CapK/CapS-like proteins control activation of a VapC-family nuclease in response to DNA damage.
While the study presents an interesting regulatory framework, several key points should be addressed before publication. In particular, the mechanistic connection between the in vivo DNA damage response and the in vitro ssDNA-dependent kinase activation remains insufficiently supported.

Major points
The authors showed that both the capKS operon promoter and downstream immune operon promoters are activated by treatment with DNA-damaging antibiotics, and linked this activation to CapK kinase activity in the presence of ssDNA and phosphorylation of the CapS repressor. However, the connection between the bacterial DNA damage response and the proposed ssDNA-mediated activation mechanism is not yet convincing.
a) Can the authors directly demonstrate an increase in ssDNA levels following mitomycin C treatment in vivo?
b) Can the authors demonstrate co-purification or physical association of CapK with ssDNA from mitomycin-treated cells? If so, what are the length and sequence characteristics of the associated ssDNA?
c) What is the minimal and maximal ssDNA length required for CapK activation? Is there any sequence specificity? Could ssRNA also activate CapK? Does DNA methylation influence kinase activation?

The authors repeatedly suggest that capKS expression can be activated not only by DNA damaging antibiotics but also by phage infection; however, this claim is not directly tested experimentally.
a) The authors could use their GFP reporter system to measure capKS expression and activity following infection with phages that induce host DNA degradation, such as T-even phages.
b) The authors should test whether capKS operons located upstream of known anti-phage systems (e.g., CBASS, Bil, or Thoeris) confer higher expression of these systems and stronger protection against phages that damage host DNA. Otherwise, the claims regarding a general role of CapK and CapS in immune system activation should be significantly toned down.

Minor points
1) The authors identify CapS DNA binding sites (TTAAA), but how conservative it is? A comparative analysis of CapS-binding motifs across multiple capKS operons, presented as a sequence logo, would be informative. This analysis could also help estimate the impact of point or double mutations on CapS binding sites.
2) The authors demonstrate VapKSC-mediated defense against the phage Bas42. Have other closely related phages (e.g., Bas35-Bas45) been tested? If defense is observed only against Bas42, comparative genomics could help explain this specificity. In addition, selecting phage mutants that overcome VapKSC defense could identify the phage gene(s) responsible for pathway activation, thereby strengthening the proposed link between DNA damage (ssDNA generation?) and VapK activation.

Responses to Referees

We thank all three Referees for their enthusiasm for our findings, and for their insightful questions that have stimulated further experiments to strengthen the revised manuscript. We have made several substantive additions to the manuscript as a result of reviewer feedback. These additions include:

- In response to a reviewer question, we have added electrophoretic mobility-shift assays (EMSAs) to detect the binding of multiple CapS dimers on the “narrow” and “wide” palindromic regions. These data are shown in **Figure 2F** of the revised manuscript. On both DNA substrates, the EMSAs clearly show an initial mobility shift consistent with high-affinity binding of one CapS dimer, followed by a super-shift consistent with the binding of additional CapS dimers. The shift-supershift pattern is the same for both narrow and wide palindromes. We discuss the implications of this observation in the revised text.
- We now show the effects of the CapS N145A mutant on binding to both narrow and wide palindromes. As expected, binding to both DNAs is strongly compromised in the mutant. These data are shown in **Figure EV3** of the revised manuscript.
- We have added CapS-DNA binding experiments where we mutate one half-site of the narrow and wide palindromes. Binding is compromised in both cases. These data are shown in **Figure 2D-E** of the revised manuscript. In addition, closer inspection of the CapS-DNA binding curves revealed cooperativity for narrow-palindrome binding. The implications of this finding - specifically how it supports our model of the narrow palindrome recruiting two CapS interface-1 dimers - are discussed in the text.
- We have added an experiment to determine the effects on GFP expression (in our reporter system) of the CapS S54E mutation, which is expected to mimic phosphorylation. As expected, this mutation results in high expression of both GFP and FLAG-CapS in our reporter assay. These data are shown in **Figure 3F** of the revised manuscript.
- Finally, we have added a set of experiments to test CapKS dependence on two DNA repair pathways: homologous recombination (HR) and nucleotide excision repair (NER). We obtained KEIO single-gene knockout *E. coli* strains missing genes in both pathways, and tested two DNA damaging-agents, mitomycin C (MMC) and levofloxacin, using our GFP expression reporter. MMC generates inter-strand crosslinks that are repaired by NER; in keeping with this, we find that strains missing either UvrA or UvrB (early NER proteins) fail to activate CapKS upon addition of MMC. Lexofloxacin is a type II topoisomerase inhibitor, and as such indirectly generates a range of DNA damage including DNA breaks due to topological issues and replication fork breakdown. No single-gene deletion in either NER or HR eliminated CapKS activation in response to levofloxacin. Overall, these data are consistent with our in vitro results showing that CapK is activated by single-stranded DNA - which in cells is a byproduct of multiple DNA repair pathways. These data are shown in **Figure 1F-G** of the revised manuscript.

In the section below, all Referee comments are shown in black text, and our responses are indented in blue text. In the attached revised manuscript, all new and edited text is shown in red text.

Referee #1:

The manuscript by Chambers et al identifies a widespread two-gene module that is associated with (for the majority) phage defence systems. The authors distinguish this CapKS as a third transcriptional regulator after BrxR/CapW and CapHP. CapS is a transcriptional repressor. Phosphorylation of CapS by CapK shifts CapS to a monomer that cannot repress. CapK is itself activated by ssDNA.

This paradigm is then further demonstrated in a VapKSC system, newly discovered and named, wherein VapS binds and inhibits VapC until p'lation of VapS by VapK again likely due to ssDNA detection. VapKSC did provide moderate phage defence.

Overall this is an exciting new addition to the field and certainly merits a broad journal such as EMBO J. On the whole, the experiments are thorough and convincing. The data are well presented and the article was a pleasure to read. Well done to the authors!

Major comment:

Bottom of page 7 - you suggest narrow palindromes might be bound by two dimers. This could easily be tested by EMSA, likely observing a "double-shift" in comparison to a wide palindrome? I would say this isn't a firm requirement, but it would [certainly] help to flesh out your model.

> This is a good suggestion. We designed 36-bp fluorescently labeled probes for EMSA assays with centrally-positioned narrow or wide palindrome sequences. Representative EMSAs are now shown in **Figure 2F**. In both cases, we observe a shifted band appearing at sub-micromolar protein concentration, then a super-shift as protein concentration is increased to a maximum of 10 micromolar. The shift/supershift pattern, including the position of the initial shifted band on the gel, is nearly identical between the narrow and wide palindromes. This is not what we would expect to observe if initial CapS binding to the narrow palindrome involves two “interface 1” dimers. It is possible that the narrow palindrome sequence can tightly bind an “interface 1” dimer with one protomer binding one of the two half-sites, and the other protomer binding a less well-conserved flanking sequence.

Top of page 8 - your AFold model of CapK-CapS shows monomeric CapS. All your data, including 5E, point to interaction with CapK and dimeric CapS, so this AFold model with monomeric CapS perhaps is not a good representation for your model. Please generate a CapK-CapS dimer model and discuss Ser54 accessibility.

> While CapS is a homodimer in solution, the predicted CapK-CapS structure is incompatible with a CapS dimer assembled through either dimer interface 1 (completely occluded by CapK binding) or dimer interface 2 (partially occluded by CapK binding). We have added new panels to **Appendix Figure S1** illustrating this overlap, and noted this in the Results section.
>
> While CapS is predominantly dimeric in solution (assembled through dimer interface 1), there is likely a minor population of monomeric CapS that CapK can bind and phosphorylate, preventing re-dimerization. Our copurifications of *Vibrio* CapK and CapS

suggest a 1:1 stoichiometry (by band intensity; see **Appendix Figure S1**), but we are unable to measure this stoichiometry because the complex is unstable, and dissociates on a gel filtration column.

Second paragraph page 8 - Please add a line explaining what is happening with the N145A mutant. If N154A cannot bind DNA then surely even without MMC induction the GFP should be fully de-repressed? Also please comment on the CapK deltaHTH, as the data are shown but I do not think they are mentioned.

We performed additional DNA binding assays on the CapS N145A mutant with both the narrow and wide palindrome DNAs. After optimizing experimental conditions, we find that for both DNAs, the N145A mutant reduces DNA binding by 3-5 fold in vitro (see **Figure EV3**). These data are in line with our GFP expression reporter results showing that mutation of CapS N145 to alanine does not completely de-repress the expression of GFP.

We have also added a short discussion of the CapK delta-HTH mutant.

Minor comments:

(please put line numbers in!)

Apologies; we have added line numbers to the revised manuscript.

Top of page 5 - what vector for reporter, what host strain?

This information is described in the **Methods**. The *E. coli* strain used was MG1655, and the vector for reporter assays is an IPTG-inducible vector, pTRC99a.

Top of page 5 - typo log-phage should be log phase

Fixed.

Fig 2A - maybe label the capS gene as FLAG-capS?

We have updated this figure as suggested.

Fig 2B - comes a lot later out of order. I know this was done to keep the CHIP-seq data together, but I think just pull out 2B and add it as 3G to stop the cognitive disruption in the flow.

We have moved **Figure 2B** to **Figure 3** as suggested (now **Figure 3E**).

Fig 2D - what about probes with just the TTAA randomised, to show it is these sequence of importance?

This is a good suggestion. We generated half-site mutants for both the narrow and wide palindromes, and show that in both cases, binding is compromised (**Figure 2D-E**).

Table S5 - the Rama allowed/outlier row seems odd, with every allowed value at 100% - do you mean the remainder from favoured adds up to 100%? Please put the allowed value and also a row stating rama outliers (hopefully zero)

We apologize for the confusion. We have now added separate lines for favored, allowed, and disallowed Ramachandran percentages. For the allowed residues, we include only the residues that are not in favored regions. For example, where we previously reported

"98.32/100.00" for favored/allowed, we now report "98.32/1.68/0.00" for favored/allowed/disallowed.

Figure 3D - you showed impact [of the CapS N145A mutant] on wide binding, what about on narrow sequence binding?

This is a good suggestion. We now show the effect of the CapS N145A mutant on binding both the narrow and wide palindromes. As we note above, these optimized assays show that the N145A mutation causes a ~3-5 fold reduction in the affinity of DNA binding (**Figure EV3**).

Top of page 10 - say "proposed" RNA target. Though likely (as you point out in Discussion), it is not confirmed.

Added.

Referee #2:

Numerous studies have demonstrated that bacteria encode a wide variety of anti-phage defense systems, which can be classified into early and late acting defense mechanisms. Many late-stage defense systems function through abortive infection mechanisms resulting in cell death of individual cells. Therefore, a fine-tuned and tightly coordinated regulation of these systems is essential to ensure effective protection while preventing intoxication of the cell in absence of infection. In the present study, the authors provide insights into a new family of DNA damage activated transcriptional regulators, directly controlling the gene expression of downstream immune pathways. To characterize the transcriptional regulators, they combined a broad variety of methods, including bioinformatic predictions, crystal structure analysis, biochemical interaction/ binding assays, and GFP reporter assays. The authors propose a regulatory mechanism in which the homodimeric transcriptional regulator CapS repress expression of downstream immune pathway genes by binding to the cognate promoter region. After binding ssDNA as the result of DNA-damage, the kinase CapK phosphorylates CapS which disrupts its homodimer structure and results in a loss of binding and thus, the induction of downstream gene expression. In addition, the authors identified CapK/CapS-like proteins that form together with the nuclease VapC, a toxin-antitoxin system which is regulated in a similar manner by DNA damage.

The authors provide detailed insights into the mechanism of the newly identified regulatory regulators CapK and CapS. Especially the combination of crystallographic analysis, protein modeling and experimental validation is well integrated and highly convincing. Overall, this is a very good study, and we suggest below experiments that could help strengthen it,but are not required for publication.

• While the study presents robust and comprehensive data on the general mechanism, the link to phage defense is not experimentally shown. It would be interesting to show how antiphage phenotype is impacted by this transcriptional regulation.

This is certainly an important question, but unfortunately we are currently unable to test it. We have not yet identified a CapKS-controlled immune operon (CBASS, Bub, or other) that shows robust defense against bacteriophage infection in the lab.

In our prior studies of CBASS systems controlled by either CapHP (DOI: 10.15252/embj.2022111540) or CapW (DOI: 10.1093/nar/gkac343), the native operons controlled by these regulators actually show significantly weaker protection than when the same operon is constitutively expressed from a plasmid, at least in plaque assays. This weaker protection is likely because of the delay in high-level production of the immune proteins after infection, being dependent on phage-induced DNA damage.

We hypothesize that in the native environment, these expression regulators strike a compromise between high baseline immune-operon expression (which mediates strong protection against phage infection, but could lead to aberrant activation and cell death in uninfected cells) and low baseline expression (which minimizes risk of aberrant activation, but provides weaker phage protection), leading to a long-term survival benefit that is difficult to access with standard assays. We are working to test this idea, but this is a long-term effort and is out of scope for the current manuscript.

• In several experiments, the authors were able to convincingly show that phosphorylation of CapS disrupts its homodimer form, resulting in no longer binding to the target promoter region and thus, to increased expression of the downstream operons. In contrast, the ChIP seq based binding assay clearly demonstrated that CapS still binds to the target DNA even after treatment with MMC (Fig 2A). As this clearly contradicts expectations and the other results of the study, the authors argue that CapS might not be completely removed from the DNA and/or that CapS-DNA binding was already restored one hour after treatment. However, this explanation is not consistent with other findings of the study, as the authors were able to show that even one hour after treatment with MMC, the majority of CapS is still in its phosphorylated state (Fig. 4D). Since the effect of phosphorylated vs not phosphorylated CapS is one of the major findings of this study, the authors should address/discuss this discrepancy further.

The Referee is correct; we now discuss this result specifically and include our reasoning behind the observation. Since ChIP-Seq is not a quantitative method, it's difficult to judge whether CapS-DNA binding is reduced in MMC treatment compared to controls. Another complication is that in MMC-treated cells, the overall amount of CapS increases significantly (compare the total amount of FLAG-CapS in lanes 1 and 2 in **Figure 4D**). Therefore, even if most CapS is phosphorylated in these cells, there may still be some unphosphorylated CapS that can bind the promoter regions.

Notably, the CapS S54E mutant (mimicking phosphorylation) does not show ChIP-Seq peaks on these promoters (**Figure 3E**), and shows de-repression of GFP (and CapS) expression (**Figure 3F**).

• The authors mentioned that the S54E mutation of CapS results in phosphorylation of the protein. As this mutant is never used in one of the functional assays, it would be interesting to include this mutation as a positive control in the GFP-expression and CapS mobility shift assay

(Fig 4D). In addition, the authors used a ∆HTH CapK mutant in which the N-terminal HTH domain was truncated (Fig 4D). If this mutant is an important control for the shown experiment, it would be useful to explain why this mutant was used as a control and how the results can be interpreted.

Clarification: The CapS S54E mutant *mimics* phosphorylation; it does not *result in* phosphorylation. As suggested by the reviewer, we have added a GFP expression reporter assay with the CapS S54E mutant (**Figure 3F**). As expected, we observe constitutively high GFP expression in both the presence and absence of DNA damage.

As noted in the response to Referee #1, we have also added a short discussion of the CapK delta-HTH mutant results to the revised manuscript.

• The authors state several times that CapS controls its own expression by binding to its promoter region (Fig 2 & 4D). However, while promoter binding indeed suggests a possible regulatory role, it does not automatically demonstrate functional autoregulation. To support this conclusion, a functional capS promoter assay could be performed. Such an assay would include cloning of the predicted capS promoter region upstream of a suitable reporter gene (e.g., gfp) and measuring the reporter activity in the wildtype and a capS mutant. A significant change in the promoter activity would further strengthen the hypothesis of autoregulation. Since the potential autoregulation of CapS is not the main objective of the paper, it is up to the authors if they want to further address this question.

While we do not have the specific reporter the Referee describes, we do have a way to measure CapS expression in our reporter plasmids, since CapS is FLAG tagged in one of our GFP expression reporter constructs. Comparing the amount of CapS in the absence versus presence of mitomycin C (in both **Figures 3F** and **4D**), it is evident that CapS expression is significantly increased upon MMC treatment.

• It would be important to provide the source data, notably for all the gels

In the revised submission, we provide all source data including uncropped images for all gels, graph data in Excel format, and bedgraph files from ChIP-Seq experiments, as required by *The EMBO Journal*.

Minor comments:

• Fig 1A: labelling of the figure legend is wrong, as bacterial immune pathways are displayed in grey and not pink

Thanks; we have removed the reference to immune pathways as pink in the legend to **Figure 1A**.

• Numbers of the associated downstream operons of CapK+CapS displayed in Fig 1B do not match with written numbers in the text

We apologize for the inconsistency, which we have now fixed.

• Fig 2A: one of the no treatment controls are missing

One of the three no-treatment control samples failed during sample preparation, and is therefore not shown.

• Fig 2B: the authors clearly show that mimicking phosphorylation of CapS by S54 to glutamate prevents binding to the target DNA. However, the authors would improve the structure and the comprehension of the paper if they moved this result to Figure 3, as that is where the corresponding mutation and the effect is discussed

We have moved **Figure 2B** to **Figure 3** as suggested (now **Figure 3E**).

• Fig 6E and F: only two replicates are shown for induction assay in Fig 6E (induced sample) and 6F (uninduced sample). Given that both experiments are presented in the main text and that the VapS/VapC induction assay represents a relatively moderate experimental effort, the authors are encouraged to repeat the missing replicates to provide more robust and

We performed these assays again and now show graphs of triplicate data for these panels.

Referee #3:

In this study, Chambers et al. investigate how bacteria regulate the expression of immune and stress-response pathways that can be deleterious to the host cell if inappropriately activated. Specifically, the authors address the important question of how DNA damage is sensed and transduced into transcriptional activation of adjacent immune operons, many of which encode abortive infection or toxin-like functions.

The authors identify a conserved regulatory module composed of a kinase (CapK) and a DNA-binding repressor (CapS) that is positioned upstream of diverse immune pathways. They demonstrate that CapS functions as a transcriptional repressor whose activity is relieved upon phosphorylation by CapK. Importantly, CapK kinase activity is directly stimulated by single-stranded DNA (ssDNA) generated during DNA repair, providing a mechanistic link between genome damage and immune pathway activation. The study further shows that this regulatory logic is modular and has been repurposed in a toxin-antitoxin context, where CapK/CapS-like proteins control activation of a VapC-family nuclease in response to DNA damage.

While the study presents an interesting regulatory framework, several key points should be addressed before publication. In particular, the mechanistic connection between the in vivo DNA damage response and the in vitro ssDNA-dependent kinase activation remains insufficiently supported.

Major points

The authors showed that both the capKS operon promoter and downstream immune operon promoters are activated by treatment with DNA-damaging antibiotics, and linked this activation to CapK kinase activity in the presence of ssDNA and phosphorylation of the CapS repressor. However, the connection between the bacterial DNA damage response and the proposed ssDNA-mediated activation mechanism is not yet convincing.

a) Can the authors directly demonstrate an increase in ssDNA levels following mitomycin C treatment in vivo?

We are not aware of a readily accessible method to measure the amount of ssDNA in live cells. Any lysis method produces large amounts of sheared/fragmented genomic DNA, making detection after cell lysis essentially impossible.

As a partial alternative, we can closely examine the effects of deleting selected genes from two DNA repair pathways - homologous recombination (HR) and nucleotide excision repair (NER) - and the dependence of CapKS activation on each of these genes. This data, for both mitomycin C and levofloxacin, is shown in **Figure 1F-G**. The results support the connection between CapKS activation and ssDNA production in three ways:

1. In a *recA* deletion mutant, GFP expression is moderate in the absence of DNA damage. Since RecA is a recombinase that binds resected single-stranded DNA and mediates strand exchange in HR, this intermediate is expected to build up in a *recA* deletion.
2. Both *uvrA* and *uvrB* deletions fail to activate CapKS after incubation with mitomycin C. Mitomycin C generates interstrand crosslinks, which must be repaired by NER. UvrA and UvrB are responsible for initial recognition of the cross-linked DNA strands. The failure of *uvrA* and *uvrB* deletions to activate CapKS is consistent with these cells' expected inability to initially process MMC-damaged DNA.
3. As a further point, UvrA and UvrB are not required for CapKS activation upon levofloxacin treatment. This is consistent with levofloxacin (a DNA topoisomerase inhibitor) generating multiple types of damage, the processing of which by multiple pathways (including but not limited to HR) gives rise to ssDNA.

b) Can the authors demonstrate co-purification or physical association of CapK with ssDNA from mitomycin-treated cells? If so, what are the length and sequence characteristics of the associated ssDNA?

When lysing bacterial cells, the genomic DNA becomes fragmented to the point where it is impossible to judge whether any co-purifying DNA was associated with a protein prior to cell lysis. Therefore, we cannot directly perform the experiment described by the Referee.

c) What is the minimal and maximal ssDNA length required for CapK activation? Is there any sequence specificity? Could ssRNA also activate CapK? Does DNA methylation influence kinase activation?

Our prior work with the CapHP system showed that CapP is activated by T-rich ssDNA (DOI: 10.15252/embj.2022111540). In our initial testing of different DNAs for CapK activation, we found that a 22-base DNA containing only T and C bases was most effective at activating CapK (now included in **Figure EV4**). Notably, this assay also included 10-base poly-T and poly-C DNAs, which did not activate CapK. We have not tested ssDNA longer than 22 bases. These data support a minimum length of ~20

bases, and a preference for T/C containing DNA, for CapK activation. These findings are discussed in the revised text.

The CapK activation assay shown in **Figure EV4** includes one 22-base ssRNA, which did not activate CapK activation. Given the abundance of ssRNA in cells at all times, we anticipate that CapK has evolved to *not* be activated by ssRNA.

With respect to the question about DNA methylation, we have not tested this idea. We note, however, that our GFP reporter assays are all done in host cells with addition of a DNA damaging agent, so there is no DNA methylation involved. There is the possibility that DNA methylation could disrupt ssDNA recognition by CapK and thereby escape CapK activation; however our model is that CapK recognizes byproducts of host genome damage, not phage DNA, making the involvement of DNA methylation unlikely.

The authors repeatedly suggest that capKS expression can be activated not only by DNA damaging antibiotics but also by phage infection; however, this claim is not directly tested experimentally.

a) The authors could use their GFP reporter system to measure capKS expression and activity following infection with phages that induce host DNA degradation, such as T-even phages.

We have tested our GFP expression reporters with phages (lambda and Bas42, a T4-like phage), and we have not observed consistent GFP expression after infection. This is counter to expectations, as the reviewer notes.

While we do not have a complete explanation for the failure of phage infection to activate CapKS in our system, we hypothesize that this may be due to the high copy number of the plasmid. We are using plasmid pTRC99a which has a pBR322 origin of replication, giving an expected 15-20 copies of this plasmid per cell. Since our GFP expression reporters include the native promoter for *capS* (between the *capK* and *capS* genes), the level of CapS protein in these cells is expected to be up to 15-20 times that of the case in which *capKS* is present in the genome. This higher level of CapS protein could result in stronger repression of the downstream promoter, and consequently lower responsiveness to perturbations. The very high level of DNA damaging agents we are using in our experiments is enough to activate CapKS, but phage infection may not be.

This is an intriguing hypothesis and may also explain some of our prior data on CapW and CapHP. We plan to pursue this question in the future, but since it requires genomic integration of various constructs and exhaustive tests of expression and phage protection, it is out of scope for the current work.

b) The authors should test whether capKS operons located upstream of known anti-phage systems (e.g., CBASS, BiI, or Thoeris) confer higher expression of these systems and stronger protection against phages that damage host DNA. Otherwise, the claims regarding a general role of CapK and CapS in immune system activation should be significantly toned down.

As noted above, we don’t currently have the ability to perform the requested experiments, since we have not yet identified an immune operon controlled by CapKS that shows protection against phage infection in the lab.

Nonetheless, we disagree with the Referee that our claims need to be toned down. Our experiments clearly demonstrate that CapKS is an expression regulator, and that CapKS is associated with many known and proposed immune operons. We specifically avoid claiming that CapKS regulation improves a given immune operon's ability to protect against a lytic phage infection (as assayed in the lab through plaque assays) - indeed as noted above, we have observed exactly the opposite in plaque assays for CBASS systems controlled by CapHP (DOI: 10.15252/embj.2022111540) and CapW (DOI: 10.1093/nar/gkac343). As discussed in those prior works and as noted above and in our manuscript, we hypothesize that tight control over immune operon expression provides a long-term fitness advantage to host cells, even though it may reduce protection as measured by plaque assays.

Minor points

1) The authors identify CapS DNA binding sites (TTAAA), but how conservative it is? A comparative analysis of CapS-binding motifs across multiple capKS operons, presented as a sequence logo, would be informative. This analysis could also help estimate the impact of point or double mutations on CapS binding sites.

We thank the reviewer for pointing out this omission. Indeed, we performed this analysis previously to convince ourselves that the narrow and wide palindrome sequences were conserved. We downloaded the genomic sequence of 100 *capS* genes related to the gene from *E. roggenkampii* (+/- 200 bp), performed a sequence alignment, and generated sequence logos of both the narrow and wide palindrome sequences. These are now added to **Figure 2B-C**, and the original sequence FASTA files are provided as source data.

Based on our re-examining these data, we have adjusted what we call the palindromic sequence. Instead of "TTAAA" half-sites, the logos show that the conservation is actually limited to "TTAA". We have made changes to both **Figure 2** and **Figure 3**, and adjusted the text to reflect this change.

2) The authors demonstrate VapKSC-mediated defense against the phage Bas42. Have other closely related phages (e.g., Bas35-Bas45) been tested? If defense is observed only against Bas42, comparative genomics could help explain this specificity. In addition, selecting phage mutants that overcome VapKSC defense could identify the phage gene(s) responsible for pathway activation, thereby strengthening the proposed link between DNA damage (ssDNA generation?) and VapK activation.

These are good suggestions. We did test VapKSC against BASEL phages 14-69, and observed weak protection against phages Bas38, Bas40, Bas41, Bas42, and Bas47. VapKSC protection was marginally stronger against Bas42 than the others; this was therefore the phage we used for triplicate measurements with VapKSC mutants. Because the initial screen was not performed in triplicate, we chose not to include it in the manuscript.

With respect to the Referee's suggestions about phage escapers, we don't consider this a good system to isolate escapers because of the relatively weak protection afforded by

VapKSC (less than 2 logs in a plaque assay); this would make it very difficult to successfully select for escaper mutants.

Dr. Kevin D Corbett
University of California at San Diego (UCSD)
Cellular and Molecular Medicine
9500 Gilman Drive
La Jolla, CA 92093

22nd May 2026

Re: EMBOJ-2026-123749R
A DNA damage-activated kinase controls bacterial immune pathway expression

Dear Kevin,

Thank you for submitting your revised manuscript on CapK/S in bacterial immune pathways for our consideration. It has now been again seen by the three original referees (see comments below), who were overall satisfied with the revisions and raised no further objections. We shall therefore be happy to accept the manuscript for publication, following incorporation of a few remaining editorial points as follows:

- Please adjust the order of the manuscript sections, and also make sure to use the correct section headers:
Single title page with complete author information, Abstract, Introduction, Results, Discussion, Methods, Data Availability, Acknowledgements, Disclosure and Competing Interests Statement, References, Main Figure Legends, Tables, Expanded Figure Legends.

- As we are switching from a free-text author contribution statement towards a more formal statement based on Contributor Role Taxonomy (CRediT) terms, please remove the present Author Contribution section and instead specify each author's contribution(s) directly in the Author Information page of our submission system during upload of the final manuscript. See https://casrai.org/credit/ for more information.

- Please carefully go through the reference list and make sure that all references have their complete citation information - including year, journal name & volume, and page/elocator numbers - such information is currently missing for several of them.

- For preprint citations: Please change in-text reference format according to "preprint: Miller et al, 2025"; and in the reference list, name of the platform plus DOI + preprint label - e.g. "bioRxiv doi: 1234/002.dfj123 [PREPRINT]"

- In the Data Availability section, please make sure that all deposited entries become publicly released by the time of publication and all links are functional.

- In the figure legends, please define the measure of center for the error bar needs for Figure 6K.

- Please convert 'Appendix Tables' S1/2/4 into Expanded View Datasets: Each of them should be uploaded as a separate XLSX file, with a 'Legends' tab including its name/title/info. Naming and in-text referencing should be 'Dataset EV1/2/3'

- For the Appendix PDF, please preface it with a title page stating "Appendix for [manuscript title]", and include page numbers for the items listed in the ToC. Furthermore, please remove Appendix Tables S1/2/4 from the listing (see above), and renumber Appendix Tables S3 and S5 as Appendix Table S1/S2, adjusting their in-text referencing accordingly.

- Please provide suggestions for a short 'blurb' text prefacing and summing up the conceptual aspect of the study in two sentences (max. 250 characters), followed by 3-5 one-sentence 'bullet points' with brief factual statements of key results of the paper; they will form the basis of an editor-written 'Synopsis' accompanying the online version of the article. Please also upload a synopsis image, which can be used as a "visual title" for the synopsis section of your paper. The image (maybe based on a more compacted version of Figure 7?) should be ideally in JPG format, and please make sure that it remains in the modest dimensions of (exactly) 550 pixels wide and between 300 pixels high.

- Finally, I wonder whether the title could be made somewhat more explicit/specific (even if then slightly exceeding the stipulated 100-character limit), e.g. by mentioning the proteins, anti-phage, anything that would allow better associating the title with the specific findings? And in the abstract, it would probably not harm to mention particular species in which key analyses were conducted here?

I am returning the manuscript to you for a final round of minor revision, solely to allow you to make these modifications and upload the revised files. Once we will have received them, we should be ready to swiftly proceed with formal acceptance and production of the manuscript.

With kind regards,

Hartmut

Hartmut Vodermaier, PhD
Senior Editor, The EMBO Journal
h.vodermaier@embojournal.org

*** PLEASE NOTE: All revised manuscript are subject to initial checks for completeness and adherence to our formatting guidelines. Revisions may be returned to the authors and delayed in their editorial re-evaluation if they fail to comply to the following requirements. As a first step please read our guidelines for revised submissions:
https://link.springer.com/journal/44318/submission-guidelines#cms-Revised-submissions

1) Every manuscript requires a Data Availability section (even if only stating that no deposited datasets are included). Primary datasets or computer code produced in the current study have to be deposited in appropriate public repositories prior to resubmission, and reviewer access details provided in case that public access is not yet allowed.

2) Each figure legend must specify
- size of the scale bars that are mandatory for all micrograph panels
- the statistical test used to generate error bars and P-values
- the type error bars (e.g., S.E.M., S.D.)
- the number (n) and nature (biological or technical replicate) of independent experiments underlying each data point
- Figures may not include error bars for experiments with n<3; scatter plots showing individual data points should be used instead.

3) Revised manuscript text (including main tables, and figure legends for main and EV figures) has to be submitted as editable text file (e.g., .docx format). We encourage highlighting of changes (e.g., via text color) for the referees' reference.

4) Each main and each Expanded View (EV) figure should be uploaded as individual production-quality files (preferably in .eps, .tif, .jpg formats). For suggestions on figure preparation/layout, please refer to our Figure Preparation Guidelines:
https://media.springernature.com/original/springer-cms/rest/v1/content/27825798/data/v1

5) Point-by-point response letters should include the original referee comments in full together with your detailed responses to them (and to specific editor requests if applicable), and also be uploaded as editable (e.g., .docx) text files.

6) Please complete our Author Checklist, and make sure that information entered into the checklist is also reflected in the manuscript; the checklist will be available to readers as part of the Review Process File.

7) All authors listed as (co-)corresponding need to deposit, in their respective author profiles in our submission system, a unique ORCiD identifier linked to their name. Please see our Guide to Authors for detailed instructions.

8) Please note that supplementary information at EMBO Press has been superseded by the 'Expanded View' for inclusion of additional figures, tables, movies or datasets; with up to five EV Figures being typeset and directly accessible in the HTML version of the article.

9) To facilitate reproducibilty and cross-laboratory adoption of methodologies, please structure the Materials & Methods section as outlined in our guide to authors, including a completed Reagents and Tools Table.

10) Digital image enhancement is acceptable practice, as long as it accurately represents the original data and conforms to community standards. If a figure has been subjected to significant electronic manipulation, this must be clearly noted in the figure legend and/or the 'Materials and Methods' section. The editors reserve the right to request original versions of figures and the original images that were used to assemble the figure. Finally, we generally encourage uploading of numerical as well as gel/blot image source data.

At EMBO Press we ask authors to provide source data for the main manuscript figures. You will receive a separate email with instructions for providing source data with your revised manuscript, including how to upload and organize the files.

In the interest of ensuring the conceptual advance provided by the work, we recommend submitting a revision within 3 months (20th Aug 2026). Please discuss the revision progress ahead of this time with the editor if you require more time to complete the revisions. Use the link below to submit your revision:

Link Not Available

Referee #1:

The revised manuscript does an excellent job of responding to earlier reviewer comments. Nothing further from me. Congratulations to the authors!

Referee #2:

The authors answered my comments adequately

Referee #3:

The authors have partially addressed my previous concerns, and I am satisfied with several of their responses.

However, a substantial part of my main questions remains unanswered, largely due to methodological limitations or other objective constraints. Therefore, it is difficult for me to fully evaluate how critical these unresolved points are for the overall conclusions of the study.

That said, if the editor and the other reviewers consider the authors' responses satisfactory, I would support publication of the manuscript in its current form.

EMBO Press Author Checklist

Corresponding Author Name: Kevin Corbett	
Journal Submitted to: The EMBO Journal	
Manuscript Number: EMBOJ-2026-123749	

USEFUL LINKS FOR COMPLETING THIS FORM
The EMBO Journal - Author Guidelines
EMBO Reports - Author Guidelines
Molecular Systems Biology - Author Guidelines
EMBO Molecular Medicine - Author Guidelines

Reporting Checklist for Life Science Articles (updated January
This checklist is adapted from Materials Design Analysis Reporting (MDAR) Checklist for Authors. MDAR establishes a minimum set of requirements in transparent reporting in the life sciences (see Statement of Task: 10.31222/osf.io/9sm4x). Please follow the journal's guidelines in preparing your
Please note that a copy of this checklist will be published alongside your article.

Abridged guidelines for figures
1. Data
The data shown in figures should satisfy the following conditions:
- the data were obtained and processed according to the field's best practice and are presented to reflect the results of the experiments in an accurate and unbiased manner.
- ideally, figure panels should include only measurements that are directly comparable to each other and obtained with the same assay.
- plots include clearly labeled error bars for independent experiments and sample sizes. Unless justified, error bars should not be shown for technical
- if n<5, the individual data points from each experiment should be plotted. Any statistical test employed should be justified.
- Source Data should be included to report the data underlying figures according to the guidelines set out in the authorship guidelines on Data

2. Captions
Each figure caption should contain the following information, for each panel where they are relevant:
- a specification of the experimental system investigated (eg cell line, species name).
- the assay(s) and method(s) used to carry out the reported observations and measurements.
- an explicit mention of the biological and chemical entity(ies) that are being measured.
- an explicit mention of the biological and chemical entity(ies) that are altered/varied/perturbed in a controlled manner.
- the exact sample size (n) for each experimental group/condition, given as a number, not a range;
- a description of the sample collection allowing the reader to understand whether the samples represent technical or biological replicates (including how many animals, litters, cultures, etc.).
- a statement of how many times the experiment shown was independently replicated in the laboratory.
- definitions of statistical methods and measures:
  - common tests, such as t-test (please specify whether paired vs. unpaired), simple $\chi 2$ tests, Wilcoxon and Mann-Whitney tests, can be unambiguously identified by name only, but more complex techniques should be described in the methods section;
  - are tests one-sided or two-sided?
  - are there adjustments for multiple comparisons?
  - exact statistical test results, e.g., P values = x but not P values < x;
  - definition of 'center values' as median or average;
  - definition of error bars as s.d. or s.e.m.

Please complete ALL of the questions below.
Select "Not Applicable" only when the requested information is not relevant for your study.

Materials

Newly Created Materials	Information included in the manuscript?	In which section is the information available? (Reagents and Tools Table, Materials and Methods, Figures, Data Availability Section)
New materials and reagents need to be available; do any restrictions apply?	Not Applicable	

Antibodies	Information included in the manuscript?	In which section is the information available? (Reagents and Tools Table, Materials and Methods, Figures, Data Availability Section)
For **antibodies** provide the following information:<br>- Commercial antibodies: RRID (if possible) or supplier name, catalogue number and or/clone number<br>- Non-commercial: RRID or citation	Yes	Reagents and Tools Table, Materials and Methods

DNA and RNA sequences	Information included in the manuscript?	In which section is the information available? (Reagents and Tools Table, Materials and Methods, Figures, Data Availability Section)
**Short novel DNA or RNA including primers, probes**: provide the sequences.	Yes	Reagents and Tools Table, Materials and Methods

Cell materials	Information included in the manuscript?	In which section is the information available? (Reagents and Tools Table, Materials and Methods, Figures, Data Availability Section)
**Cell lines:** Provide species information, strain. Provide accession number in repository **OR** supplier name, catalog number, clone number, and/**OR** RRID.	Not Applicable	
**Primary cultures:** Provide species, strain, sex of origin, genetic modification status.	Not Applicable	
Report if the cell lines were recently **authenticated** (e.g., by STR profiling) and tested for mycoplasma contamination.	Not Applicable	

Experimental animals	Information included in the manuscript?	In which section is the information available? (Reagents and Tools Table, Materials and Methods, Figures, Data Availability Section)
**Laboratory animals or Model organisms:** Provide species, strain, sex, age, genetic modification status. Provide accession number in repository **OR** supplier name, catalog number, clone number, **OR** RRID.	Not Applicable	
**Animal observed in or captured from the field:** Provide species, sex, and age where possible.	Not Applicable	
Please detail **housing and husbandry conditions**.	Not Applicable	

Plants and microbes	Information included in the manuscript?	In which section is the information available? (Reagents and Tools Table, Materials and Methods, Figures, Data Availability Section)
**Plants:** provide species and strain, ecotype and cultivar where relevant, unique accession number if available, and source (including location for collected wild specimens).	Not Applicable	
**Microbes:** provide species and strain, unique accession number if available, and source.	Yes	Reagents and Tools Table, Materials and Methods

Human research participants	Information included in the manuscript?	In which section is the information available? (Reagents and Tools Table, Materials and Methods, Figures, Data Availability Section)
If collected and within the bounds of privacy constraints report on age, sex and gender or ethnicity for all study participants.	Not Applicable	

Core facilities	Information included in the manuscript?	In which section is the information available? (Reagents and Tools Table, Materials and Methods, Figures, Data Availability Section)
If your work benefited from core facilities, was their service mentioned in the acknowledgments section?	Yes	Acknowledgements

Design

Study protocol	Information included in the manuscript?	In which section is the information available? (Reagents and Tools Table, Materials and Methods, Figures, Data Availability Section)
If study protocol has been **pre-registered, provide DOI in the manuscript**. For clinical trials, provide the trial registration number **OR** cite DOI.	Not Applicable	
Report the **clinical trial registration number** (at ClinicalTrials.gov or equivalent), where applicable.	Not Applicable	

Laboratory protocol	Information included in the manuscript?	In which section is the information available? (Reagents and Tools Table, Materials and Methods, Figures, Data Availability Section)
Provide DOI OR other citation details if **external detailed step-by-step protocols** are available.	Not Applicable	

Experimental study design and statistics	Information included in the manuscript?	In which section is the information available? (Reagents and Tools Table, Materials and Methods, Figures, Data Availability Section)
Include a statement about **sample size** estimate even if no statistical methods were used.	Not Applicable	
Were any steps taken to minimize the effects of subjective bias when allocating animals/samples to treatment (e.g. **randomization procedure**)? If yes, have they been described?	Not Applicable	
Include a statement about **blinding** even if no blinding was done.	Not Applicable	
Describe **inclusion/exclusion criteria** if samples or animals were excluded from the analysis. Were the criteria pre-established?<br><br>If sample or data points were omitted from analysis, report if this was due to attrition or intentional exclusion and provide justification.	Not Applicable	
For every figure, are **statistical tests** justified as appropriate? Do the data meet the assumptions of the tests (e.g., normal distribution)? Describe any methods used to assess it. Is there an estimate of variation within each group of data? Is the variance similar between the groups that are being statistically compared?	Yes	Figure legends, Materials and Methods

Sample definition and in-laboratory replication	Information included in the manuscript?	In which section is the information available? (Reagents and Tools Table, Materials and Methods, Figures, Data Availability Section)
In the figure legends: state number of times the experiment was **replicated** in laboratory.	Yes	Figure legends
In the figure legends: define whether data describe **technical or biological replicates**.	Yes	Figure legends

Ethics

Ethics	Information included in the manuscript?	In which section is the information available? (Reagents and Tools Table, Materials and Methods, Figures, Data Availability Section)
Studies involving **human participants**: State details of **authority granting ethics approval** (IRB or equivalent committee(s), provide reference number for approval.	Not Applicable	
Studies involving **human participants**: Include a statement confirming that **informed consent** was obtained from all subjects and that the experiments conformed to the principles set out in the WMA Declaration of Helsinki and the Department of Health and Human Services Belmont Report.	Not Applicable	
Studies involving **human participants:** For publication of **patient photos**, include a statement confirming that consent to publish was obtained.	Not Applicable	
Studies involving experimental **animals**: State details of **authority granting ethics approval** (IRB or equivalent committee(s), provide reference number for approval. Include a statement of compliance with ethical regulations.	Not Applicable	
Studies involving **specimen and field samples:** State if relevant **permits** obtained, provide details of authority approving study; if none were required, explain why.	Not Applicable	

Dual Use Research of Concern (DURC)	Information included in the manuscript?	In which section is the information available? (Reagents and Tools Table, Materials and Methods, Figures, Data Availability Section)
Could your study fall under dual use research restrictions? Please check biosecurity documents and list of **select agents and toxins** (CDC): https://www.selectagents.gov/sat/list.htm	Not Applicable	
If you used a select agent, is the security level of the lab appropriate and reported in the manuscript?	Not Applicable	
If a study is subject to dual use research of concern regulations, is the name of the **authority granting approval and reference number** for the regulatory approval provided in the manuscript?	Not Applicable	

Reporting

The MDAR framework recommends adoption of discipline-specific guidelines, established and endorsed through community initiatives. Journals have their own policy about requiring specific guidelines and recommendations to complement MDAR.

Adherence to community standards	Information included in the manuscript?	In which section is the information available? (Reagents and Tools Table, Materials and Methods, Figures, Data Availability Section)
State if relevant guidelines or checklists (e.g., **ICMJE, MIBBI, ARRIVE, PRISMA**) have been followed or provided.	Not Applicable	
For **tumor marker prognostic studies,** we recommend that you follow the **REMARK** reporting guidelines (see link list at top right). See author guidelines, under 'Reporting Guidelines'. Please confirm you have followed these guidelines.	Not Applicable	
For **phase II and III randomized controlled trials**, please refer to the **CONSORT** flow diagram (see link list at top right) and submit the CONSORT checklist (see link list at top right) with your submission. See author guidelines, under 'Reporting Guidelines'. Please confirm you have submitted this list.	Not Applicable	

Data Availability

Data availability	Information included in the manuscript?	In which section is the information available? (Reagents and Tools Table, Materials and Methods, Figures, Data Availability Section)
Have **primary datasets** been deposited according to the journal's guidelines (see 'Data Deposition' section) and the respective accession numbers provided in the Data Availability Section?	Yes	Materials and Methods, Data Availability Section
Were **human clinical and genomic datasets** deposited in a public access-controlled repository in accordance to ethical obligations to the patients and to the applicable consent agreement?	Not Applicable	
Are **computational models** that are central and integral to a study available without restrictions in a machine-readable form? Were the relevant accession numbers or links provided?	Not Applicable	
If publicly available data were reused, provide the respective **data citations in the reference list**.	Yes	Materials and Methods, References
