# Supplementary material for: A DNA damage-activated kinase phosphorylates a transcriptional repressor to control bacterial immune pathway expression
Source: EMBO J. 2026 Jun 9;45(14):5079–100. doi: 10.1038/s44318-026-00831-y (PMC13373217; doi:10.1038/s44318-026-00831-y)
Supplement: Supplementary file 13 — Source Data for Expanded View and Appendix Figures [file 44318_2026_831_MOESM13_ESM.zip › Source_Data_for_Expanded_View_and_Appendix/AppendixFigureS4/S4A-E/STARANISO-output.zip!/LICR_UCSD_09_12_1_3point4_STARANISO/Summary.html]

ZwB5bFSwXQ4Y\_\_Q0: STARANISO anisotropy & Bayesian estimation server


|  |  |  |
| --- | --- | --- |
| The STARANISO Server  *Anisotropy of the Diffraction Limit* and  *Bayesian Estimation of Structure Amplitudes*   | | |

---

  

- **WARNING: Reflections file from XDS CORRECT (built 20250714) used, the file from INTEGRATE is recommended.**

- **Your STARANISO job  ZwB5bFSwXQ4Y\_\_Q0  for  "LICR\_UCSD\_09\_12\_1\_XDS\_ASCII.HKL"  completed successfully.**  

  - **Merging statistics table for observed data extracted from the final MRFANA log file:**

    ```
                                                                                         Compl. Spher.   Multiplicity                                   Compl. Ellip. 
                                                                                         --------------  ------------                                  --------------- 
                Resolution      #uniq     #Rfac  Rmerge   Rmeas    Rpim   #Isig  I/sigI     all     ano    all    ano CC(1/2)  #CCAno CC(ano)  SigAno    all     ano
     -----------------------------------------------------------------------------------------------------------------------------------------------------------------
             74.221 -  10.997     656      4002   0.110   0.120   0.049     656  14.345  0.9719  0.9615   6.11   3.08  0.9928     624  0.1504   0.876   0.9719  0.9615
             10.997 -   8.766     657      3777   0.139   0.152   0.062     657  12.337  0.9970  0.9909   5.75   2.89  0.9911     594 -0.0161   0.929   0.9970  0.9909
              8.766 -   7.674     656      4006   0.199   0.218   0.088     656  10.337  1.0000  0.9985   6.11   3.06  0.9842     633 -0.1122   0.925   1.0000  0.9985
              7.674 -   6.987     654      3937   0.280   0.307   0.124     654   7.321  1.0000  0.9985   6.02   3.01  0.9696     624 -0.0670   0.821   1.0000  0.9985
              6.987 -   6.489     658      4104   0.363   0.396   0.158     658   5.592  1.0000  1.0000   6.24   3.12  0.9620     634  0.0230   0.759   1.0000  1.0000
              6.489 -   6.101     657      4113   0.464   0.506   0.201     657   4.604  1.0000  1.0000   6.26   3.13  0.9447     642 -0.0391   0.712   1.0000  1.0000
              6.101 -   5.802     657      4029   0.493   0.540   0.218     657   4.247  1.0000  0.9924   6.13   3.08  0.9389     621 -0.0061   0.704   1.0000  0.9924
              5.802 -   5.535     653      3498   0.527   0.585   0.248     653   3.603  0.9519  0.9257   5.38   2.75  0.9028     534 -0.0852   0.666   0.9519  0.9257
              5.535 -   5.326     662      3811   0.570   0.627   0.259     662   3.495  0.9985  0.9985   5.76   2.88  0.9201     608 -0.0621   0.670   0.9985  0.9985
              5.326 -   5.140     654      3865   0.646   0.709   0.289     654   3.184  1.0000  1.0000   5.91   2.95  0.9137     618  0.0090   0.677   1.0000  1.0000
              5.140 -   4.981     654      3901   0.626   0.687   0.279     654   3.368  1.0000  0.9985   5.96   2.99  0.9017     609 -0.0573   0.654   1.0000  0.9985
              4.981 -   4.840     658      3961   0.657   0.720   0.293     658   3.428  0.9970  0.9955   6.02   3.01  0.9014     628 -0.1188   0.661   0.9970  0.9955
              4.840 -   4.709     653      3963   0.660   0.723   0.294     653   3.355  0.9561  0.9546   6.07   3.04  0.9162     624  0.0625   0.666   0.9561  0.9546
              4.708 -   4.584     658      3970   0.711   0.780   0.318     658   3.250  0.9026  0.8999   6.03   3.02  0.9076     623 -0.0339   0.648   0.9026  0.8999
              4.584 -   4.454     653      3935   0.734   0.805   0.327     653   3.306  0.7691  0.7656   6.03   3.03  0.8967     609  0.0948   0.693   0.7691  0.7656
              4.454 -   4.308     660      3813   0.671   0.739   0.307     660   3.318  0.6298  0.6269   5.78   2.90  0.9259     606 -0.0562   0.666   0.8397  0.8359
              4.308 -   4.149     659      3853   0.770   0.847   0.350     659   2.972  0.4846  0.4809   5.85   2.94  0.9245     602 -0.0891   0.615   0.8916  0.8848
              4.149 -   3.963     656      3797   0.793   0.874   0.363     656   3.193  0.3527  0.3484   5.79   2.92  0.8610     587 -0.1284   0.652   0.8378  0.8276
              3.963 -   3.729     656      3750   0.932   1.028   0.429     656   2.878  0.2282  0.2250   5.72   2.89  0.8365     559 -0.0118   0.632   0.7990  0.7881
              3.729 -   3.402     655      3484   1.119   1.246   0.539     655   2.609  0.1195  0.1145   5.33   2.73  0.7570     478 -0.0440   0.550   0.7607  0.7294
     -----------------------------------------------------------------------------------------------------------------------------------------------------------------
     Total:  74.221 -   3.402   13126     77569   0.352   0.386   0.158   13126   5.037  0.5751  0.5709   5.91   2.97  0.9848   12057 -0.0068   0.709   0.9253  0.9185
    ```

    - **Merging statistics plots for observed data, extracted from the
      MRFANA log file** (click image to enlarge):

      |  |  |  |  |  |  |  |  |  |  |  |  |
      | --- | --- | --- | --- | --- | --- | --- | --- | --- | --- | --- | --- |
      | Spherical completeness *vs.* image no.:    Spherical completeness *vs.* mean *d*-spacing:   | Ellipsoidal completeness *vs.* image no.:    Ellipsoidal completeness *vs.* mean *d*-spacing:   | Merging *R* values *vs.* image no.:    Merging *R* values *vs.* mean *d*-spacing:   | CC_½_ *vs.* mean *d*-spacing:    CC_½_ano *vs.* mean *d*-spacing:   | Mean *I*/σ(*I*) *vs.* image no.:    Mean *I*/σ(*I*) *vs.* mean *d*-spacing:   | Mean *D*ano/σ(*D*ano) *vs.* mean *d*-spacing:    Spherical redundancy *vs.* mean *d*-spacing: | | | | | | | | | | | |

        
      - **Summary of merging statistics for observed data:**

        ```
                                                      Overall  InnerShell  OuterShell
          ---------------------------------------------------------------------------
             Low resolution limit                      74.221      74.221       3.729
             High resolution limit                      3.402      10.997       3.402

             Rmerge  (all I+ & I-)                      0.352       0.110       1.119
             Rmerge  (within I+/I-)                     0.302       0.099       0.922
             Rmeas   (all I+ & I-)                      0.386       0.120       1.246
             Rmeas   (within I+/I-)                     0.370       0.120       1.148
             Rpim    (all I+ & I-)                      0.158       0.049       0.539
             Rpim    (within I+/I-)                     0.212       0.068       0.676
             Total number of observations               77606        4008        3489
             Total number unique                        13126         656         655
             Mean(I)/sd(I)                                5.0        14.3         2.6
             Completeness (spherical)                    57.5        97.2        11.9
             Completeness (ellipsoidal)                  92.5        97.2        76.1
             Multiplicity                                 5.9         6.1         5.3
             CC(1/2)                                    0.985       0.993       0.757

             Anomalous completeness (spherical)          57.1        96.1        11.5
             Anomalous completeness (ellipsoidal)        91.9        96.1        72.9
             Anomalous multiplicity                       3.0         3.1         2.7
             CC(ano)                                   -0.007       0.150      -0.044
             |DANO|/sd(DANO)                            0.709       0.876       0.550
        ```

---

  
- **Data processing and anisotropy information extracted from the STARANISO log file:**

  ```
  STARANISO version: 3.0.17 (1-Oct-2025)   Run on: Fri, 17 Oct 2025 17:28:50 +0200.

  From CORRECT (VERSION Jan 19, 2025  BUILT=20250714)

  *** WARNING: Output from CORRECT or XSCALE detected, consider using the output from INTEGRATE instead.


  Using MTZ column labels:      IMEAN  SIGIMEAN  I(+)  SIGI(+)  I(-)  SIGI(-)  N(+)  N(-)  IHALF1  IHALF2

  Unit cell and space group:      159.082  159.082  176.198  90.00  90.00 120.00   'H 3'
  Nominal diffraction range:       74.221  3.400
  Input reflection count:           22725

  Diffraction cut-off criterion: Local <I/sd(I)> = 1.20

  Semi-axis d-spacings (Ang.) and corresponding principal axes of the ellipsoid fitted to the
  diffraction cut-off surface:

    Diffraction limit #1:  4.466   ( 1.0000,  0.0000,  0.0000)    0.894 a* - 0.447 b*
    Diffraction limit #2:  4.466   ( 0.0000,  1.0000,  0.0000)    b*
    Diffraction limit #3:  3.112   ( 0.0000,  0.0000,  1.0000)    c*

  The principal axes are given as direction cosines relative to the orthonormal basis (standard PDB
  convention), and also in terms of reciprocal unit-cell vectors.

  Worst diffraction limit after cut-off:
              5.165 at reflection    3   25    2  in direction   0.119 a* + 0.990 b* + 0.079 c*

  Best diffraction limit after cut-off:
              3.402 at reflection    8    4  -50  in direction   0.157 a* + 0.079 b* - 0.984 c*

  NOTE that because the cut-off surface is likely to be only very approximately ellipsoidal, in part
  due to variations in reflection redundancy arising from the chosen collection strategy, the
  directions of the worst and best diffraction limits may not correspond with the reciprocal axes,
  even in high-symmetry space groups (the only constraint being that the surface must have point
  symmetry at least that of the Laue class).

  Scale:                       1.35353E+02    [ = factor to place Iobs on same scale as Iprofile.]
  Beq:                              118.57    [ = equivalent overall isotropic B factor on Fs.]

                                     B11      B22      B33      B23      B31      B12
  Baniso tensor:                  148.12   148.12    59.48     0.00     0.00     0.00

  NOTE: The Baniso tensor is the overall anisotropy tensor on Fs.

  Delta-B tensor:                  29.55    29.55   -59.10     0.00     0.00     0.00

  NOTE: The delta-B tensor is the overall anisotropy tensor on Fs after subtraction of Beq from its
  diagonal elements (so trace = 0).  Neither this nor its eigenvalues shown below is used further in
  any computation, including in anisotropy correction and deposition.

  Eigenvalues of overall anisotropy tensor (Ang.^2), eigenvalues after subtraction of smallest
  eigenvalue (as used in the anisotropy correction) and corresponding eigenvectors of the overall &
  anisotropy tensor as direction cosines relative to the orthonormal basis (standard PDB convention),
  and also in terms of reciprocal unit-cell vectors:

    Eigenvalue #1:          148.12    88.66   ( 1.0000,  0.0000,  0.0000)    0.894 a* - 0.447 b*
    Eigenvalue #2:          148.12    88.66   ( 0.0000,  1.0000,  0.0000)    b*
    Eigenvalue #3:           59.47     0.00   ( 0.0000,  0.0000,  1.0000)    c*

  The eigenvalues and eigenvectors of the overall B tensor are the squares of the lengths and the
  directions of the principal axes of the ellipsoid that represents the tensor.

  Delta-B eigenvalues:             29.55    29.55   -59.10

  The delta-B eigenvalues are the eigenvalues of the overall anisotropy tensor after subtraction of
  Beq (so sum = 0).

  Angle & axis of rotation of diffraction-limit ellipsoid relative to anisotropy tensor:

                                 0.00         0.0000   0.0000   1.0000

  Anisotropy ratio:             0.748    [ = (Emax - Emin) / Beq ]
  Fractional anisotropy:        0.407    [ = sqrt(1.5 Sum_i (E_i - Beq)^2 / Sum_i E_i^2) ]

  Eigenvalues & eigenvectors of redundancy anisotropy tensor:

                                 1.52         1.0000   0.0000   0.0000       0.894 a* - 0.447 b*
                                 1.52         0.0000   1.0000   0.0000       b*
                                 2.81         0.0000   0.0000   1.0000       c*

  Eigenvalues & eigenvectors of <I/sd(I)> anisotropy tensor:

                                 0.78         1.0000   0.0000   0.0000       0.894 a* - 0.447 b*
                                 0.78         0.0000   1.0000   0.0000       b*
                                 2.20         0.0000   0.0000   1.0000       c*

  Eigenvalues & eigenvectors of weighted CC_1/2 anisotropy tensor:

                                0.203         1.0000   0.0000   0.0000       0.894 a* - 0.447 b*
                                0.203         0.0000   1.0000   0.0000       b*
                                0.409         0.0000   0.0000   1.0000       c*

  Eigenvalues & eigenvectors of Z(weighted CC_1/2) anisotropy tensor:

                                 5.87         1.0000   0.0000   0.0000       0.894 a* - 0.447 b*
                                 5.87         0.0000   1.0000   0.0000       b*
                                11.72         0.0000   0.0000   1.0000       c*

  Eigenvalues & eigenvectors of <K-L divergence> anisotropy tensor:

                                0.377         1.0000   0.0000   0.0000       0.894 a* - 0.447 b*
                                0.377         0.0000   1.0000   0.0000       b*
                                0.962         0.0000   0.0000   1.0000       c*

  Eigenvalues & eigenvectors of <I/E[I]> anisotropy tensor:

                               2.64E-01         1.0000   0.0000   0.0000       0.894 a* - 0.447 b*
                               2.64E-01         0.0000   1.0000   0.0000       b*
                               5.06E-01         0.0000   0.0000   1.0000       c*

  Ranges of redundancy, local <I/sd(I)>, local weighted CC_1/2, local Z(weighted CC_1/2), local <K-L divergence>, local <I/E[I]> and D-W factor [= exp(-4 pi^2 s.Us)]:

                    Redund  ISmean  CChalf  ZChalf  KLdive  IEmean  DWfact
    0   Grey                             Unobservable*
    1   Blue                              Observable*
    2   Red|Pink:9       1    1.20  0.3001    8.00   0.479   0.921  0.0648
    3   Orange           2    2.94  0.7595   20.73   1.376   1.035  0.1436
    4   Yellow           4    6.03  0.8523   26.35   2.370   1.152  0.2776
    5   Green            5    9.86  0.8892   29.32   3.310   1.288  0.4683
    6   Cyan             6   13.88  0.9059   31.12   4.135   1.455  0.6893
    7   Magenta          8   14.99  0.9192   32.75   4.575   1.615  0.8854
    8   White            9   15.11  0.9264   33.72   4.902   1.764  0.9923

  * Refer to GLOSSARY for explanation of terminology.

  The fitted cut-off surface uses a different color scheme:
  Unmeasured points are blue (inside the surface) or cyan (outside).
  Unobserved points are red (in) or green (out).
  Observed points are orange (in) or white (out).
  The fitted surface is magenta.

  Anisotropic S/N ratio:  6.45        [ = max_h | exp(4 pi^2 s_h.delta(B)s_h) - 1 | <I_h/sd(I_h)> ]

  The 'anisotropic S/N ratio', unlike the 'anisotropy ratio' or the 'fractional' anisotropy shown
  above, in addition to the anisotropy of the B tensor, takes both the diffraction and the local mean
  I/sd(I) into account.

  GoF to ellipsoid (d*):  0.0211    Fraction of surface points fitted:  100.0%  (     2041 /     2041)

  Number of observed reflections inside surface:      13125
  Number of unobserved reflections inside surface:      993
  Number of observed reflections outside surface:         1

  Lowest cut-off diffraction limit:
              4.876 at reflection    4   26    2  in direction   0.152 a* + 0.986 b* + 0.076 c*

  Fraction of data inside cut-off surface:                           57.8%  (    13126 /    22725)

  Fraction of surface truncated by detector edges:                    1.5%  (       13 /      843)

  Fraction of total surface above threshold truncated by cusp(s):     0.0%  (        0 /      861)

  Estimated twin fraction from K-L divergence of observed acentric Z probability (before, after):  0.00  0.00

  Estimated twin fraction from K-L divergence of posterior acentric Z probability:                 0.00

  Estimated twin fraction from Britton histogram for k,h,-l operator (strong, all):                0.00  0.00

  Estimated twin fraction from Murray-Rust plot for k,h,-l operator (strong, all):                 0.00  0.01

  Estimated twin fraction from weighted Fisher & Sweet plot for k,h,-l operator:                   0.01

  Estimated twin fraction from K-L divergence of |delta-Z| probability for k,h,-l operator:        0.00

  Estimated twin fraction from K-L divergence of |H| probability for k,h,-l operator:              0.00

  Estimated twin fraction from Britton histogram for -h,-k,l operator (strong, all):               0.50  0.50

  Estimated twin fraction from Murray-Rust plot for -h,-k,l operator (strong, all):                0.01  0.02

  Estimated twin fraction from weighted Fisher & Sweet plot for -h,-k,l operator:                  0.01

  Estimated twin fraction from K-L divergence of |delta-Z| probability for -h,-k,l operator:       0.00

  Estimated twin fraction from K-L divergence of |H| probability for -h,-k,l operator:             0.01

  Estimated twin fraction from Britton histogram for -k,-h,-l operator (strong, all):              0.01  0.01

  Estimated twin fraction from Murray-Rust plot for -k,-h,-l operator (strong, all):               0.00  0.02

  Estimated twin fraction from weighted Fisher & Sweet plot for -k,-h,-l operator:                 0.02

  Estimated twin fraction from K-L divergence of |delta-Z| probability for -k,-h,-l operator:      0.00

  Estimated twin fraction from K-L divergence of |H| probability for -k,-h,-l operator:            0.00

  Estimated twin fraction from K-L divergence of unrelated acentric |delta-Z| probability:         0.00

  Padilla & Yeates L test for twinning, acentric moments of |L|:
  <|L|> (normal = .500; perfect twin = .375):  0.516
  <L^2> (normal = .333; perfect twin = .200):  0.347

  Estimated twin fraction from K-L divergence of |L| probability:                                  0.01
  ```

  ### - Normalised scores from a panoply of hemihedral twinning tests.

  For each test, the most likely hemihedral twin fraction based on the
  data is the one corresponding to the lowest normalised score
  (*i.e.* like the *R* value).  For all the distributional
  tests (*i.e.* those of the form P(*x*)), the raw score before
  normalisation is the Kullback-Leibler
  divergence from the theoretical to the observed probability
  density.  For the non-distributional Britton and Fisher & Sweet
  tests, it's the count of extra negative intensities obtained by
  detwinning the data using each possible twin fraction.  Note: click
  image to enlarge.
    
    
    
    
  **Reference**

---

- A complete list of the files in the downloaded tar archive
  '**ZwB5bFSwXQ4Y\_\_Q0.tgz**' follows:
    
    

  |  |  |  |  |  |  |  |  |  |  |  |  |  |  |  |  |
  | --- | --- | --- | --- | --- | --- | --- | --- | --- | --- | --- | --- | --- | --- | --- | --- |
  | Summary.html Summary file from server.|  |  |  |  |  |  |  |  |  |  |  |  |  |  | | --- | --- | --- | --- | --- | --- | --- | --- | --- | --- | --- | --- | --- | --- | | twintests.png Summary plot of all twinning tests.|  |  |  |  |  |  |  |  |  |  |  |  | | --- | --- | --- | --- | --- | --- | --- | --- | --- | --- | --- | --- | | |  |  |  |  |  |  |  |  |  |  |  | | --- | --- | --- | --- | --- | --- | --- | --- | --- | --- | --- | | Additional outputs from the server using the **'merged data' protocol**:| |  |  |  |  |  |  |  |  | | --- | --- | --- | --- | --- | --- | --- | --- | | -aniso-merged.log Log file from STARANISO, based on **unmerged** data scaled with an **isotropic** diffraction cut-off, then an **anisotropic** diffraction cut-off and anisotropy correction applied to the **merged** data.| -aniso-merged.res Results file from STARANISO.|  |  |  |  | | --- | --- | --- | --- | | -aniso-merged.mtz.bz2 Corrected MTZ file output by STARANISO.|  |  | | --- | --- | | -aniso-merged.mmcif.bz2 Corrected MTZ file output by STARANISO converted to mmCIF format for deposition, concatenated to the mmCIF datablock created from the STARANISO input MTZ file.  STARANISO server release v3.402 2-Oct-2025. | | | | | | | | | | | | | | | |
